# An immune-competent lung-on-a-chip for modelling the human severe influenza infection response

**DOI:** 10.1038/s41551-025-01491-9

**Published:** 2025-09-23

**Authors:** Rachel Ringquist, Eshant Bhatia, Paramita Chatterjee, Drishti Maniar, Zhou Fang, Page Franz, Liana Kramer, Delta Ghoshal, Neha Sonthi, Emma Downey, Joshua Canlas, Abigail Ochal, Savi Agarwal, Valeria Cuéllar, Grace Harrigan, Ahmet F. Coskun, Ankur Singh, Krishnendu Roy

**Affiliations:** 1 School of Chemical and Biomolecular Engineering, Georgia Institute of Technology, Atlanta, GA, USA.; 2 Woodruff School of Mechanical Engineering, Georgia Institute of Technology, Atlanta, GA, USA.; 3 Marcus Center for Therapeutic Cell Characterization and Manufacturing (MC3M), Georgia Institute of Technology, Atlanta, GA, USA.; 4 Parker H. Petit Institute for Bioengineering and Bioscience, Georgia Institute of Technology, Atlanta, GA, USA.; 5 Coulter Department of Biomedical Engineering, Georgia Institute of Technology and Emory University, Atlanta, GA, USA.; 6 School of Biological Sciences, Georgia Institute of Technology, Atlanta, GA, USA.; 7 School of Chemistry and Biochemistry, Georgia Institute of Technology, Atlanta, GA, USA.; 8 Scheller College of Business, Georgia Institute of Technology, Atlanta, GA, USA.; 9 Department of Biomedical Engineering, Vanderbilt University, Nashville, TN, USA.; 10 Department of Pathology, Microbiology, and Immunology, Vanderbilt University Medical Center, Nashville, TN, USA.; 11 Department of Chemical and Biomolecular Engineering, Vanderbilt University, Nashville, TN, USA.

## Abstract

Severe influenza affects 3–5 million people worldwide each year, resulting in more than 300,000 deaths annually. However, standard-of-care antiviral therapeutics have limited effectiveness in these patients. Current preclinical models of severe influenza fail to accurately recapitulate the human immune response to severe viral infection. Here we develop an immune-competent, microvascularized, human lung-on-a-chip device to model the small airways, successfully demonstrating the cytokine storm, immune cell activation, epithelial cell damage, and other cellular- and tissue-level human immune responses to severe H1N1 infection. We find that interleukin-1β and tumour necrosis factor-α play opposing roles in the initiation and regulation of the cytokine storm associated with severe influenza. Furthermore, we discover the critical stromal–immune CXCL12–CXCR4 interaction and its role in immune response to infection. Our results underscore the importance of stromal cells and immune cells in microphysiological models of severe lung disease, describing a scalable model for severe influenza research. We expect the immune-competent human lung-on-a-chip device to enable critical discoveries in respiratory host–pathogen interactions, therapeutic side effects, vaccine potency evaluation, and crosstalk between systemic and mucosal immunity in human lung.

Influenza A affects nearly 1 billion people per year, producing a broad spectrum of disease severity and resulting in 290,000–600,000 deaths worldwide annually^1–4^. Severe viral infection and replication in airway epithelial cells can induce the production of cytokines and chemokines in response to both cell-intrinsic and extrinsic signals. These inflammatory signals drive the presence of monocytes as the predominant inflammatory cells in the early stages and recruit neutrophils with increasing degrees of epithelial necrosis in severe disease^3^. Innate signals are responsible for mounting the initial immune response and instruct the adaptive immune system to control and eliminate the infection through immunoglobulin-mediated blocking of viral spread and T cell-mediated elimination of virus-infected cells^5,6^. The T cell response in draining lymph nodes is further orchestrated by lung tissue-resident immune and stromal cells^4^. A strong local immune response may rapidly clear the virus; however, when unchecked, it can also cause extensive collateral tissue damage and loss of cellular function. It is challenging to determine if and how a host can balance between clearance of the pathogen and maintenance of tissue function, especially in cases with high viral titre and severe disease. Understanding the immune–stromal crosstalk and key drivers of the lung immune response could address the inefficacies of current treatment options against severe influenza cases.

Our understanding of disease severity outcomes in H1N1 infection and the development of targeted therapeutics is hindered by a lack of preclinical models that can determine the immune correlates of protection, enable the study of complex interactions between structural cells of the lung and resident immune cells, identify viable therapeutic candidates and demonstrate off-target effects. Currently, H1N1 infection and disease severity studies are based on animal models, post-mortem analysis of patients, or staged clinical trials. Therapeutic development is also centred around high-throughput screening using 2D cell culture and animal models. However, only around 1% of therapeutic candidates make it to clinical trials, and 12% of therapies that enter clinical trials translate to the market^7^. This highlights the discrepancy between therapeutic performance in current state-of-the-art preclinical models and actual clinical response. Importantly, there are also physiological differences between species for severe influenza infection that prevent results from in vivo mouse models from being directly translatable to humans. For example, in addition to differences in virus-binding receptors, there are substantial differences in lung vasculature and relative airway size between mice and humans, which makes it difficult to translate results from deep lung studies in mice to humans^8,9^. Alternative ferret models that share lung pathology and cellular receptors to humans^10^ are costly and suffer from a shortage of ferret-specific reagents, such as antibodies, for the analysis of immune responses to influenza virus infection and vaccination^11^. Therefore, while the ferret model answers some of the physiological limitations of mouse models, it invokes a new set of logistical and often cost-preventative issues.

To overcome the aforementioned barriers and develop a severe influenza model of the human lung, there is a need for a physiologically relevant in vitro model of human lung tissue that is capable of acquiring distinct activation states of structural cells (epithelial, stromal and endothelial) and that integrates the activation signals at both the cellular and tissue levels for innate and adaptive host immune responses. Unfortunately, no such in vitro models exist as current lung tissue models^12–21^ mostly fail to capture the complex lung microvasculature and the full repertoire of immune cells to accurately evaluate the spectrum of immune–stromal interactions and the systems immunology of the virally infected lung. Canonical lung-on-a-chip (LOC) models use the bilayer system^22^, serving to recapitulate the alveolar–capillary interface with a monolayer of alveolar epithelial cells separated by a membrane from an endothelial cell monolayer^22^. Although these simplified models have offered valuable insights into the role of lung mechanical forces in viral replication and mutagenesis, as well as the response of endothelial monolayers to epithelial infection, they lack critical immune components necessary to study the immune response to viral infection and exclude the interstitial extracellular matrix (ECM) and stromal components of the lung^23–27^.

A more advanced system^18^, consists of a microfluidic device mimicking the bronchial airway, lined with human bronchial epithelial and pulmonary endothelial cells, to effectively mimic viral infections. This system allows the study of variations in viral virulence, cytokine production and the recruitment of primary neutrophils from the circulating channel. However, a critical limitation of this study is that it largely focuses on neutrophils and does not present the complex spectrum of tissue-resident and circulatory immune cells, which are critical for defence against pathogens. These immune cells include, but are not limited to, airway macrophages (AMs), interstitial macrophages (IMs), interstitial dendritic cells (DCs) and peripheral blood mononuclear cells (PBMCs). To the best of our knowledge, no LOC studies have successfully demonstrated immune–immune and immune–stromal crosstalk or the impact of a diverse spectrum of immune cells on the extracellular environment of the lung in severe H1N1 infection. Although studies have shown that low infectivity (multiplicity of infection (MOI) 0.001–0.1) can still damage the bronchial epithelial cells from the upper respiratory tract^18^, it remains unclear whether such systems are able to model severe influenza and induce an immune response similar to humans, as the progression of severe influenza often entails the migration of the disease into the lower respiratory tract, where the distribution of sialic acid receptors varies significantly from that of the upper airways^28–33^. These critical gaps emphasize the importance of developing a fully immunocompetent small airway LOC model to reproduce the antiviral inflammatory immune response and illustrate the intricate signalling cascade involved in severe influenza infection.

Here we bioengineered a human lung tissue model that represents small airway structures with tissue-resident and circulatory immune cells, including AMs and IMs, DCs, granulocytes, neutrophils, T cells, B cells and natural killer (NK) cells. Incorporating granulocytes required the use of whole blood as PBMCs exclude the polymorphonuclear fraction of white blood cells, which are key mediators of immune responses. While endothelial and stromal cells can become activated upon encountering a pathogen, the pro-inflammatory cytokines and interferons secreted by recruited immune cells exacerbate this activation. With this platform, we demonstrate that a conventional LOC model of the small airway, which lacks immune cells, induces a limited cytokine response to severe influenza infection. By contrast, LOCs containing tissue-resident macrophages induce a significant cytokine response in the airway layer of the device, mimicking bronchoalveolar lavage (BAL) fluid of patients. However, the full array of tissue-resident and circulatory immune cells is necessary to elicit a significant airway and interstitial cytokine storm in response to severe infection. Through single-cell RNA sequencing (scRNA-seq), we identify key pathways that become dysregulated in the case of severe influenza, specifically the interleukin (IL)-1β pathway, the tumour necrosis factor (TNF)-α pathway and the CXCL12–CXCR4 axis, driven by fibroblast-secreted CXCL12. We find that inhibition of IL-1β completely ameliorates the observed cytokine storm, whereas TNF-α appears to be a critical regulator, as its inhibition results in a highly increased inflammatory response. In addition, inhibition of the CXCR4 axis plays a critical role in the influx of immune cells into inflamed tissue, modulating the inflammatory landscape by decreasing levels of pro-inflammatory cytokines while increasing antiviral cytokines such as interferons. Thus, we show that the inclusion of tissue-resident and circulatory immune cells, as well as stromal cells within our LOC model, allows us to recapitulate hallmarks of disease states such as severe influenza and sheds light on potential therapeutic avenues targeting immune dysregulation.

## Results

### Immune-competent LOC mimics small airway cell composition

**Fig. 1 |:**
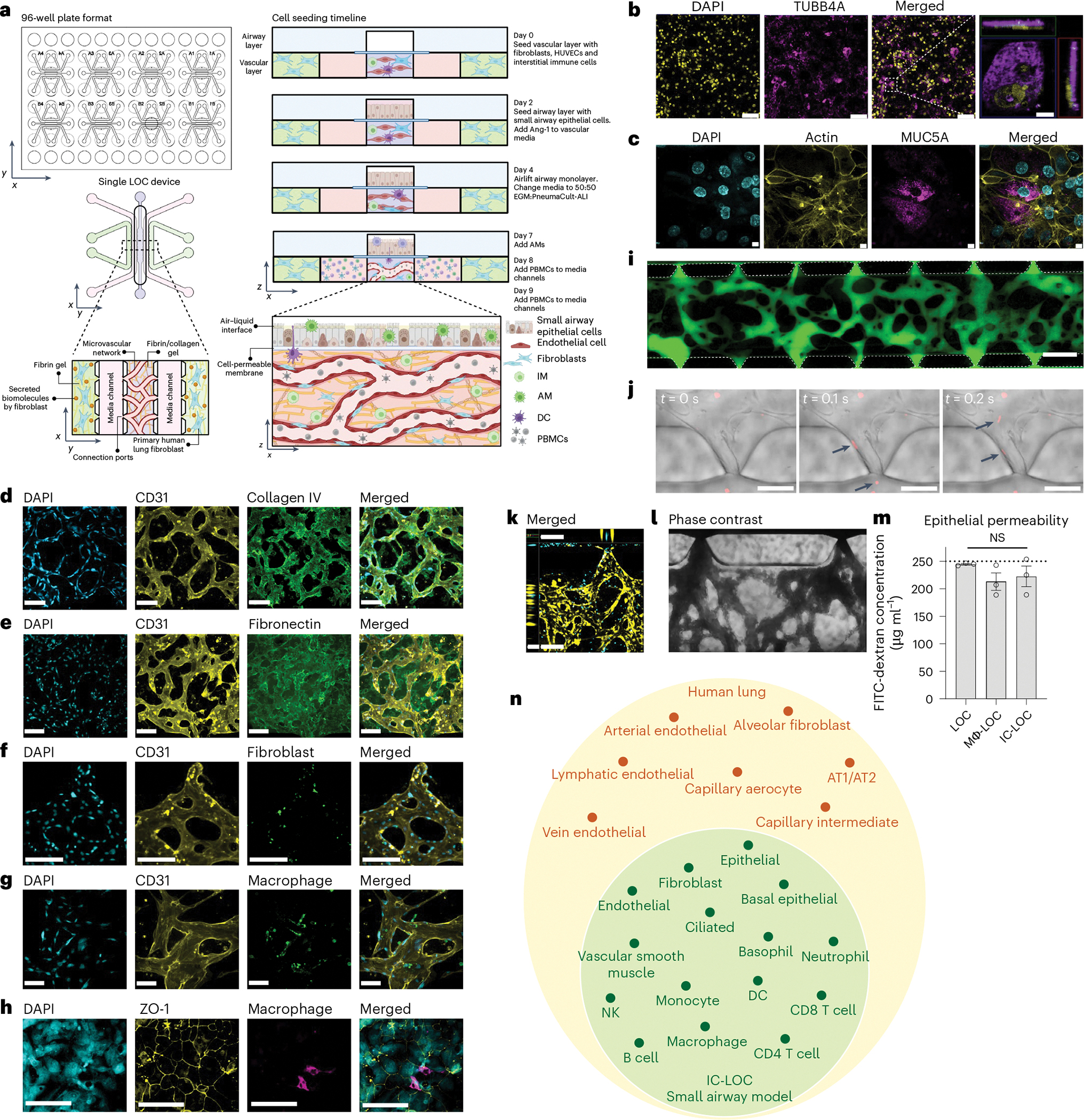
An immune-competent, microvascularized, human LOC device in a 96-well format. **a**, Schematic of the 96-well plate format of the LOC device, showing the 2 × 4 grid formatted to fit on a bottomless 96-well plate, including a zoomed-in view of a single device and the cellular composition of each channel and the device seeding timeline, created using BioRender (https://BioRender.com/7wk1dzr). **b**,**c**, Representative images of the airway epithelium in the LOC device, showing DAPI in yellow and ciliated cell marker TUBB4A in magenta (scale bars, 100 μm), with inset showing TUBB4A signal situated at the top surface of the cell (scale bar, 10 μm) (**b**), as well as goblet cell marker Mucin5AC (magenta) and actin (yellow) (**c**). Scale bars, 11 μm. **d**, ECM staining of collagen IV (green) surrounding the formed vessels (CD31, yellow). Scale bars, 160 μm. **e**, Additional ECM staining of fibronectin (green) in the interstitial space along with CD31 (yellow) staining of the vessels. Scale bars, 160 μm. **f**, Incorporation of labelled interstitial fibroblasts (green) with vasculature (CD31, yellow). Scale bars, 160 μm. **g**, Incorporation of IMs (green) with vasculature (CD31, yellow). Scale bars, 80 μm. **h**, Representative images of the epithelium (ZO-1, yellow) with AMs (CD68, magenta). Scale bars, 100 μm. **i**, The microvascular network perfused with 70 kDa FITC-dextran. Scale bar, 500 μm. **j**, Timeframe images (Δ*t* = 100 ms) of labelled PBMCs (red, arrows) entering the microvascular network through open lumens between ports of the device. **k**, Labelled PBMCs (cyan) perfused through the microvascular network (yellow), showing PBMCs entering through an open lumen. Scale bar, *x*-direction 160 μm, *z*-direction 50 μm. **l**, Bright-field image of whole blood perfused through the microvasculature. Scale bar, 160 μm. **m**, 10 kDa FITC-dextran permeability of the epithelium in LOC, Mϕ-LOC and IC-LOC devices, quantified by the airway concentration of FITC-dextran at the assay endpoint (2 h incubation). The dotted line represents the starting concentration of 250 μg ml^−1^. *N* = 3 independent devices, with statistical significance determined via ordinary one-way ANOVA followed by Tukey’s multiple comparisons test. NS, not signficant. Data are presented as mean values ± s.d. **n**, A graph depicting the major cell types observed in the IC-LOC and the human lung, as determined via scRNA-seq.

The immune-competent LOC model of the small airway comprises a three-dimensional (3D), perfusable microvascular network underneath a mature, differentiated airway epithelium at an air–liquid interface (ALI). The 3D microvascular network provides a unique advantage in that it is comprised of both endothelial cells and fibroblasts in a hydrogel matrix, in which interstitial immune cells can be incorporated. The perfusable microvascular network introduces circulating immune cells, which have the potential to migrate into the vascular network, extravasate and transmigrate in response to a stimulated epithelium. We modified our recently reported microphysiological human LOC device^34^ to include multiple tissue-resident and circulatory immune cell types (Fig. 1a and Supplementary Fig. 1). The modified device incorporates a monolayer of small airway epithelial cells (SAECs) at ALI separated by a cell-permeable 5.0 μm pore size polyester track etched (PETE) membrane from a 3D perfusable microvascular network to allow for immune cell migration between the two compartments. The membrane pore size was optimized to allow for active immune cell migration across the membrane in response to a chemoattractant (Supplementary Fig. 2a). A schematic of the device design, as well as the cell seeding timeline for the device, is shown in Fig. 1a. To validate that the 5.0 μm pore size membrane could support the formation of a mature, differentiated airway epithelium in the LOC device, we evaluated the expression of ciliated cell marker beta-tubulin (TUBB4A) (Fig. 1b), as well as goblet cell marker mucin 5AC (MUC5AC, Fig. 1c), indicative of a differentiated epithelium in the airway layer of the LOC. As expected, we observed cilia to be situated on the top surface of the epithelial cells within the airway layer of the lung chip devices (Fig. 1b, inset). To investigate the formation of the interstitial layer underlying the airway epithelium, we performed confocal imaging and observed networks of CD31^+^ human umbilical vein endothelial cells (HUVECs) (Fig. 1d,e). ECM staining showed high levels of secreted collagen IV forming a basement-like membrane around the vessels (CD31) (Fig. 1d) as well as diffuse fibronectin in the interstitial regions between the vessels (Fig. 1e). These observations align with what has been shown in the human lung, with collagen IV primarily found in basement membranes and fibronectin largely existing in the interstitial matrix^35–37^. Confocal imaging further confirmed the presence of the interstitial fibroblasts interspersed within the CD31^+^ vascular network (Fig. 1f). These fibroblasts are perivascular and function as stromal support, with a distinct role from those in the outer channels where the fibroblasts support the formation of vasculature through the secretion of growth factors.

To determine the role of AMs and IMs on lung structure and function, we incorporated blood monocyte-derived macrophages into the LOC, denoted Mϕ-LOC. Monocytes were isolated from whole blood and differentiated with macrophage colony-stimulating factor (M-CSF) for at least 7 days before incorporation within the LOC. Media composition was optimized, and IMs were successfully incorporated into the interstitial vascular layer on day 0 at a ratio of 1:10 with the HUVECs, as shown in Fig. 1g and Supplementary Fig. 2b. By pre-labelling macrophages with CellTracker Green, we found that the IMs primarily localized perivascularly (Fig. 1g). On day 7, after establishing ALI, macrophages were also incorporated into the airway layer of the Mϕ-LOC at a concentration of 1 million cells per ml. These AMs were successfully maintained in the epithelium until at least day 12 without disrupting the integrity of the epithelial monolayer, as demonstrated by intact ZO-1 staining near CD68^+^ macrophages (Fig. 1h).

**Extended Data Fig. 1 |:**
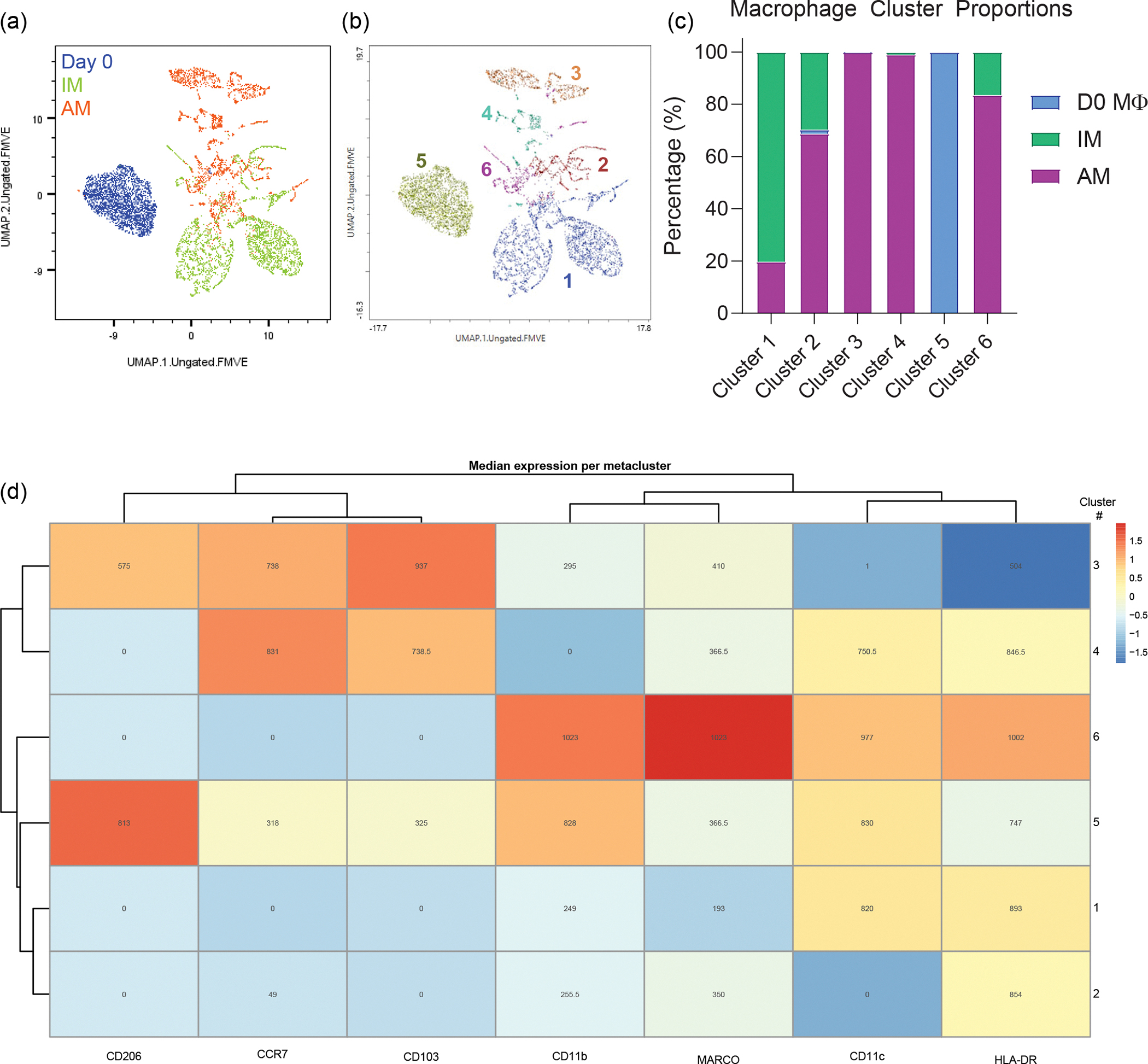
Culture within the lung-on-chip device induces macrophage heterogeneity. **(a)** Flow cytometry analysis of Day 0 macrophages (pre-device) and interstitial macrophages (IM) and airway macrophages (AM) from the device. IMs were prelabelled with CellTracker Green and AMs were prelabelled with CellTrace Yellow, such that the populations could be distinguished within the IC-LOC samples. Expression of markers CD206, CCR7, CD103, CD11b, MARCO, CD11c, and HLA-DR was used to map the cells onto 2D space using the UMAP plugin in FlowJo. **(b)** The FlowSOM plugin in FlowJo was used to cluster the cells according to their expression levels of the aforementioned markers, resulting in 6 distinct clusters. **(c)** The composition of each cluster is shown, with Cluster 5 comprised exclusively of Day 0 macrophages, Clusters 3 and 4 comprised exclusively of airway macrophages, and Clusters 1, 2 and 6 containing a mixture of cells. **(d)** A heatmap illustrating the mean fluorescence intensity of each of the given markers in each of the clusters, with the color showing the standardized levels of expression and the true MFI values also shown. All groups are comprised of N = 3 samples. Four IC-LOC devices were pooled for each AM/IM sample, and all devices used the same immune cell donor.

Furthermore, we found that macrophages adopted distinct phenotypes depending upon their location, interstitial or airway, within the device. Utilizing the UMAP and FlowSOM plugins in FlowJo version 10.8 to analyse flow cytometry data of the expression of markers CD11b, CD11c, CD206, HLA-DR, MARCO, CCR7 and CD103, we observed that day 0 (pre-device) macrophages, IMs from devices and AMs from devices each clustered separately (Extended Data Fig. 1a–c). To accurately identify IMs and AMs from the devices, IMs were pre-labelled with CellTracker Green, and AMs were pre-labelled with CellTrace Yellow before incorporation into the device. Before culture within the device, the macrophage population was largely homogeneous, with high levels of CD206 and CD11b (Extended Data Fig. 1d). Meanwhile, the IMs and AMs clustered into five separate clusters. While there was some overlap between the airway and interstitial populations, as expected, the IMs and AMs largely clustered separately and demonstrated a high level of heterogeneity after culture within the device (Extended Data Fig. 1d). The IMs retained relatively low expression of markers MARCO, CCR7, CD206 and CD103. By contrast, the AM populations expressed high levels of these markers, depending upon cluster. Furthermore, we observed an overall increase in CD11c and HLA-DR expression on AMs compared with those in pre-device macrophages (day 0). MARCO, CD11c and HLA-DR represent important mediators of cell adhesion, phagocytosis and antigen presentation in AM populations. Thus, their increase upon culture within the Mϕ-LOC demonstrates the physiologically relevant influence of the Mϕ-LOC microenvironment on cellular phenotype^19,38–42^. Thus, both IM and AM populations can be represented within the LOC, which is herein referred to as the Mϕ-LOC.

**Extended Data Fig. 2 |:**
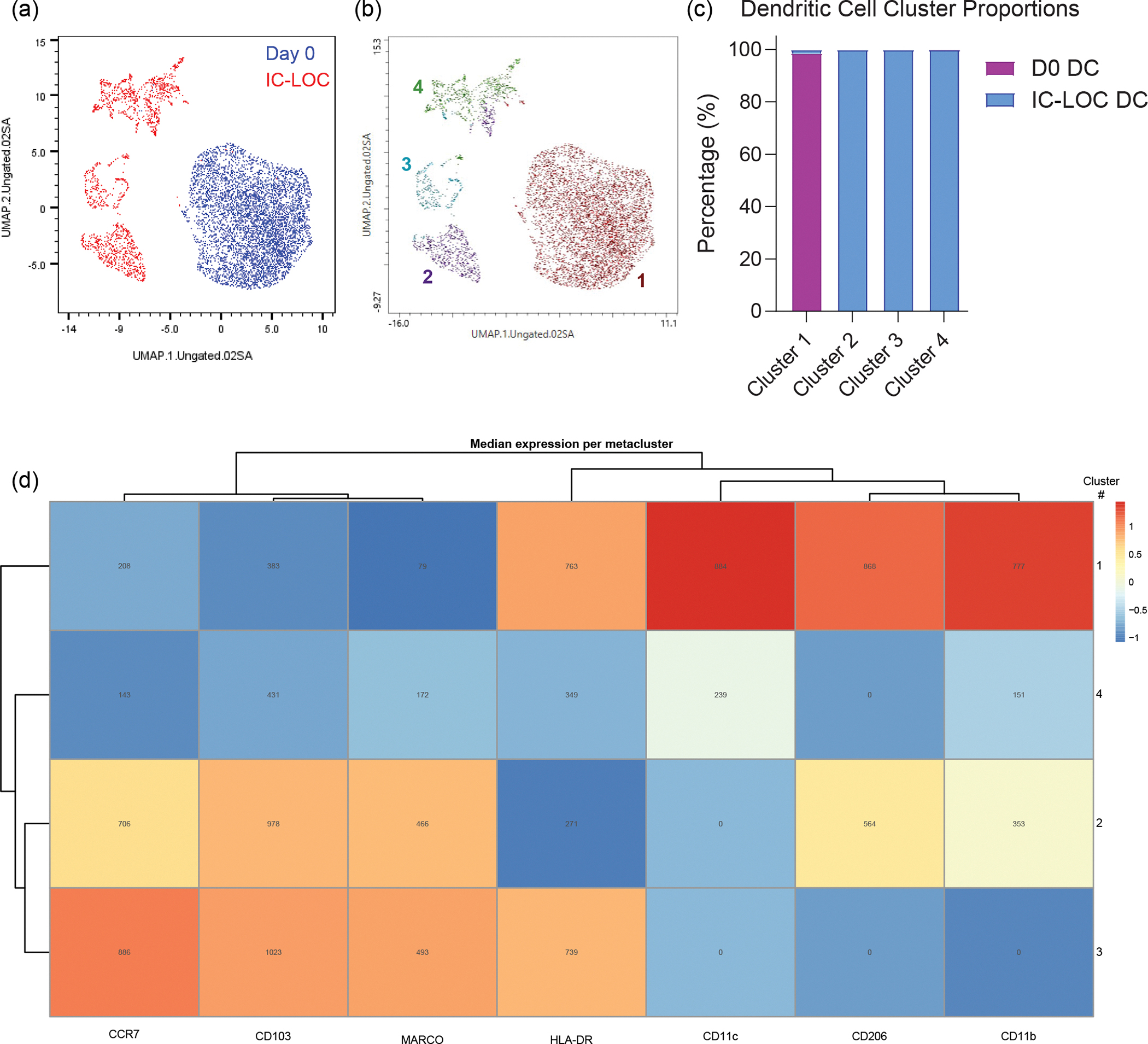
Culture within the lung-on-chip device induces dendritic cell heterogeneity. **(a)** Flow cytometry analysis of Day 0 dendritic cells (DCs) (pre-device) and IC-LOC DCs from the device. DCs were pre-labelled with CellTrace Violet prior to incorporation into the IC-LOC to allow them to be distinguished at the endpoint. Expression of markers CD206, CCR7, CD103, CD11b, MARCO, CD11c, and HLA-DR was used to map the cells onto 2D space using the UMAP plugin in FlowJo. **(b)** The FlowSOM plugin in FlowJo was used to cluster the cells according to their expression levels of the aforementioned markers, resulting in 4 distinct clusters. **(c)** The composition of each cluster is shown, with Cluster 1 comprised exclusively of Day 0 DCs and Clusters 2, 3, and 4 comprised exclusively of IC-LOC DCs. **(d)** A heatmap illustrating the mean fluorescence intensity of each of the given markers in each of the clusters, color showing the standardized levels of expression and the true MFI values also shown. All groups are comprised of N = 3 samples. Four IC-LOC devices were pooled for each AM/IM sample, and all devices used the same immune cell donor.

We then built on the Mϕ-LOC to include additional immune populations to enhance the physiological relevance of the device. Specifically, within the interstitial compartment, we incorporated interstitial DCs in a similar manner to IMs, at a ratio of 1:10 with the HUVECs. DCs were differentiated from blood monocytes using IL-4 and granulocyte-macrophage colony-stimulating factor (GM-CSF) and were donor-matched to the incorporated macrophages. Lung DCs are critical in the response to foreign pathogens and the initiation of the adaptive immune response^43^. Here we found that the day 0 (pre-device) DC population was largely homogeneous and clustered separately from the more heterogeneous post-device DCs (Extended Data Fig. 2a–c). Before culture within the device, the DC population expressed high levels of CD206, CD11b and CD11c and clustered into a single population. However, after culture within the device, we observed an increase in heterogeneity and an increase in CD103, CCR7 and MARCO expression (Extended Data Fig. 2d). CD103^+^ DCs are canonically associated with the lung epithelium and are critical for the antiviral T cell response, and thus it is encouraging that we can recapitulate this phenotype within the chip^44^.

**Extended Data Fig. 3 |:**
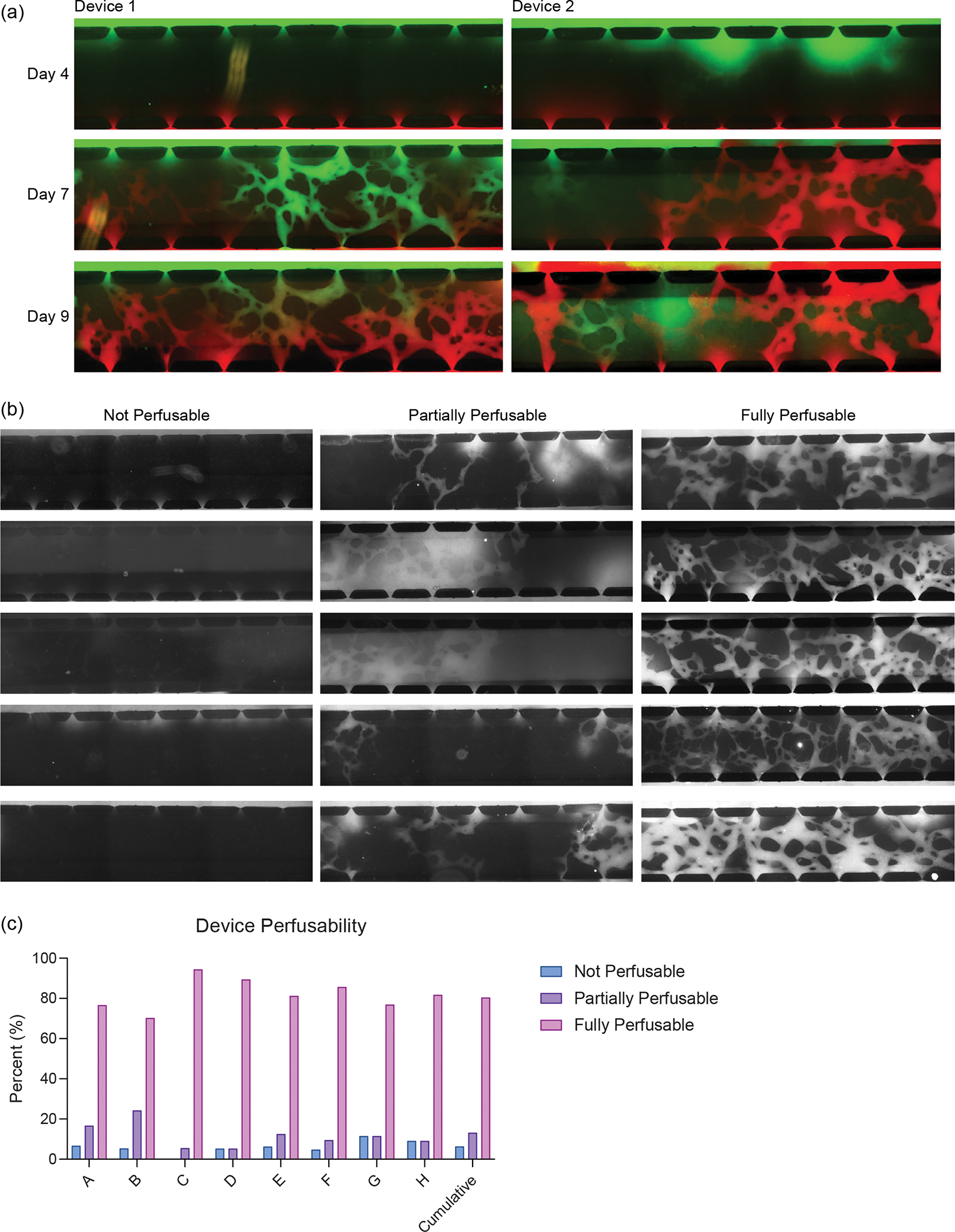
Perfusability and reproducibility of the vascular network in the LOC. **(a)** Representative images shown from two independent devices, imaged sequentially over 9 days to identify the timepoint at which the network was fully perfusable. Perfusability was evaluated using 70 kDa FITC- and TRITC-dextran. **(b)** Representative images of 70 kDa FITC-Dextran perfusion in lung chip devices, split into three categories based on perfusability. “Not perfusable” denotes devices in which 0 of the 7 ports on either side of the central channel are open. “Partially perfusable” denotes devices in which more than 0 but less than 6 of the 7 ports on either side of the central channel are open. “Fully perfusable” denotes devices in which at least 6 of the 7 ports on either side of the central channel are open. **(c)** Quantification of the percentage of devices in each category of perfusion from 8 independent experiments, labelled A-H, as well as the cumulative overall percentages across all 8 experiments.

The severity of influenza is dependent on the host’s both innate and adaptive immunity^45^. Thus, to mimic this, we aimed to incorporate circulatory immune cells into the model. To this end, we first evaluated the timeline of perfusability of the vascular network within the lung chip. We observed the formation of a fully perfusable network within the device by day 9 as observed by perfusion of 70 kDa FITC-dextran (Fig. 1i and Extended Data Fig. 3a). To address the reliability and repeatability of the perfusable network formation, we characterized the dextran perfusability of devices from 8 independent experiments, ranging from 24 to 48 devices per experiment. For this analysis, we characterized devices as either ‘not perfusable’ (0/7 open ports), ‘partially perfusable’ (>0/7 but <6/7 open ports) or ‘fully perfusable’ (≥6/7 open ports). Through this, we found that across 8 independent experiments, 80.4% of devices (152 devices) were fully perfusable, 13.2% (25 devices) were partially perfusable, and 6.3% (12 devices) were not perfusable. Across the 8 experiments, the percentage of fully perfusable devices ranged from 70.3% to 94.4% (Extended Data Fig. 3b,c). After confirming the perfusable vasculature, on day 9, we added PBMCs or red blood cell (RBC)-depleted whole blood to the media channels to perfuse through the microvascular network (Fig. 1j–l). We observed the entrance of circulatory immune cells (PBMCs; Fig. 1j,k) or whole blood (Fig. 1l) into the central channel through the open lumens of the microvasculature. All immune cells (macrophages, DCs and PBMCs) used in the model were obtained from the same single donor to mitigate non-self-immune reactions as much as possible without a fully autologous system and to ensure reproducibility by avoiding donor-dependent variances. The LOC with incorporated tissue-resident (AM, IM and DC) and circulatory immune cells was coined the immune-competent LOC (IC-LOC).

**Extended Data Fig. 4 |:**
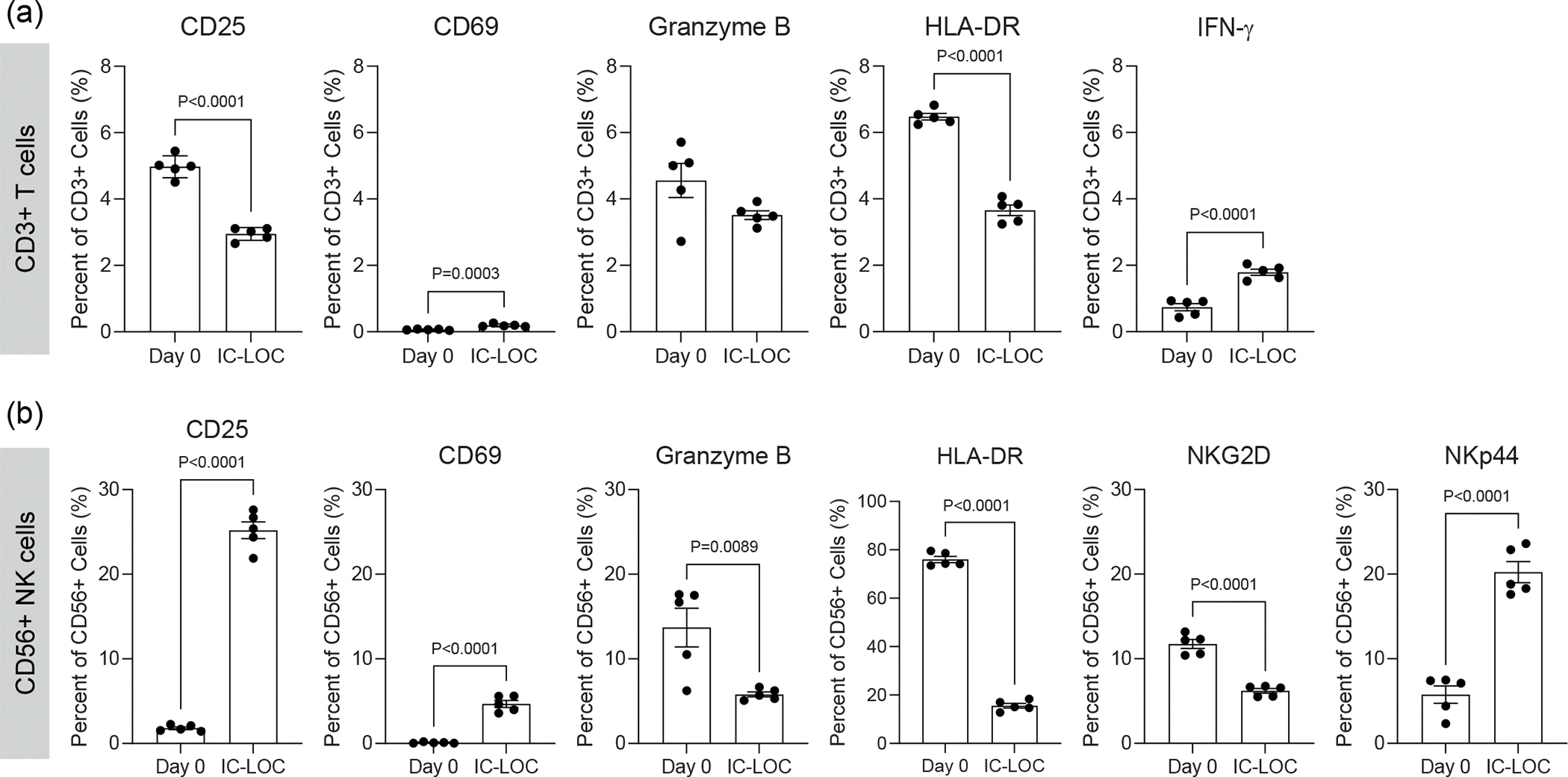
Culture within the IC-LOC does not induce substantial immune cell activation. **(a)** Flow cytometry analysis of activation markers on CD3 + T and **(b)** CD56 + NK cell populations from PBMCs pre- and post- culture within the device, designated D0 PBMCs and IC-LOC, −H1N1, respectively. N = 5 samples, with two IC-LOC devices from the same immune cell donor pooled per sample for the IC-LOC samples. Statistical significance determined via two-tailed unpaired t-test, with p-values shown on graph and error bars showing S.E.M.

The use of RBC-depleted whole blood markedly deviates from current approaches that use only PBMCs, thereby excluding the polymorphonuclear fraction of white blood cells, key mediators of immune responses. Especially in the context of viral infection, where neutrophils and other granulocytes are key responders, incorporating these cells is critical. The circulating immune populations within the devices were maintained for up to 72 h with viability of >90% in the circulating fraction and >75% in the extravasated interstitial fraction (Supplementary Fig. 3). In addition, we did not observe any substantial activation of the T cells owing to culture within the device, as evidenced by very low levels of activation markers CD25, CD69, HLA-DR, granzyme B, interferon (IFN)-γ, NKp44 and NKG2D after 72 h within the device, comparable to that of the day 0 (pre-device) PBMCs (Extended Data Fig. 4a). However, we did observe mild activation of NK cells upon 72 h culture within the chip, with an increase in select activation markers such as CD25, CD69 and NKp44 but a decrease in others such as HLA-DR, granzyme B and NKG2D (Extended Data Fig. 4b).

To examine the integrity of the airway barrier in the presence of immune cells, we performed an FITC-dextran permeability assay by adding 250 μg ml^−1^ of 10 kDa FITC-dextran in PBS to the airway chamber. We observed that the concentration of the FITC-dextran did not significantly change after 2 h of incubation, irrespective of LOC, Mϕ-LOC and IC-LOC (Fig. 1m), indicating the formation of a tight epithelial barrier. The choice of 10 kDa dextran was based on previous publications^46^. In addition, we observed no significant changes in the vasculature characteristics between the three models (LOC, Mϕ-LOC and IC-LOC) (Supplementary Fig. 4).

To further understand the cellular heterogeneity of the IC-LOC system, we performed scRNA-seq on devices with incorporated AMs, IMs, DCs and RBC-depleted whole blood and analysed the resulting transcriptomic data using the Seurat package in R^47–50^. Through scRNA-seq analysis of our IC-LOC and a published human lung atlas scRNA-seq dataset^51^, we found that we were able to recapitulate the majority of the cell types found in the in vivo lung that correspond to those of the small airway (Fig. 1n and Supplementary Fig. 5), as our focus was specifically on the small airway in the current study. Specifically, when evaluating the resulting clusters from the integrated dataset, we found that the majority were shared between the IC-LOC and the human lung, including T cell, B cell, NK cell, DC, macrophage, monocyte, neutrophil, epithelial cell, fibroblast and endothelial cell clusters. As expected, the cell types that were exclusively found in the in vivo human lung dataset corresponded primarily to alveolar-associated cells, including alveolar epithelial cells, alveolar fibroblasts, lymphatic endothelial cells, and specialized arterial and vein endothelial cells. When considering the overall comparison, we found that the IC-LOC recapitulates the cell types expected in the in vivo small airways in human lungs. While most cell types were recapitulated, we observe differences in proportions relative to the human lung dataset (Supplementary Fig. 5). This may be explained by the incorporation of both the central channel (interstitium and airway) and circulatory cells from the media channels in our IC-LOC dataset. By contrast, the human lung dataset would differ in the proportion of blood cells and includes regions of the lung outside of the small airways. Furthermore, while the overarching cell types are represented, more in-depth sequencing analysis is required to determine whether phenotypic subpopulations are maintained. Regardless, we find that our IC-LOC models are among the most complete in vitro representations of the small airway regions of the human lung thus far.

### Establishing severe influenza in the LOC

**Fig. 2 |:**
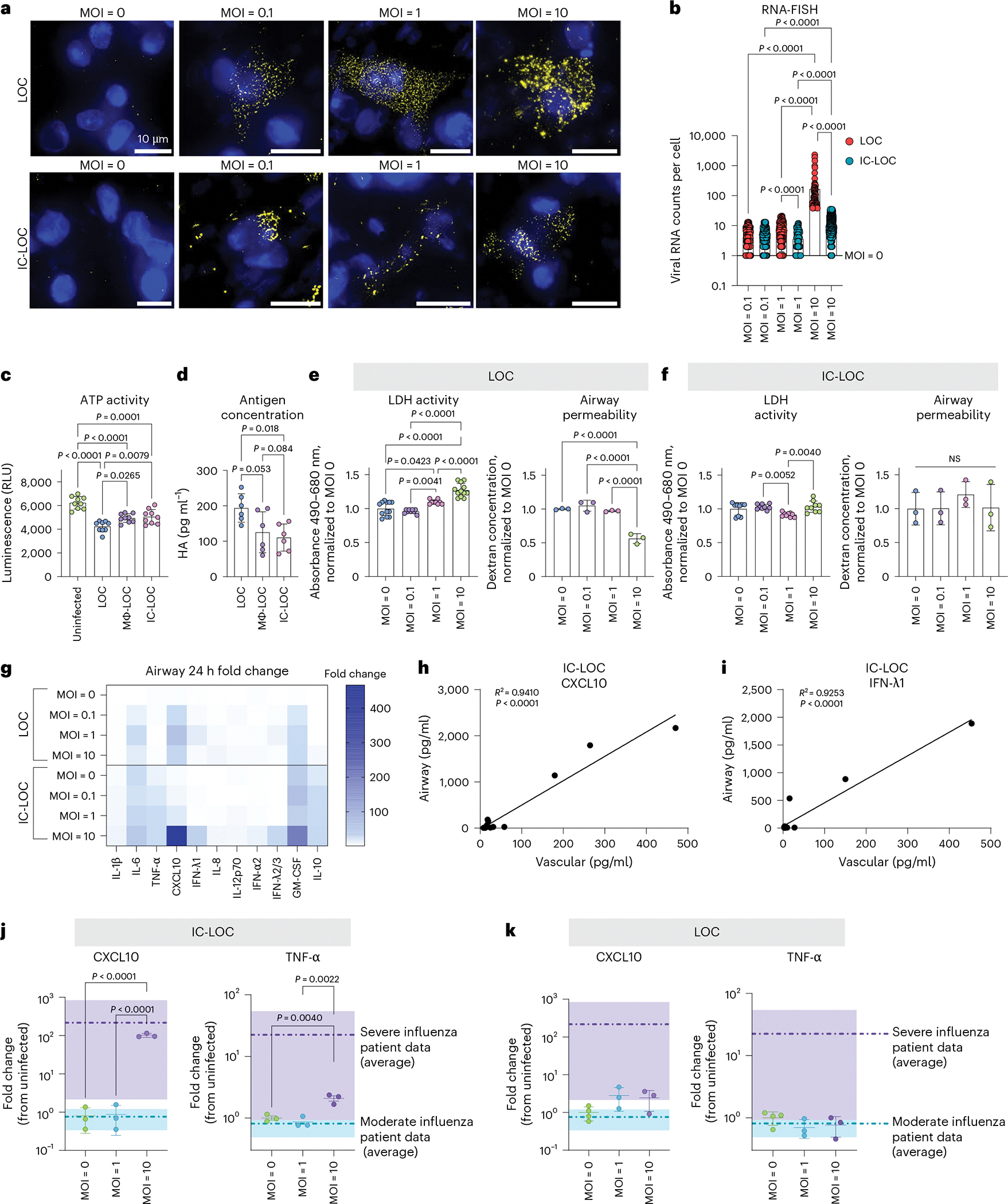
Establishing severe influenza on chip. **a**, Representative RNA-FISH images of the epithelial layer of the device, showing nuclei in blue and viral H1N1 RNA in yellow for the LOC and IC-LOC at MOI = 0, 0.1, 1 and 10. **b**, Quantification of the viral RNA counts per cell, as determined via RNA-FISH. *N* = 3 independent devices. Statistical significance determined via one-way ANOVA with Kruskal–Wallis test, and *P* values are shown. **c**, Relative quantification of infectious viral titre via a ViralTox Glo assay. Relative luminescence (RLU) corresponds to ATP activity of H1N1-susceptible MDCK cells cultured with the cell lysis of uninfected devices or LOC, Mϕ-LOC or IC-LOC devices infected at MOI = 10. *N* = 10 independent devices. **d**, Quantification of antigen (HA) concentration in the cell lysis of LOC, Mϕ-LOC or IC-LOC devices infected at MOI = 10. *N* = 6 independent devices. **e**, Quantification of epithelial damage via LDH activity and airway permeability in the LOC in response to H1N1 infection at MOI. *N* = 8 independent devices. **f**, Quantification of epithelial damage via LDH activity and airway permeability in the IC-LOC in response to H1N1 infection at varying MOIs. *N* = 3 independent devices. **g**, LEGENDplex analysis of the cytokine release profiles in the LOC and IC-LOC 24 hpi at varying MOIs. Heat map shows the fold change from time zero (pre-infection). *N* = 4 independent devices. **h**,**i**, Simple linear regression correlation of airway and vascular levels of CXCL10 (**h**) and IFN-λ1 (**i**) in the IC-LOC. *N* = 14 independent devices. **j**,**k**, Comparison of cytokine release profiles in the airway layer of the IC-LOC (**j**) and LOC (**k**) to that of BAL fluid from patients with moderate and severe influenza. Fold change from uninfected/healthy is shown. Purple bands denote the range of patient data with severe influenza, while teal bands denote the range of patients with moderate influenza. *N* = 3 independent devices. All immune-competent devices use cells from the same immune cell donor. All statistical significance determined via one-way ANOVA followed by Tukey’s multiple comparisons test unless otherwise noted. Data are presented as mean values ± s.d.

To develop a severe influenza model within the LOC, the epithelium of LOC, Mϕ-LOC and IC-LOC devices were infected with live H1N1 (A/PR/08/34). We evaluated a series of MOIs ranging from 0.1 to 10 to determine the MOI that best recapitulated select aspects of severe influenza. Infection was started on day 9, after establishing epithelial ALI (day 4) and introducing AMs (day 7) with circulatory immune cell populations (day 9). We utilized single-molecule RNA fluorescence in situ hybridization (RNA-FISH) imaging to detect and quantify intracellular viral RNA levels at the single cell level at 72 h post-infection (hpi) in the LOC and IC-LOC at varying MOIs (Fig. 2a). We observed a significant spike in viral RNA counts per cell at MOI 10 in both LOC and IC-LOC devices, averaging nearly 100 viral RNA counts per cell in the LOC epithelium, with IC-LOC resulting in comparatively lower viral loads (Fig. 2b).

To complement the RNA-FISH observations, we further quantified viral levels in devices infected at MOI 10 using a Viral ToxGlo assay (Promega) and hemagglutinin-specific (HA) enzyme-linked immunosorbent assays (ELISAs) (Fig. 2c,d). The Viral ToxGlo assay involved culturing H1N1-susceptible Madin–Darby canine kidney (MDCK) cells with the collected media and cell lysis from infected devices, thus pooling all intra- and extracellular virions from an infected device. In this assay, decreased ATP activity corresponds to increased viral-induced cell death^52^. We observed significantly more viral-induced cell death from LOC samples than Mϕ-LOC and IC-LOC samples, indicating a higher titre of infectious virus in the LOC devices (Fig. 2c). Antigen (HA) concentration was also measured in the cell lysis of infected devices via ELISA to quantify intracellular viral antigens. Here we also observed a significantly higher antigen concentration in the LOC compared with the Mϕ-LOC and IC-LOC samples (Fig. 2d).

**Extended Data Fig. 5 |:**
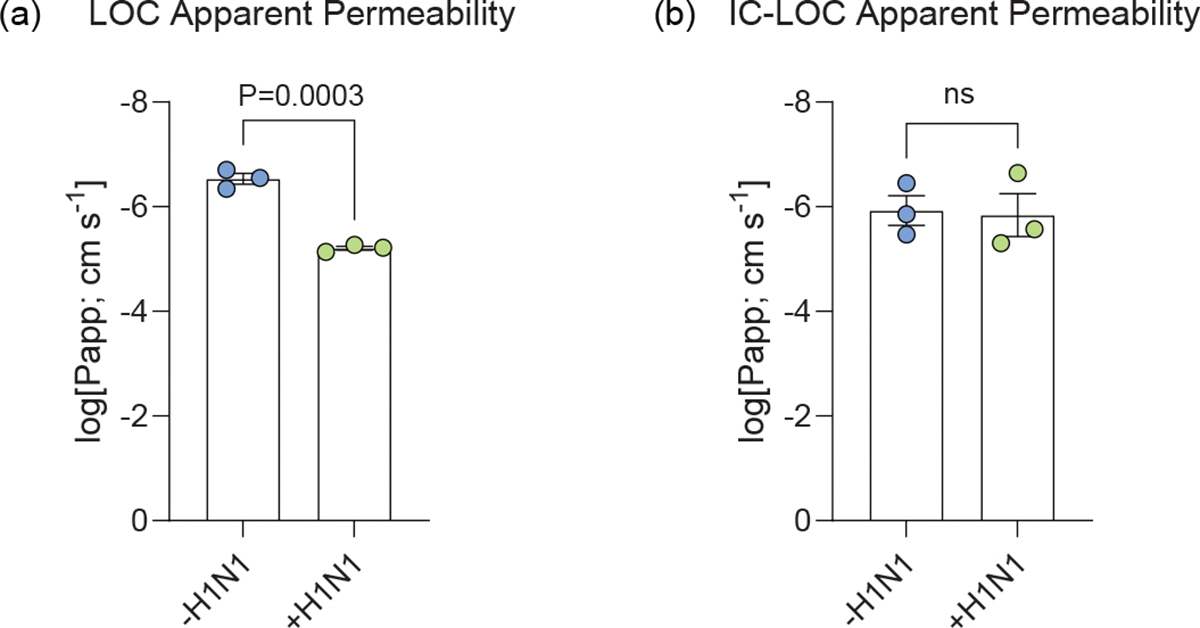
Estimated apparent permeability of the epithelial monolayer in LOC and IC-LOC devices. **(a)** Estimated apparent permeability of the LOC and **(b)** the IC-LOC in uninfected (−H1N1) and severely infected (+H1N1) devices. Significance determined via unpaired two-tailed t test. N = 3 independent devices from the same immune cell donor for the IC-LOC samples.

In addition, we evaluated epithelial damage in the LOC and IC-LOC at varying MOIs via lactate dehydrogenase (LDH) activity and epithelial permeability assays. We observed increased LDH activity with increasing MOI in the LOC devices and a significant increase in epithelial permeability in the LOC at MOI 10, indicative of structural damage to the epithelium (Fig. 2e). This damage was largely prevented in the IC-LOC devices (Fig. 2f). To further quantify the permeability, we took an alternative approach to previous reported methods of apparent permeability calculations. Apparent permeability coefficient (*P*_app_) values are highly context dependent and, in LOC devices, can differ based on the design and material properties of the devices, as well as the experimental conditions^22^. Furthermore, *P*_app_ values are typically calculated using concentration measurements from the basolateral side of the membrane. While this is feasible in more canonical bilayer LOC models, the hydrogel underlying our airway epithelium makes taking direct measurements from the basolateral compartment technically challenging. While samples can be taken from the adjacent media channels, this involves diffusion through the hydrogel along the *y*-axis in addition to the diffusion from the airway chamber along the *z*-axis. Because of this, these measurements do not accurately reflect the airway permeability, and as such, permeability was purposefully presented as the dextran concentration in the airway compartment, as reported elsewhere^20^. However, for ease of comparison to other existing models, we have estimated apparent permeability values by assuming conservation of mass to estimate the amount of dextran that passed across the membrane into the basolateral compartment (Extended Data Fig. 5). Our results show the log[*P*_app_] for uninfected control devices to be around −6 in both the LOC (−6.53) and IC-LOC (−5.93) (Extended Data Fig. 5). In a bilayer LOC model, Si et al.^18^ also reported the apparent permeability (log[*P*_app_]) in a human airway chip with no infection to be approximately −6. In our LOC, 72 h of severe infection with H1N1 virus significantly reduced the log[*P*_app_] from −6.53 to −5.20. In the IC-LOC, log[*P*_app_] was not significantly affected, only decreasing from −5.93 to −5.84, indicating that epithelial barrier permeability was minimally compromised in the IC-LOC. Taken together, the viral quantification and epithelial damage assays confirm the viral titres and infection and elucidate the protective role of the immune cells in the Mϕ-LOC and IC-LOC devices.

We further observed that MOI 10 was necessary to induce high levels of cytokine secretion associated with severe influenza, often referred to as a cytokine storm, with significant spikes in IL-1β, IL-8, TNF-α, CXCL10, IFN-λ1, IFN-λ2/3, IFN-α2, GM-CSF and IL-10 at 72 hpi ranging from 5- to 400-fold increase from time zero (pre-infection) (Fig. 2g). Previous reports indicate that CXCL10 levels in the BAL and plasma uniquely correlate in patients with severe influenza and SARS-CoV-2, with plasma levels of CXCL10 even predicting patient outcomes in a study performed by Reynolds et al.^53^. Here we aimed to illustrate that our system mirrors in vivo observations by detecting CXCL10 as a key cytokine whose elevated levels on the vascular side of the device correlate with elevations on the airway side. We observed that the CXCL10 levels in the airway and vascular regions of the infected IC-LOC device uniquely correlated, while 11 of the additional evaluated cytokines did not (Fig. 2h and Supplementary Fig. 6). Notably, these correlations were absent in the LOC or Mϕ-LOC models (Supplementary Figs. 7 and 8). Furthermore, we discovered that in the IC-LOC, the airway and vascular levels of IFN-λ1, which were not evaluated in the patient data reported by Reynolds et al., also correlated (Fig. 2i). Obtaining cytokine measurements from the BAL fluid in patients is extremely invasive, whereas obtaining plasma samples is comparatively simple; thus, developing a comprehensive array of plasma cytokines to understand airway inflammation could be highly impactful. Here we demonstrate that our IC-LOC, but not LOC or Mϕ-LOC, can recapitulate BAL and plasma cytokine relationships observed in patients.

To further evaluate the ability of the IC-LOC to recapitulate the cytokine storm observed in vivo, we compared the fold changes in secreted cytokines CXCL10, TNF-α and IL-1β in the airway of the IC-LOC to those in the BAL fluid of patients with moderate and severe influenza infection^53^, compared with uninfected IC-LOC devices and healthy patients, respectively. In IC-LOC devices, MOI 10 resulted in an increase in CXCL10 and TNF-α that corresponded to the increase observed in patients with severe influenza (Fig. 2j). Meanwhile, LOC devices were unable to produce physiologically relevant increases in cytokine levels when compared with patient data, regardless of MOI (Fig. 2k). Mϕ-LOC devices only produced physiologically relevant increases in CXCL10, with no significant increase in TNF-α (Supplementary Fig. 9). Meanwhile, for IL-1β, MOI 10 elicited a significant increase only in the IC-LOC, not in LOC or Mϕ-LOC devices (Supplementary Fig. 9). These findings indicate that the IC-LOC system recapitulates the hyperinflammatory environment observed in patients with severe influenza when infected at MOI 10. Therefore, MOI 10 was used in all subsequent experiments to mimic severe influenza infection.

### IC-LOC reveals critical structural changes in infected airways

**Fig. 3 |:**
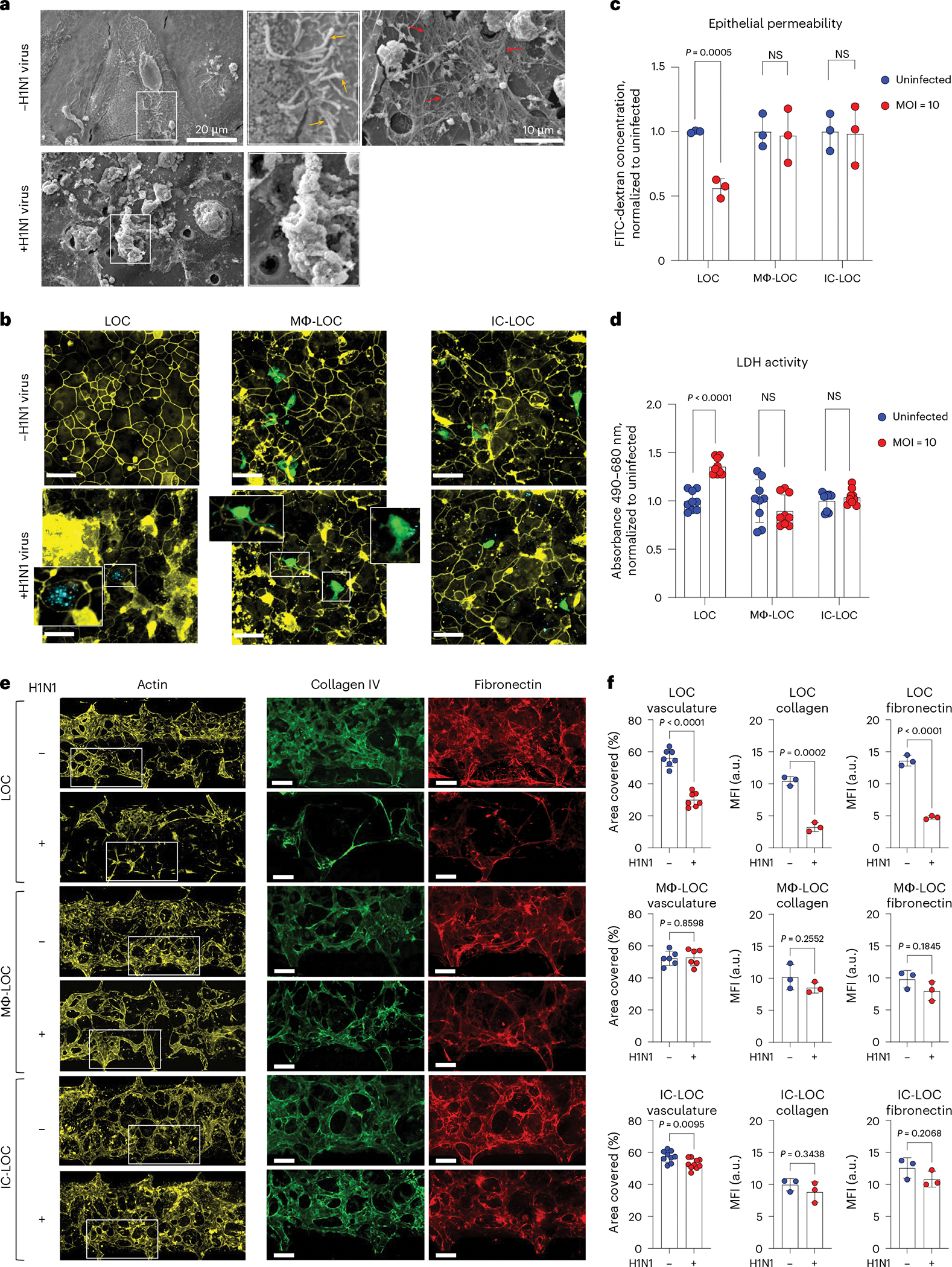
Virus-induced epithelial and endothelial damage in the LOC, Mϕ-LOC and IC-LOC. **a**, SEM of the epithelium in the LOC with and without severe influenza infection. Yellow arrows, cilia. Red arrows, mucus. **b**, Epithelial H1N1-mediated damage in the LOC, Mϕ-LOC and IC-LOC shown by irregularity in ZO-1 (yellow) staining, with H1N1 shown in cyan and AMs labelled with CellTrace Green (green). **c**, Quantification of epithelial barrier function via epithelial permeability assay with 10 kDa FITC-dextran. The graph shows FITC-dextran concentration in the airway channel after 2 h incubation, normalized to uninfected controls. *N* = 3 independent devices. **d**, Quantification of epithelial damage via LDH activity as determined via a colorimetric assay. Absorbance values shown normalized to uninfected controls. *N* = 8 independent devices. **e**, Representative images of the ECM in LOC, Mϕ-LOC and IC-LOC in severely infected and uninfected devices. Images show the vascular network (actin, yellow) and related ECM proteins (collagen, green; fibronectin, red). **f**, Quantification of the percent area covered by the vasculature in the LOC, Mϕ-LOC and IC-LOC. In addition, quantification of the collagen and fibronectin levels, as quantified by MFI, in each device in response to infection are also shown. *N* = 7 independent devices for vasculature analysis and *N* = 3 independent devices for collagen and fibronectin analysis. Scale bar for all images, 160 μm. All immune-competent devices use immune cell from the same immune cell donor. Statistics represent two-tailed unpaired *t*-tests, with *P* value shown on each graph. Data are presented as mean values ± s.d.

In patients, severe H1N1 infection results in a disruption of lung integrity. However, whether specific immune cell subtypes contribute to the exacerbation or prevention of damage to structural cells within the lung microenvironment remains unclear. To this end, we first evaluated structural changes in the LOC epithelium in response to severe influenza infection through scanning electron microscopy (SEM). Healthy, uninfected devices showed an intact monolayer with cilia and mucus secretions (Fig. 3a). Meanwhile, upon severe influenza infection, SEM imaging revealed substantial epithelial disruption (Fig. 3a). To further investigate the specific role of both tissue-resident and circulating immune cells on H1N1-mediated epithelial damage, we evaluated the presence of tight junction protein ZO-1 in the epithelial layer of various LOC devices 72 hpi using confocal microscopy (Fig. 3b). The LOC devices, devoid of immune cells, experienced significant epithelial ZO-1 disruption upon H1N1 infection. By contrast, the presence of immune cells in the Mϕ-LOC and IC-LOC models reduced the tight junction irregularity. High-resolution confocal microscopy further revealed active uptake of viral particles by AMs (Fig. 3b, insets). We also observed that the viral load within epithelial cells was lessened by the presence of AMs (Fig. 3b, insets). Epithelial permeability and LDH activity analysis across LOC, Mϕ-LOC and IC-LOC samples revealed that both the Mϕ-LOC and IC-LOC largely prevented the epithelial damage observed in LOC devices (Fig. 3c,d). These findings demonstrate the key role that tissue-resident and circulatory immune cells play in maintaining the integrity of the epithelial monolayer in these devices.

In addition to characterizing the effect of immune cell incorporation and H1N1 infection on the epithelium, we also investigated their effect on the vascular network. Using AngioTool (version 0.5), we quantified the vascular percent area coverage and average vessel length. We observed that in the LOC, H1N1 infection destroyed the vascular network, as can be qualitatively observed in the representative images shown in Fig. 3e. Quantitative analysis revealed that H1N1 infection in the LOC significantly decreased vasculature percent area coverage and average vessel length (Fig. 3f and Supplementary Fig. 10). However, tissue-resident macrophages in the Mϕ-LOC mitigated the observed vascular damage. With the presence of macrophages, H1N1 infection had no significant effect on vascular percent area covered or average vessel length. While the overall vascular network of the IC-LOC was largely maintained in the severe H1N1 condition, the infected IC-LOC showed few areas of vascular disruption as evident with an overall modest but significant decrease in the percent area covered compared with the uninfected devices (Fig. 3f). This is unsurprising, as severe influenza infection is known to cause vascular disruption through the high levels of secreted cytokines^54^. Nevertheless, as with the airway layer of the device, incorporating tissue-resident immune cells in the Mϕ-LOC and IC-LOC can protect the vascular network from undergoing significant structural damage upon severe infection. ECM remodelling also occurs in response to lung respiratory infections^55^. These changes include increased deposition of ECM fragments and collagen, and massive formation and deposition of fibronectin, among others. However, these observations are largely made from patients with SARS-CoV-2^55^. In mice, SARS-CoV-2 infection increased collagen deposition in the lungs compared with influenza-infected mice. It remains unclear how the immune competency of lung tissues impacts alterations to the ECM in response to severe H1N1 infection. Thus, to investigate the impact of immune cells on the extracellular environment of the lung in severe H1N1, we quantified the effect of H1N1 infection on secreted ECM proteins. In the immune-deficient LOC, severe H1N1 infection significantly reduced collagen IV and fibronectin deposition in the interstitium (Fig. 3e,f). Specifically, the percent area covered by collagen IV was significantly lower in LOC devices with severe H1N1 infection, indicating that fewer cells were secreting this ECM protein, even after normalization to the actin percent area covered to account for differences in total cells present. Similar to collagen IV, fibronectin secretion levels were also lower in H1N1-infected LOC devices, indicating a change in the interstitial ECM composition. The mean fluorescence intensity (MFI) was significantly lower in response to H1N1 infection, indicating that the cells were producing lower levels of these ECM proteins (Fig. 3f). By contrast, in the Mϕ-LOC and IC-LOC devices, collagen IV and fibronectin levels were modestly but insignificantly reduced, therefore suggesting that the local niche was largely maintained. With this, we find that the lack of immune components may result in an overstated impact of viral infection in vitro, as immune competency appears to significantly protect from viral-induced structural damage.

### IC-LOC demonstrates immune cell influx and viral uptake

**Fig. 4 |:**
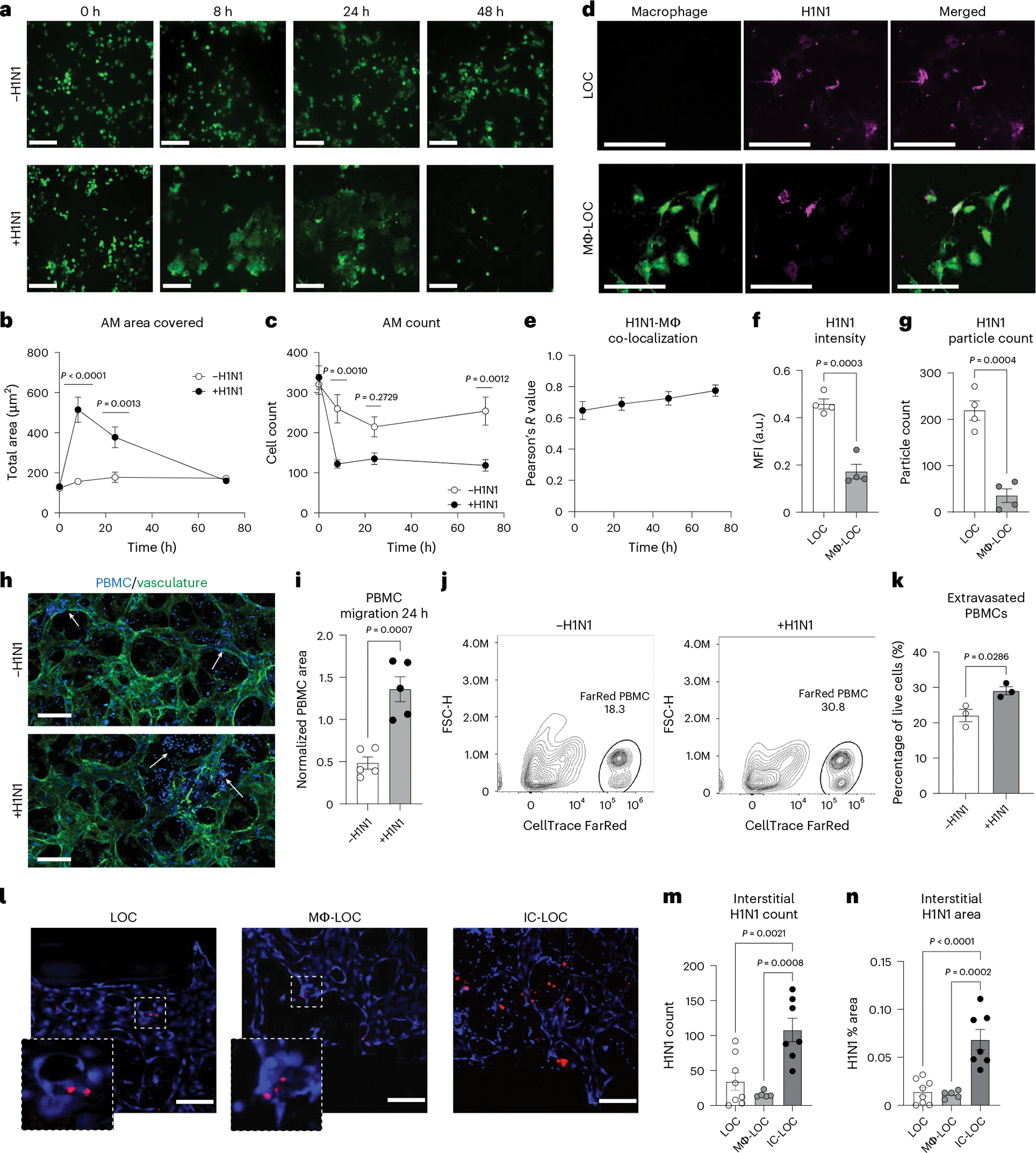
Immune-mediated viral sequestration and trafficking in severe influenza infection on chip. **a**, Representative images of the change in AM size, shape and abundance over time in the Mϕ-LOC device with and without severe influenza infection (MOI = 10). Scale bars, 160 μm. **b**, Quantification of the number of AMs observed in devices with and without severe infection. *N* = 10 independent devices. **c**, Quantification of the total area covered by AMs in devices with and without severe infection. *N* = 10 independent devices. **d**, AM (green) co-localization with PKH26 dye-labelled H1N1 viral particles (magenta). Scale bars, 160 μm. **e**, Co-localization of AMs with labelled H1N1 particles. *N* = 7 independent devices. **f**,**g**, MFI (**f**) and particle count (**g**) of the labelled H1N1 signal in LOC devices with and without tissue-resident macrophages. *N* = 4 independent devices. **h**, PBMC infiltration into the interstitial layer of the IC-LOC device, with and without severe influenza infection (vasculature, green; pre-labelled PBMCs, blue). Scale bars, 160 μm. **i**, Quantification of labelled PBMC infiltration into the interstitial layer at 24 hpi. *N* = 5 independent devices. **j**, Representative flow cytometry analysis plots of the CellTrace FarRed labelled PBMC population from the central channel of the IC-LOC. **k**, Percentage of live cells from the central channel that are PBMCs (CellTrace FarRed^+^) in uninfected and infected IC-LOC devices. *N* = 3 independent samples, with each sample = 4 pooled devices. **l**, Representative images showing viral nucleoprotein (H1N1 NP, red) staining in the vascular layer (DAPI, blue). Scale bars, 150 μm. **m**,**n**, Quantification of interstitial H1N1 particle count (**m**) and area (**n**), as determined by H1N1 nucleoprotein staining in LOC, Mϕ-LOC and IC-LOC devices, determined via ordinary one-way ANOVA followed by Tukey’s multiple comparisons test. *N* = 7 independent devices. All immune-competent devices use immune cells from the same immune cell donor. All statistical significance determined via two-tailed unpaired *t*-test unless otherwise noted. Data are presented as mean values ± s.d.

To further understand the role of AMs in the progression of H1N1 infection, we labelled macrophages with CellTracker Green and imaged the airway epithelium periodically over the 72 h infection period (Fig. 4a). We found that at 8 hpi, the AMs in the infected devices increased dramatically in size in response to infection (Fig. 4b), a phenomenon that has not been previously reported. The overall number of AMs also plummeted after 8 h in the infected devices but remained relatively stable in uninfected devices (Fig. 4c). Furthermore, it was observed that the AMs co-localized with PKH26 fluorescent cell linker dye-labelled H1N1 viral particles as shown by a high Pearson’s *R* value, determined using ImageJ (version 1.54) co-localization analysis, ranging from 0.64 to 0.79 and increasing over time (Fig. 4d,e). At the 72 h endpoint, both the MFI of the viral signal and the absolute number of observable viral particles in the epithelium were higher in devices without macrophages (Fig. 4f,g). This, along with the co-localization, suggests that the AMs uptake the virus upon initial infection and protect the lung from the progression of infection.

**Extended Data Fig. 6 |:**
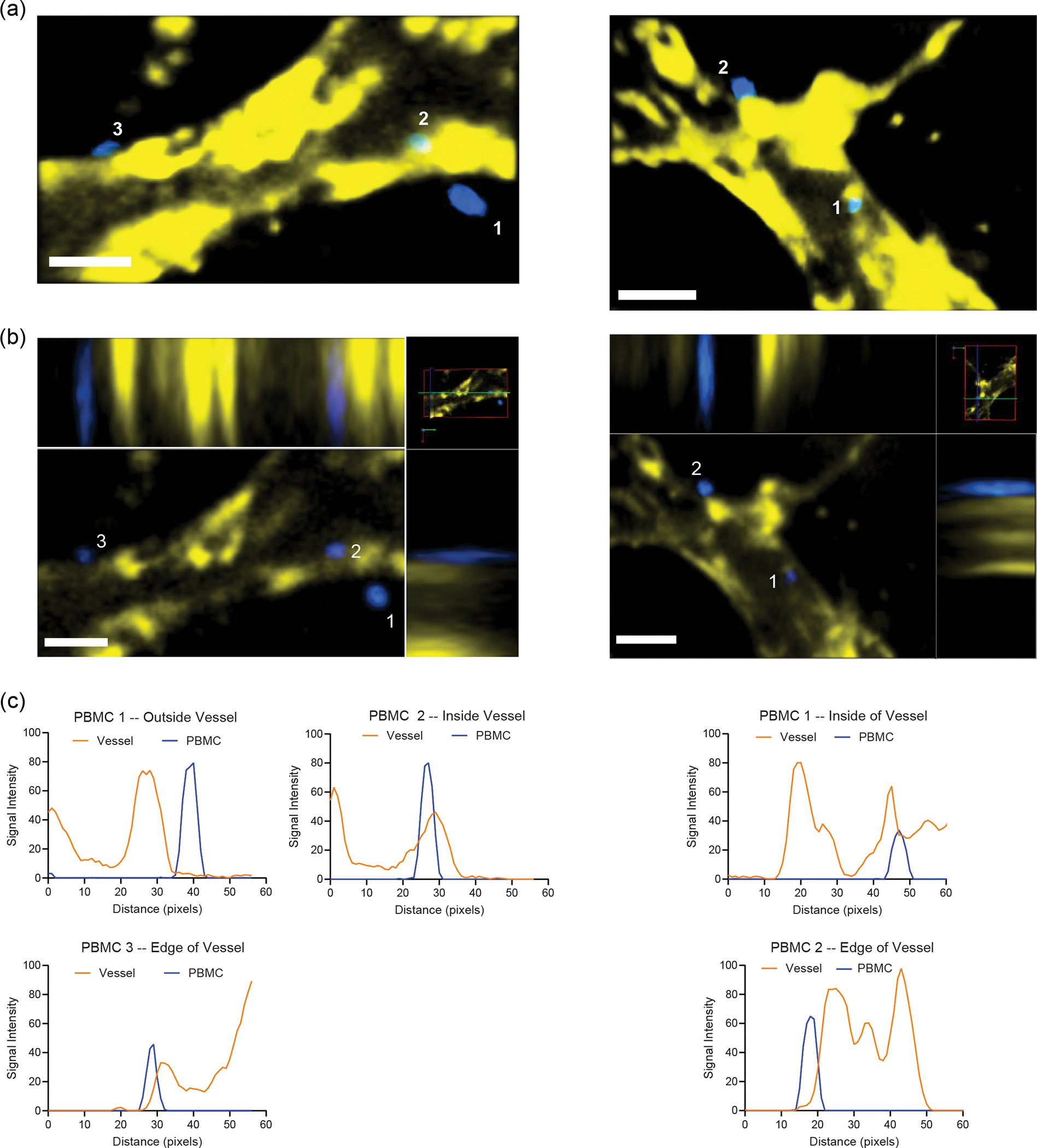
Circulatory immune cell extravasation in the IC-LOC in response to severe H1N1 infection. **(a)** Representative maximum intensity projection and **(b)** orthogonal projection images of PBMCs (blue) and endothelial vessels (yellow) with **(c)** plot profiles showing PBMCs inside vessels, outside vessels, and at the vessel walls.

The recruitment of circulating immune cells from the vascular space into infected tissue is central to the lung pathology in influenza infection^25,56^. However, the most relevant previous work using a human airway chip and influenza infection in an in vitro model has used only primary neutrophils. A major caveat of previous systems is that they lack the interstitial space and chemokine gradients generated across these various epithelial and endothelial barriers, including basement membranes, during viral infections. However, in influenza infection in patients, the collagen-rich interstitium plays a vital role where immune cells such as neutrophils and T cells navigate through the interstitium, guided by chemokines to be recruited to the airway space^57^. To overcome this technical barrier and knowledge gap, we characterized the effect of H1N1 infection on PBMC migration in the IC-LOC by labelling PBMCs with CellTrace Violet and perfusing them through the microvascular network (Fig. 4h,i). We observed a significant increase in migration of PBMCs from the media channels into the central channel of the device in response to H1N1 infection, as measured by the percent area covered by the labelled PBMCs at 24 hpi, normalized to the area covered by PBMCs at time zero to account strictly for migration into the central channel in response to infection (Fig. 4i). Importantly, we also observed a qualitative increase in PBMCs within the central channel that had extravasated from the vessels into the interstitial space in the infected devices. By contrast, in uninfected devices, the PBMCs within the central channel primarily remained within the microvascular network (Fig. 4h, arrows, and Extended Data Fig. 6). This qualitative observation was further validated through flow cytometric analysis of the central channel of IC-LOC devices with perfused PBMCs labelled with CellTracker FarRed. IC-LOC devices were flushed with PBS to remove any free PBMCs, and then the central channel hydrogel was degraded, collected and evaluated via flow cytometry. We observed a significant increase in the percentage of extravasated PBMCs present in the central channel of infected devices (Fig. 4j,k). Thus, we not only observed an increase in PBMC infiltration from the media channels into the vascular network, but also we observed an increase in PBMCs which extravasated into the interstitial space in the presence of an H1N1-infected epithelium, reminiscent of the immune cell infiltration and recruitment observed in severe H1N1 in vivo. To the best of our knowledge, our work is the first demonstration of the recruitment of PBMCs through an endothelial network and into interstitial space in an in vitro lung infection model.

Arguably, the recruitment of PBMCs from the vascular network to the interstitium could be due to virus infiltration in the interstitial space. Using confocal microscopy, we probed the interstitium of infected IC-LOC devices for the influenza A nucleoprotein (NP) (Fig. 4l–n). Indeed, we observed a substantial viral signal in the interstitium of the IC-LOC in both observable particle count and percent area covered. However, HUVECs are relatively resistant to direct influenza infection, and, accordingly, minimal viral signal was detected in the interstitial layer of the LOC and Mϕ-LOC devices (Fig. 4m,n). This suggests that interstitial DCs and extravasation of circulating immune populations may be necessary for viral trafficking from the epithelium.

### Severe infection in IC-LOC induces systemic cytokine storm

In vivo, severe H1N1 infection is characterized by significantly elevated levels of inflammatory cytokines, including but not limited to IFNs, IL-1, IL-6 and IL-10 in both the BAL fluid and serum^53,58–60^. In recent years, high systemic levels of cytokines and their association with disease severity have frequently been referred to as a ‘cytokine storm’. However, it remains unclear whether specific inflammatory molecules drive lung pathology or serve as correlates of other tissue-level mechanisms. To study this, we characterized the cytokine response to severe H1N1 infection from samples collected from both the airway and interstitial layers of infected and uninfected LOC, Mϕ-LOC and IC-LOC devices at various timepoints, including 0, 2, 24 and 72 hpi.

**Fig. 5 |:**
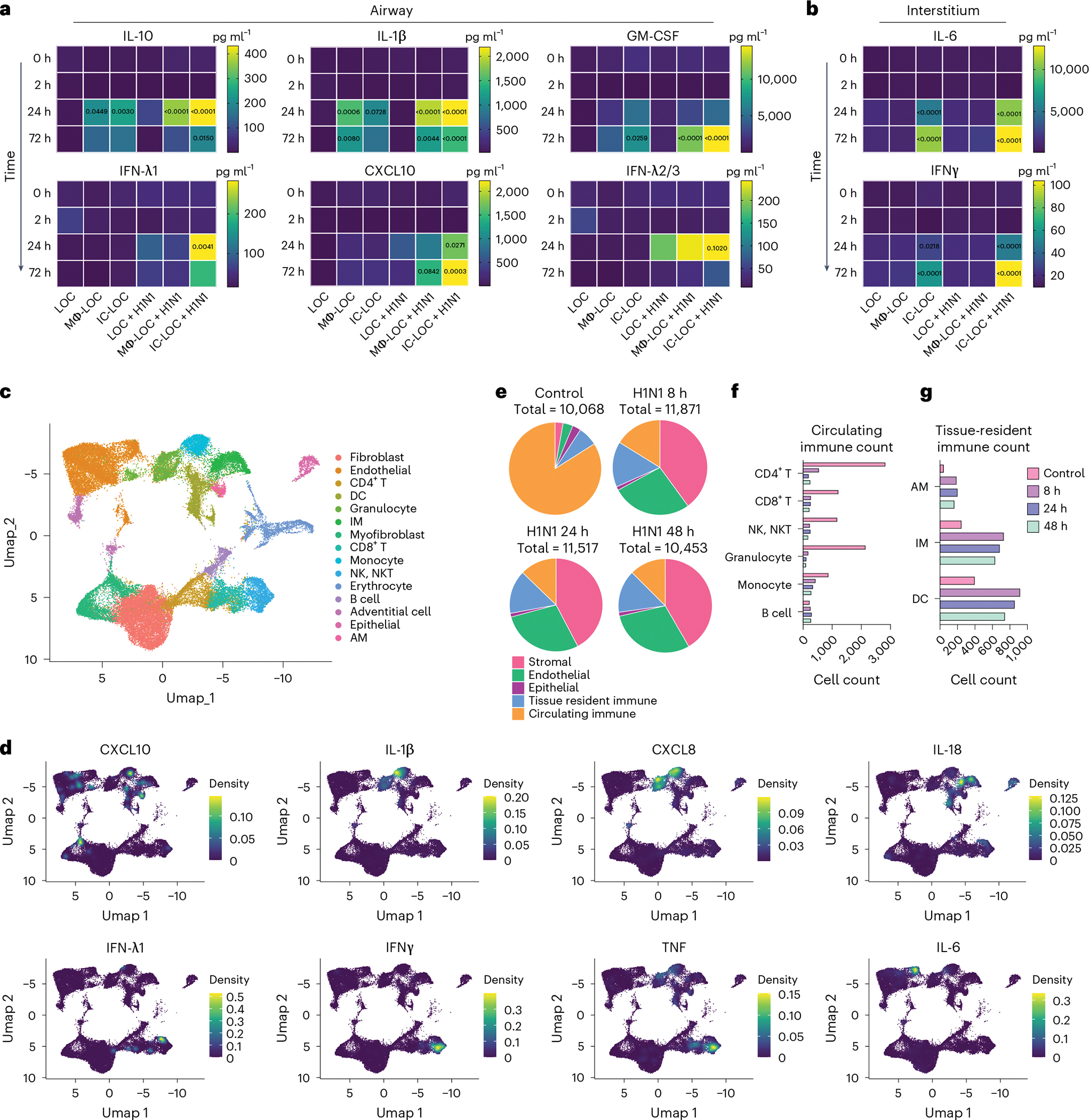
The IC-LOC recapitulates a virally induced cytokine storm. **a**,**b**, Secreted pro-inflammatory cytokine levels in the epithelial airway (**a**) and interstitial vascular (**b**) layers of the device in healthy and infected (H1N1, MOI = 10) conditions, statistical significance compared with time 0 h determined via ordinary one-way ANOVA with *P* values shown on the charts. *N* = 6–7 independent devices from the same immune cell donor across at least 2 independent experiments. **c**, UMAP visualization of the integrated datasets (control, 8 h, 24 h and 48 h) identifying 15 distinct cell types within the IC-LOC. **d**, Visualization of select key cytokine expression patterns in UMAP space using the kernel density estimation method in the R package Nebulosa. **e**, Pie charts depicting the relative proportions of stromal, endothelial, epithelial, tissue-resident immune and circulating immune cells identified in the scRNA-seq data. **f**,**g**, Raw counts of circulating (**f**) and tissue-resident (**g**) immune cells from the scRNA-seq data.

In the LOC, only a modest cytokine response was observed in response to infection (Fig. 5a,b and Supplementary Figs. 11 and 12), with IFN-λ2/3 being the most highly secreted cytokine. This is expected as IFN-λ is secreted primarily by epithelial cells in response to infection^61^. The Mϕ-LOC produced a notable increase in inflammatory cytokines in the airway layer, with elevated levels of IL-10, IL-1β, GM-CSF and CXCL10 in response to infection, but failed to generate a significant interstitial response (Fig. 5a,b and Supplementary Figs. 11 and 12). However, in the IC-LOC, we observed a considerable cytokine response generated in both the airway and the interstitial layers with a significant increase in IL-10, IL-1β, GM-CSF, IFN-λ1, CXCL10, IFN-λ2/3, IL-6 and IFNγ. These findings, when coupled with the lack of detectable viral signal in the endothelium of infected LOC and Mϕ-LOC devices, indicate that full immunocompetency results in a broad spectrum increase in cytokines and other inflammatory proteins, which could potentially have a damaging impact on the lung endothelium structure.

**Extended Data Fig. 7 |:**
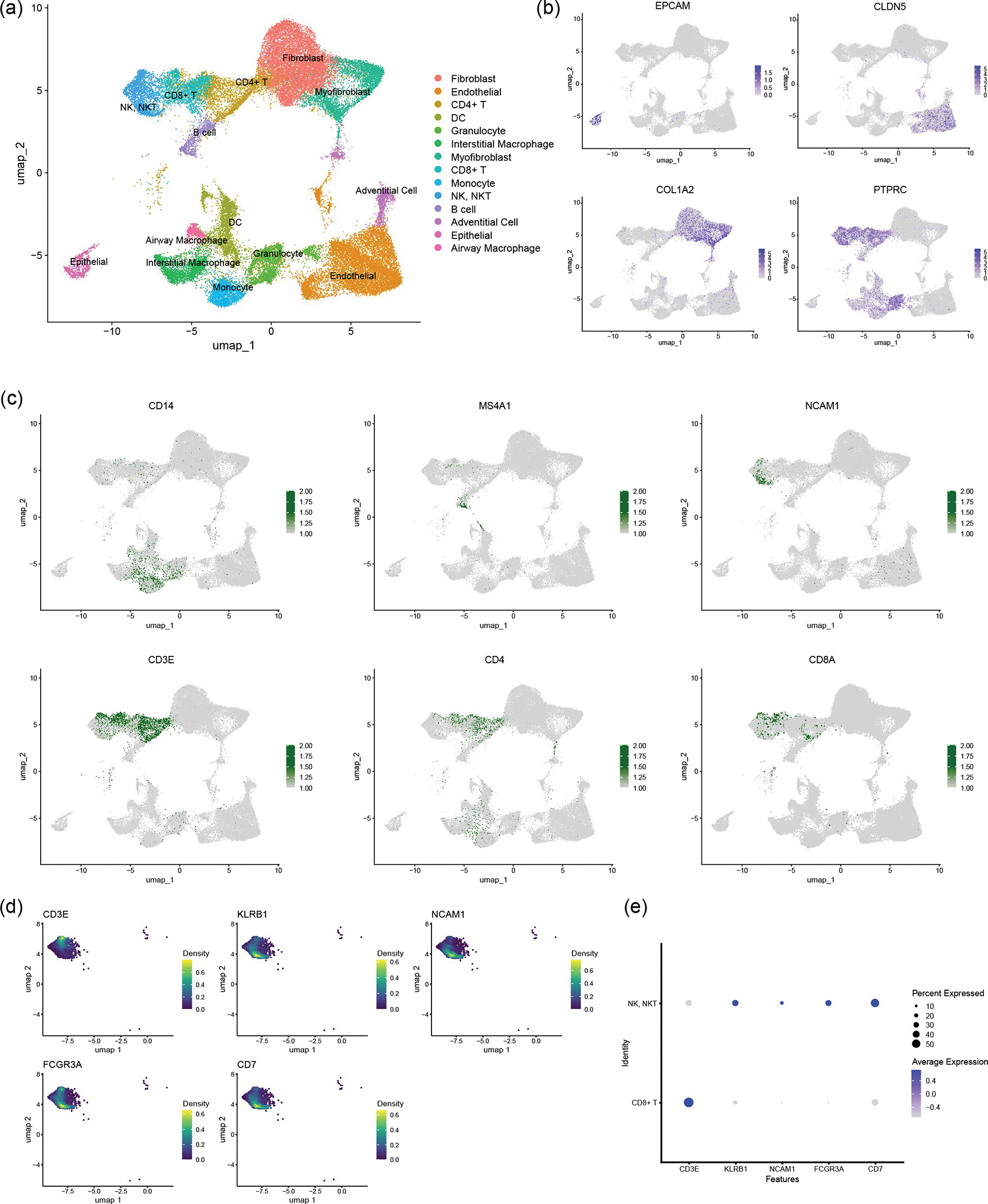
Cell type annotations of the IC-LOC scRNA-sequencing dataset. **(a)** UMAP of integrated dataset containing uninfected control IC-LOC sample and infected IC-LOC samples from 8-, 24-, and 48-hours post-infection, resulting in 15 distinct cell populations. **(b)** The canonical markers used to define the four major categories of cells within the IC-LOC – epithelial (EPCAM), endothelial (CLDN5), stromal (COL1A2), and immune (PTPRC). **(c)** The canonical markers used to identify the major immune cell types within the IC-LOC – myeloid (CD14), B cell (MS4A1), NK/NKT cell (NCAM1), T cell (CD3E), and specifically CD4 + T cell (CD4) and CD8 + T cell (CD8A). **(d)** Kernel density plots of select genes within the “NK, NKT” cluster, leading to its designation. **(e)** Dot plots of select genes showing average expression and percent expressed within the “NK, NKT” cluster and “CD8 + T” cluster.

Through scRNA-seq analysis of both healthy and infected IC-LOC devices, we could also identify several key producers of inflammatory cytokines. Specifically, we compared samples from control uninfected devices and devices that had been infected with H1N1 for 8 h, 24 h and 48 h timepoints and analysed the resulting transcriptomic data using the Seurat package in R^47–50^. To ensure that any observed effects were not due to differences in the length of device culture, all devices were cultured for the same amount of time, and viral infection was initiated at 8 h, 24 h or 48 h before the experiment endpoint, with control devices cultured for the full time with no infection. Here we utilized RBC-depleted whole blood to incorporate both leukocytes and granulocytes in the circulating immune populations, in addition to the tissue-resident IMs, DCs and AMs. Cells were combined from both layers of the IC-LOC devices for sequencing, and gene expression profiles were compared with known datasets and used to annotate cell type and subset^62^. Through scRNA-seq analysis, we were able to detect 15 distinct cell types, including endothelial cells, fibroblasts, myofibroblasts, adventitial cells, epithelial cells, IMs, AMs, DCs, monocytes, granulocytes, CD4 T cells, CD8 T cells, NK/NKT cells, B cells and erythrocytes, the last of which were excluded during analysis (Fig. 5c and Extended Data Fig. 7).

We then probed for inflammatory cytokines to identify key players in the observed cytokine storm. To this end, we found that monocytes were largely responsible for the production of IL-10 and IL-1β, IMs were the main producers of IL-18, granulocytes produced significant levels of IL-8, and NK/NKT and T cells were largely responsible for IFNγ, IFN-λ1 and TNF production (Fig. 5c,d). Notably, we found that endothelial and stromal cells produced significant amounts of IL-10, IL-12 and IL-6, aligning with the notion that lung endothelial cells are key orchestrators of cytokine amplification during influenza virus infections^57^. In the multiplexed cytokine analyses (Fig. 5a,b), we did not observe significant levels of these cytokines secreted in either the infected LOC or Mϕ-LOC. Thus, we hypothesize that the activation of circulating immune cells is necessary to exacerbate the activation of endothelial and stromal cells and induce the secretion of this inflammatory milieu, further underscoring the necessity of the fully immunocompetent IC-LOC to mimic the antiviral inflammatory immune response and highlighting the complex signalling cascade that occurs in severe influenza.

### Severe H1N1 infection alters stromal and immune populations

Analysis of scRNA-seq data revealed an overall temporal change in cellular proportions in response to severe influenza infection, which remains poorly understood owing to a lack of controlled human influenza studies from patients with severe influenza. Specifically, the circulating immune populations (defined as all T cells, B cells, NK/NKT cells, monocytes and granulocytes) accounted for 83.9% of the cells in the uninfected control sample, compared with only 16.2%, 12.8% and 12.5% in the 8 h, 24 h and 48 h of severely infected samples, respectively (Fig. 5e). We specifically observed a substantial decrease in CD4 and CD8 T cells, NK/NKT cells and granulocytes, with a modest decrease in monocytes. Notably, the B cell population numbers remained similar or even increased in response to H1N1 infection (Fig. 5f). Meanwhile, the tissue-resident immune populations (defined as AMs, IMs and DCs) increased slightly (Fig. 5g).

**Extended Data Fig. 8 |:**
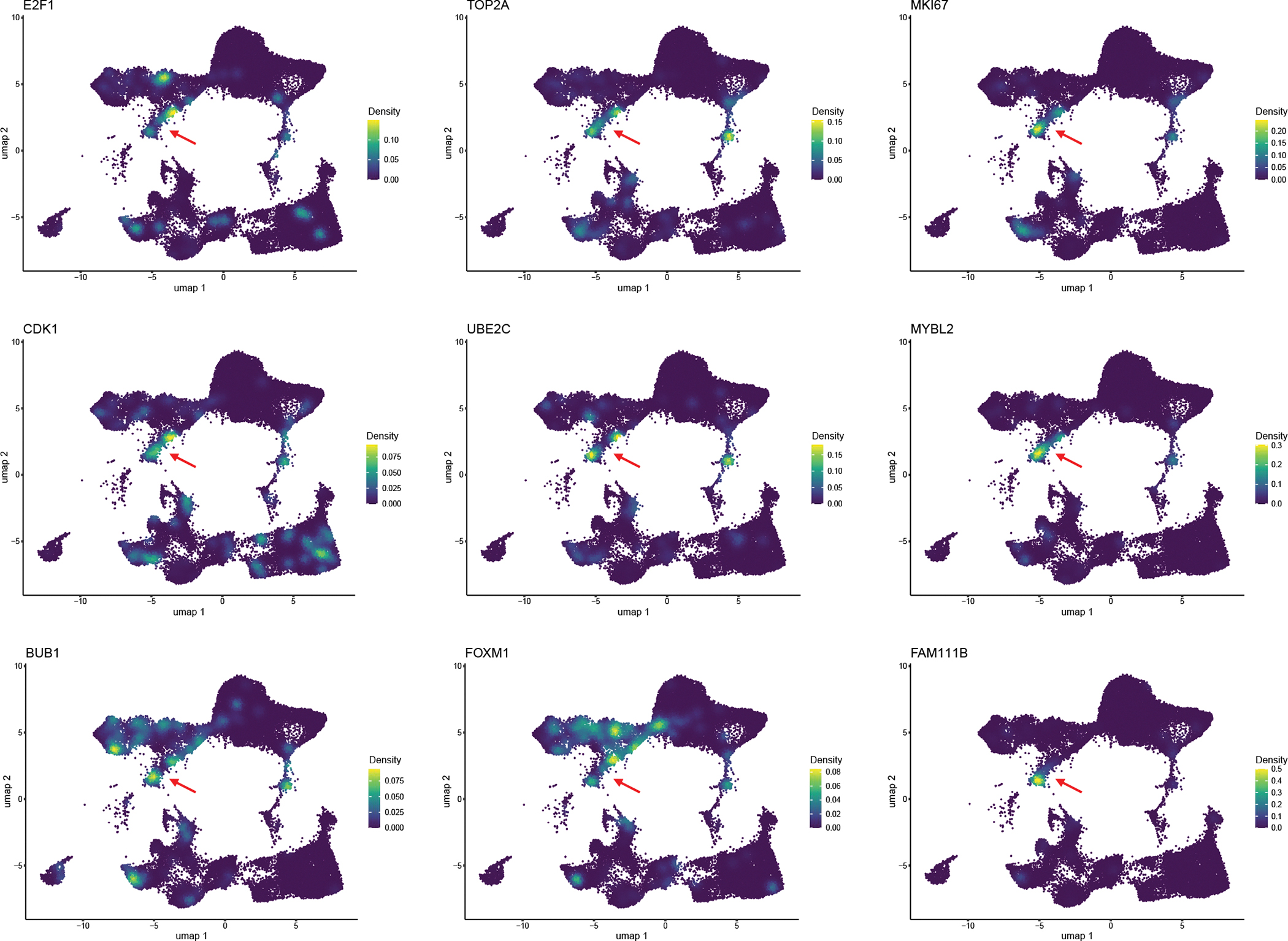
B cell proliferation in the IC-LOC. Visualization of proliferation marker *E2F1, TOP2A, MKI67, CDK1, UBE2, MYBL2, BUB1, FOXM1*, and *FAM111B* expression patterns in UMAP space using the kernel density estimation methods in the R package Nebulosa. The red arrow indicates the location of the B cell cluster.

We observed a notable decrease in epithelial cells, most likely in direct response to viral infection. By contrast, the percentage of stromal and endothelial cells increased significantly—likely as an artefact of cell survival as opposed to cell proliferation^54,63^. To confirm this, we evaluated the expression patterns of various proliferation markers, including *PIF1*, *CDK1*, *UBE2C*, *FAM111B* and *TOP2A*, and found that the stromal and endothelial cells did not express substantial levels of any of the evaluated proliferation markers. However, this did yield an interesting finding in which B cells within the infected devices were the most prominently proliferating cell type (Extended Data Fig. 8), in line with our previous observation of the B cell proportions in infected devices.

### Severe infection boosts stromal–immune crosstalk in IC-LOC

**Fig. 6 |:**
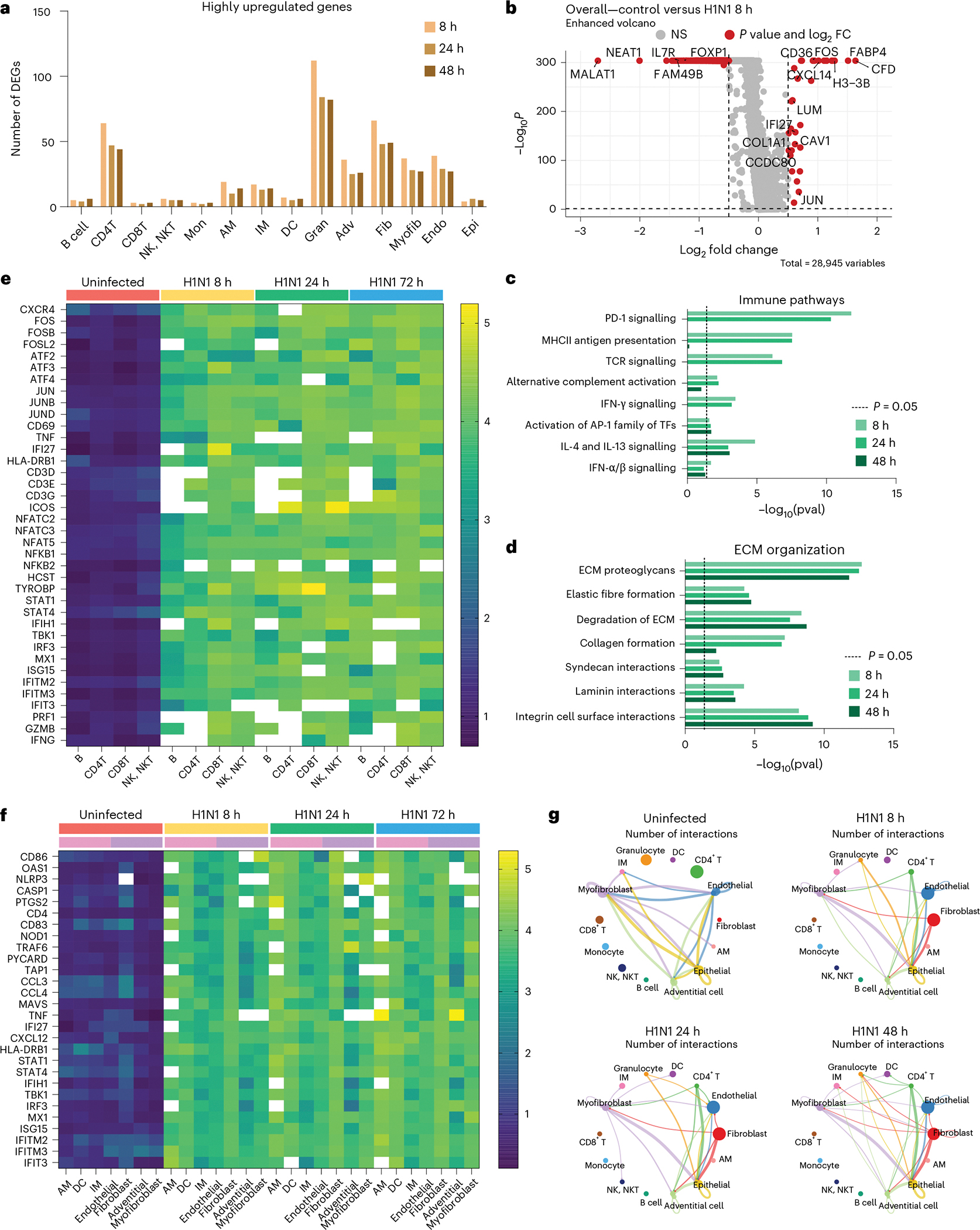
Severe influenza infection induces transcriptional shutdown and activation of ECM reorganization and antiviral immune pathways. **a**, The number of highly upregulated genes (FC >1.5) per cell type compared with the control devices. **b**, Volcano plot visualization of DEG analysis, highlighting all genes significantly highly (*P* < 0.05, |FC| >1.5) up- or downregulated across all cell populations when comparing uninfected control to 8 h H1N1 infection IC-LOC devices. Statistical significance determined via Wilcoxon rank sum test. **c**,**d**, Pathways represented in the top 10% of upregulated genes in each infected condition, comprised primarily of immune pathways (**c**) and ECM interactions (**d**). Statistical significance determined via binomial test. **e**, Heat map of expression levels of inflammatory, antiviral and immune activation genes in B, CD4 T, CD8 T and NK/NKT cells from uninfected and infected devices showing increased expression levels in the infected devices. Heat map shows average expression levels of each gene for cells with non-zero expression. White boxes represent genes that are not expressed in the given cell type. **f**, Heat map of expression levels of innate immune response genes in AM, DC, IM, endothelial, fibroblast, adventitial and myofibroblast populations. **g**, Top 10% of CellChat predicted cell–cell interactions, demonstrating an increase in both stromal and immune cell interactions in response to infection. DEGs, differentially expressed genes; Mon, monocytes; Gran, granulocytes; Adv, adventitial cells; Fib, fibroblasts; Myofib, myofibroblasts; Endo, endothelial cells; Epi, epithelial cells; TFs, transcription factors; pval, *P* value.

In addition to a significant variation in cellular proportions, we observed a major transcriptional shutdown in response to severe influenza infection in the IC-LOC. The median number of genes per cell in the control sample was 2,792 compared with 218, 171 and 155 in the 8 h, 24 h and 48 h infection samples, respectively. Although we observed a major transcriptional shutdown in response to severe H1N1 infection, as evidenced by the downregulation of many ribosomal genes, a small number of genes were also upregulated in response to infection (Fig. 6a and Supplementary Fig. 13). Across the 8 h, 24 h and 48 h infection samples, 215, 157 and 160 unique genes, respectively, were highly upregulated (fold change (FC) >1.5) compared with the control.

When evaluating total differential gene expression levels between infected and control devices, we found that most of the largely downregulated genes (FC <−1.5) corresponded to cell cycle and transcriptional regulatory genes such as *MALAT1*, *NEAT1* and *FAM49B* (Fig. 6b and Supplementary Figs. 14–16). *IL7R* was also significantly downregulated, as is typical following TCR engagement^64^. When considering genes that were upregulated in the total cell population, we identified many genes canonically involved in immune cell recruitment and response, such as *CXCL14*, *CFD* and *IFI27*. *CD36*, a scavenger receptor that responds to pathogen-associated molecular patterns and damage-associated molecular patterns, was also significantly elevated. Beyond these, ECM-related genes such as *COL1A1* and *CCDC80*, positive regulators of ECM reorganization and cell-substrate adhesion, were also elevated.

Pathway analysis revealed that the H1N1-upregulated genes corresponded to immune response pathways, including MHC-II antigen presentation, TCR signalling and IFN signalling, as well as ECM remodelling pathways, including ECM proteoglycans, ECM degradation, laminin interactions and integrin cell surface interactions (Fig. 6c,d), as determined using Reactome pathway analysis tools^65–67^. While we did not observe significant differences in the fluorescence microscopy of secreted collagen IV and fibronectin in response to influenza infection, the transcriptomic analysis revealed that severe influenza had a substantial effect on the ECM milieu and cell–ECM interactions in the IC-LOC, beyond simple collagen and fibronectin levels (Fig. 6d). Using gene set enrichment analysis (GSEA), we identified key pathways upregulated in each cell type subset compared with the control^68^. Notably, some of the most upregulated pathways included the inflammatory response, IFN-α response, IFN-γ response and oxidative phosphorylation (Supplementary Fig. 17). The key cell types involved in these upregulated pathways included endothelial cells, fibroblasts, adventitial cells, epithelial cells, DCs and granulocytes. Stromal cells are heavily represented in these pathways and thus highlight the importance of including the interstitium in in vitro infection models of the lung.

While the differentially expressed genes and upregulated pathways alluded to overall changes, we next evaluated gene expression patterns across specific cell types in the IC-LOC in response to severe infection. Here we evaluated levels of key activation and antiviral response genes in the uninfected and infected (8 h, 24 h and 48 h) conditions. We first considered the adaptive immune populations within the IC-LOC, including B, CD4 T, CD8 T and NK/NKT cells (Fig. 6e). Cells were first filtered to only account for those that had non-zero expression of the target genes, and then average expression levels were calculated for each gene of interest. We probed for markers of adaptive immune cell (T/B/NK/NKT) activation (*CD69*, *TNF*, *HLA-DRB1*, *CD3*, *ICOS*, *GZMB*, *PRF1* and *IFNG*), the AP-1 transcription factor complex (*FOS*, *FOSB*, *FOSL2*, *ATF2–4*, *JUN*, *JUNB* and *JUND*), NF-κB pathway (*NFKB1/2*), and IFN response and antiviral genes (*STAT1/4*, *IFI27*, *IFIH1*, *IRF3*, *ISG15*, *IFITM2/3* and *IFIT3*). Across all evaluated genes, we saw significant increases in expression in adaptive immune cells from infected devices from as early as 8 hpi and increasing over time. We then probed the tissue-resident immune populations (AM, DC and IM) for similar activation and antiviral response genes, specifically innate immune cell activation markers (*CD86*, *CD83* and *HLA-DRB1*), IFN-stimulated genes (*OAS1*, *MX1*, *ISG15*, *IFITM2/3*, *IFT3*, *IFI27* and *STAT1/4*), inflammasome components (*NLRP3*, *CASP1* and *PYCARD*), and cytokine and secreted signalling genes (*TNF*, *CXCL12*, *CCL3* and *CCL4*) (Fig. 6f). We observed significant increases in these innate immune responses in the stromal components (fibroblast, adventitial cell and myofibroblast) of the IC-LOC as well as in the expected innate immune cell types (Fig. 6f), highlighting the role of both the stromal and tissue-resident immune cells in the tissue-level response to infection.

**Extended Data Fig. 9 |:**
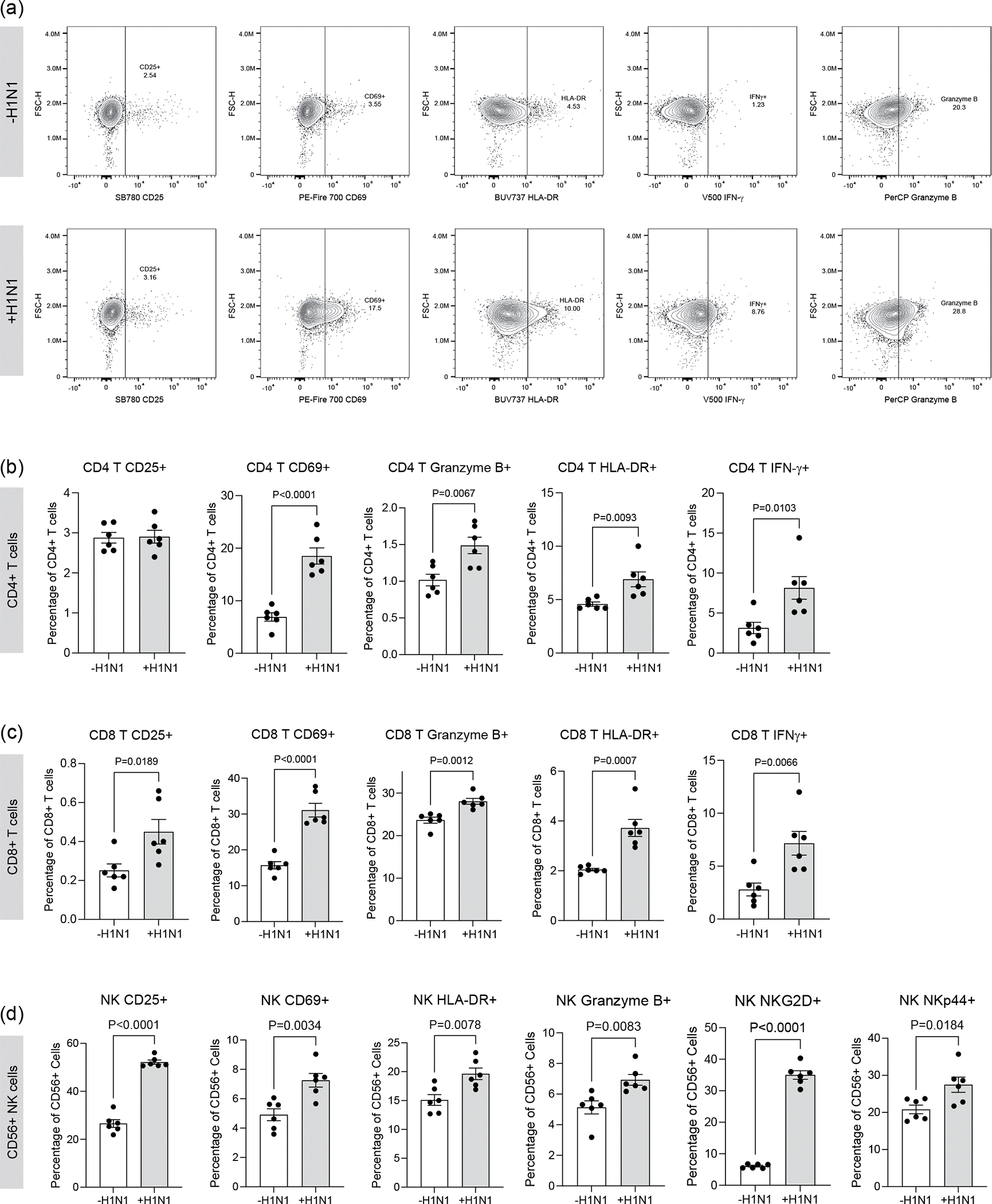
T and NK cell activation at 3 days post-infection in the IC-LOC. **(a)** Representative flow cytometry plots showing levels of activation markers (CD25, CD69, HLA-DR, IFN-γ, Granzyme B) in CD8 T cells from uninfected (−H1N1) and infected (+H1N1) IC-LOC devices. **(b)** Quantification of activation markers on CD4 + T cells, **(c)** CD8 + T cells, and **(d)** CD56 + NK cells from uninfected and infected IC-LOC devices. N = 6 samples, with each sample comprised of two pooled devices from the same immune cell donor across at least 2 independent experiments. Statistical significance determined via two-tailed unpaired t-test, with error bars showing S.E.M.

**Fig. 7 |:**
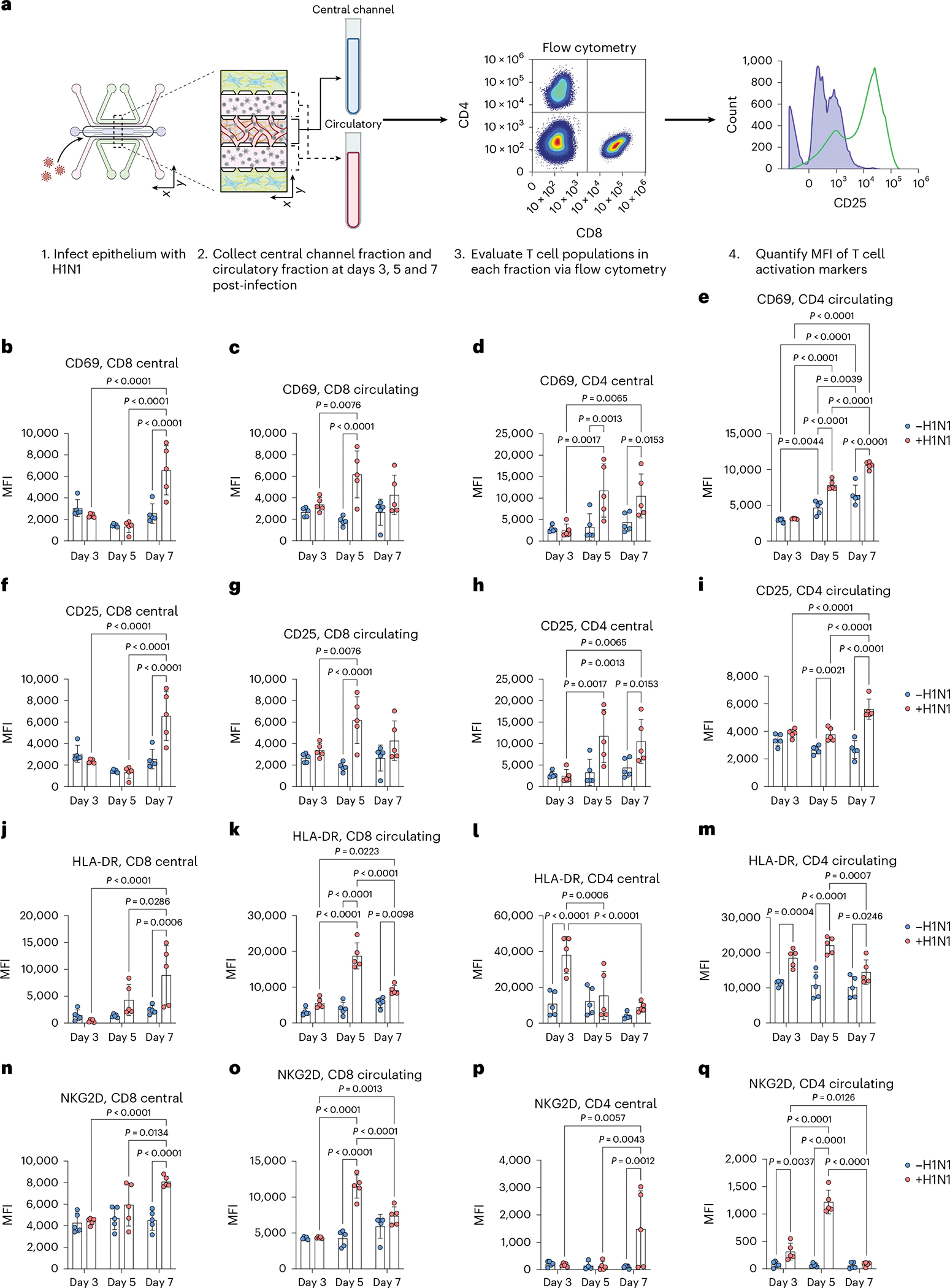
Severe H1N1 infection activates T cells in IC-LOC devices. **a**, Schematic showing experimental set-up, created using BioRender (https://BioRender.com/f8dciz7). **b**–**e**, CD69 expression levels at 3, 5 and 7 days post-infection, as shown by MFI, in central channel CD8 T cells (**b**), circulating CD8 T cells (**c**), central channel CD4 T cells (**d**) and circulating CD4 T cells (**e**) from uninfected and infected IC-LOC devices. *N* = 5 independent devices at each timepoint for each condition. **f**–**i**, CD25 expression levels at 3, 5 and 7 days post-infection, as shown by MFI, in central channel CD8 T cells (**f**), circulating CD8 T cells (**g**), central channel CD4 T cells (**h**) and circulating CD4 T cells (**i**) from uninfected and infected IC-LOC devices. *N* = 5 independent devices at each timepoint for each condition. **j**–**m**, HLA-DR expression levels at 3, 5 and 7 days post-infection, as shown by MFI, in central channel CD8 T cells (**j**), circulating CD8 T cells (**k**), central channel CD4 T cells (**l**) and circulating CD4 T cells (**m**) from uninfected and infected IC-LOC devices. *N* = 5 independent devices at each timepoint for each condition. **n**–**q**, NKG2D expression levels at 3, 5 and 7 days post-infection, as shown by MFI, in central channel CD8 T cells (**n**), circulating CD8 T cells (**o**), central channel CD4 T cells (**p**) and circulating CD4 T cells (**q**) from uninfected and infected IC-LOC devices. *N* = 5 independent devices at each timepoint for each condition. All devices use immune cells from the same immune cell donor. Statistical significance determined via ordinary one-way ANOVA followed by Tukey’s multiple comparisons test with *P* values shown on plots. Data are presented as mean values ± s.d.

We further validated the activation of CD4 T, CD8 T and NK cells via flow cytometry and observed significant increases in activation profiles in response to severe influenza in the IC-LOC (Extended Data Fig. 9). While we observed modest but significant activation of the T cell subsets at 3 days post-infection, we further investigated whether this activation could be sustained over the course of 7 days, as naive T cell activation is typically triggered within the first 3 days of infection, with differentiation and effector function occurring by day 7 and beyond. To this end, we evaluated the expression levels of activation markers CD25, CD69, HLA-DR and NKG2D in both CD4 and CD8 T cell populations collected from the media channels (denoted as ‘circulatory’) or the degraded central channel (denoted as ‘central’) of both uninfected and infected IC-LOC devices at 3, 5 and 7 days post-infection (Fig. 7a and Supplementary Fig. 18). With this, we found that while activation of the CD8 T cell populations was indeed initiated at 3 days post-infection with significantly elevated levels of CD69 (Fig. 7b,c), expression of CD25 (Fig. 7f,g), HLA-DR (Fig. 7j,k) and NKG2D (Fig. 7n,o) continued to increase at 5 and 7 days post-infection. In addition, we observed that CD4 T cells in the central channel exhibited a nearly 3-fold increase in HLA-DR expression 3 days post-infection (Fig. 7l,m), followed by 2- to 3-fold increases in CD69 (Fig. 7d,e) and CD25 (Fig. 7h,i) by days 5 and 7. While CD4 T cells in the circulating fraction also showed significant increases in CD25, CD69 and HLA-DR, they did not reach the magnitude of those in the central channel, thereby highlighting a unique capability of our IC-LOC system to distinctly evaluate the activation of cells residing within the interstitial (‘central’) regions of the lung device and the more distant ‘circulatory’ fraction.

**Extended Data Fig. 10 |:**
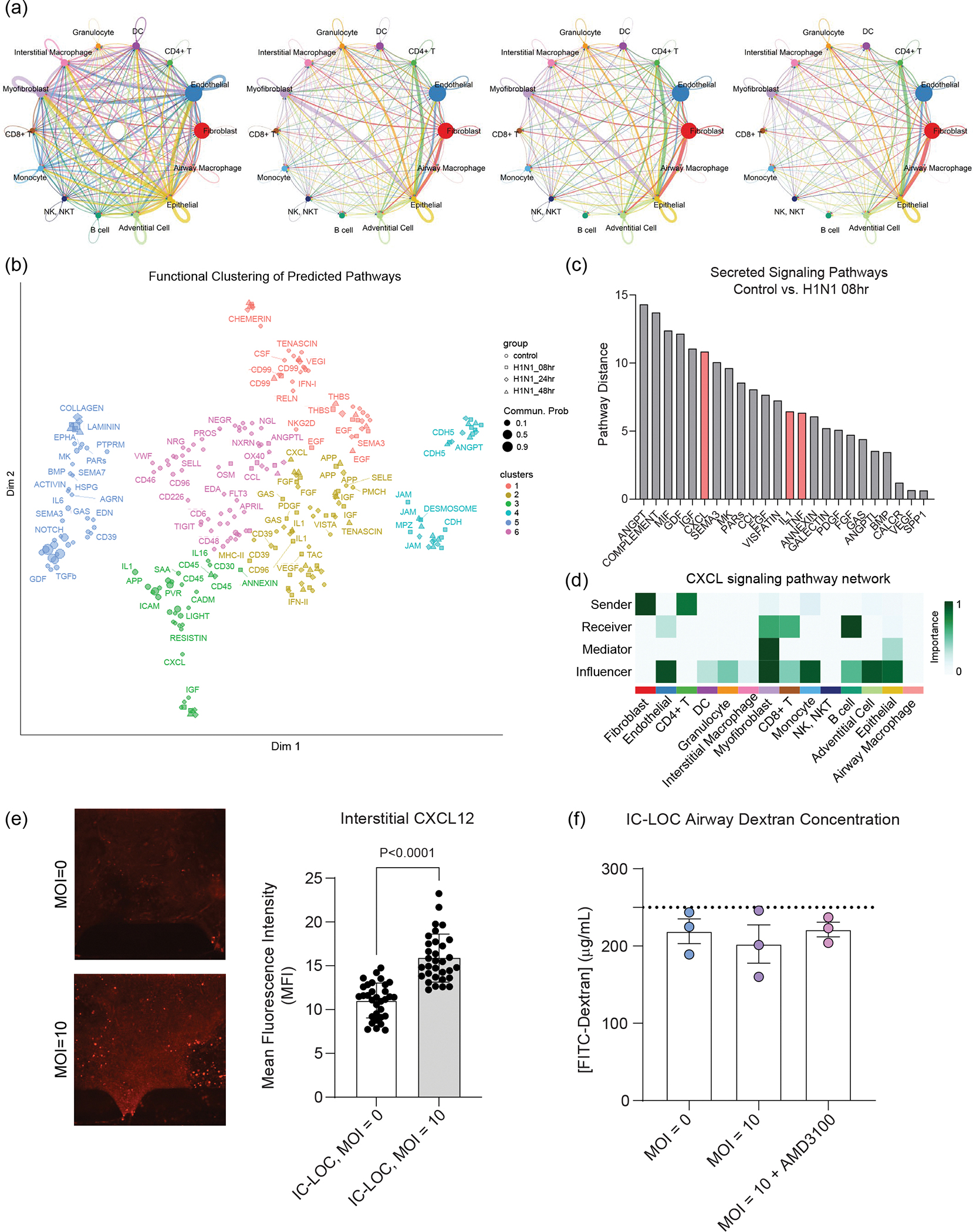
Dysregulated CXCL signaling in the IC-LOC in response to severe H1N1 infection. **(a)** Circle plots showing all CellChat predicted cellular interactions in the control, 08 hr H1N1, 24 hr H1N1, and 48 hr H1N1 samples. Weight of line indicates number of interactions. **(b)** Functional clustering of predicted pathways from CellChat analysis of scRNA-sequencing data of healthy (control) and infected (08 hr, 24 hr, 48 hr) IC-LOC devices. Pathways with similar functions, based on gene expression patterns, are grouped and colored together into 6 distinct clusters. **(c)** The calculated Euclidean distance between the control and 08 hr infected samples in the functional clustering map for all secreted signaling pathways. **(d)** Signaling patterns in the CXCL pathway in the 08 hr H1N1 IC-LOC sample, showing signaling arising largely from the fibroblast population signaling mainly to myofibroblasts, CD8 T cells, and B cells. **(e)** Representative images and quantification of interstitial CXCL12 expression levels in the uninfected (MOI = 0) and infected (MOI = 10) IC-LOC. N = 30 ROIs from 3 independent devices from the same immune cell donor. Statistical significance determined via two-tailed unpaired t-test with Welch’s correction and error bars showing S.D. **(f)** The dextran permeability for IC-LOC devices that were uninfected, infected at MOI 10, or infected at MOI 10 and also treated with the CXCR4 inhibitor AMD3100. N = 3 independent devices from the same immune cell donor.

In addition to gene expression analysis, we utilized CellChat analysis techniques to evaluate the change in predicted cellular communication and cell–cell interactions in response to severe H1N1^49^. In the healthy state, the primary interactions were between myofibroblasts, IMs, adventitial cells, endothelial cells and epithelial cells (Fig. 6g and Extended Data Fig. 10a). Upon H1N1 infection, interactions with fibroblasts, CD4 T cells, granulocytes and DCs increased dramatically, as can be seen in Fig. 6g, with the top 10% of predicted interactions shown and line thickness corresponding to the number of predicted interactions. Notably, we again observe the significant role of stromal cells, namely fibroblasts, in severe influenza in the IC-LOC.

### CXCR4, IL-1β and TNF-α inhibition alters severe influenza

**Fig. 8 |:**
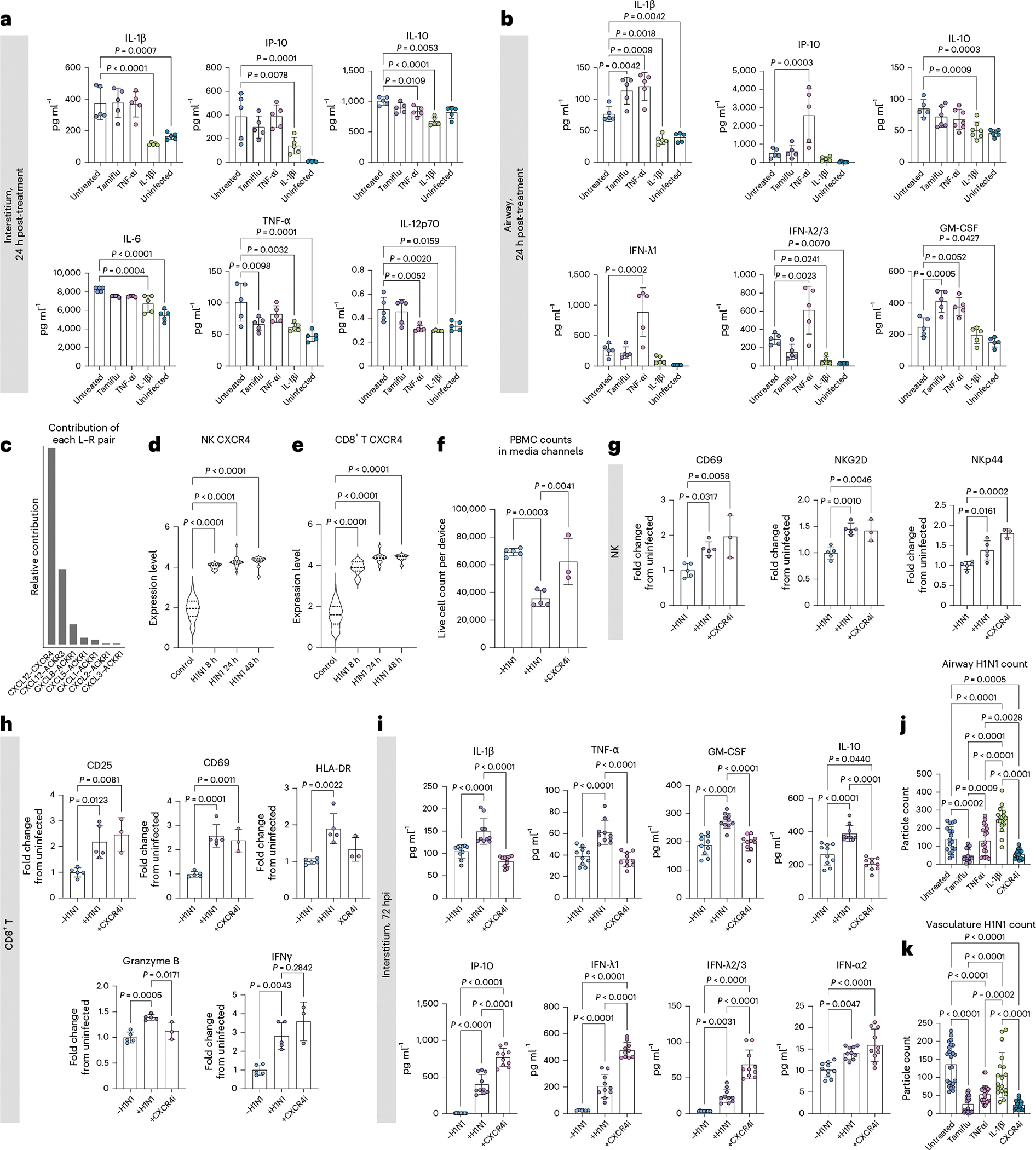
Modulating the immune activation and cytokine response to severe influenza. **a**,**b**, Cytokine release profiles in the interstitium (**a**) and airway (**b**) of IC-LOC devices in response to infection and treatment with Tamiflu, IL-1βi (canakinumab) or TNF-αi (infliximab). *N* = 5 independent devices. **c**, CellChat predicted L–R interactions comprising the CXCL pathway, with CXCL12–CXCR4 appearing as the key L–R pair. **d**,**e**, CXCR4 expression levels in CXCR4^+^ NK cells (**d**) and CD8 T cells (**e**) in uninfected and infected devices, determined from scRNA-seq data. Significance determined via one-way ANOVA followed by Kruskal–Wallis test. **f**, Flow cytometry quantification of live PBMC cell counts per device in the circulatory fraction of IC-LOC devices in response to H1N1 infection and treatment with CXCR4 inhibitor AMD3100. *N* = 5 samples for ±H1N1 and *N* = 3 samples for AMD3100, with each sample comprised of 3 pooled IC-LOC devices. **g**,**h**, Activation phenotypes of NK cells (**g**) and CD8 T cells (**h**) in response to influenza infection and CXCR4 inhibition with AMD3100. Marker expression plotted as fold change from uninfected devices. *N* = 5 samples for ±H1N1 and *N* = 3 samples for AMD3100, with each sample comprised of 3 pooled IC-LOC devices. **i**, Cytokine release profiles in the interstitium of IC-LOC devices in response to infection and treatment with AMD3100. *N* = 10 independent devices across at least 2 independent experiments. **j**,**k**, Quantified viral levels as determined via particle count from confocal fluorescence microscopy in the airway (**j**) and interstitium (**k**) of infected devices left untreated or treated with Tamiflu, IL-1βi (canakinumab), TNF-αi (infliximab) or CXCR4i (AMD3100). *N* = 18 ROIs from 3 independent devices. All devices use immune cells from the same immune cell donor. Statistical significance determined via ordinary one-way ANOVA unless otherwise specified. Data are presented as mean values ± s.d.

We then used the joint manifold learning capabilities of CellChat to cluster the significant pathways by function and observed six distinct clusters (Extended Data Fig. 10a). We computed the Euclidean distance between control pathways and their 8 h H1N1 counterparts to quantify the functional difference between pathways from uninfected and infected samples, with a larger Euclidean distance indicative of increased dysfunction (Extended Data Fig. 10b,c). Through this analysis, we found that the TNF and IL-1 pathways were implicated in the sequencing data as dysregulated pathways in response to H1N1 (Extended Data Fig. 10c), in agreement with the cytokine secretion data (Fig. 2j). Thus, we chose to evaluate TNF-α and IL-1β inhibitors for their ability to ameliorate the cytokine storm associated with severe influenza. To mimic the typical in vivo timeline of antiviral administration, all therapeutics were administered to the devices 24 hpi. After 24 h of treatment (48 hpi), media was collected from the interstitial and airway layers of the device, and the secreted cytokine landscape was quantified via LEGENDplex multiplexed cytokine analysis. We found that the IL-1β inhibitor, canakinumab (10 μg ml^−1^), was uniquely able to control the hyperinflammation following infection, significantly reducing cytokine levels compared with untreated devices and, in most cases, returning the levels back to that of the uninfected devices (Fig. 8a,b). While administration of the TNF-α inhibitor, infliximab (10 μg ml^−1^), had a modest anti-inflammatory effect in the interstitium, it resulted in significant spikes in the airway layer in antiviral response cytokines CXCL10, IFN-λ1 and IFN-λ2/3 and pro-inflammatory IL-1β, further contributing to the hyperinflammatory environment (Fig. 8a,b). We also wondered whether the antiviral oseltamivir (Tamiflu), approved for seasonal flu by United States Food and Drug Administration, could mitigate the associated inflammatory response in severe influenza. Notably, we found that administering Tamiflu (100 μM) to severe influenza IC-LOC devices had little to no beneficial effect on the observed cytokine response, even increasing levels of IL-1β and GM-CSF in the airway (Fig. 8a,b).

Notably, among the signalling pathways canonically associated with inflammation, it was the CXCL pathway that had the largest predicted dysregulation between the control and 8 h H1N1 samples (Extended Data Fig. 10c). Thus, we investigated the specific signalling occurring in this pathway. When considering the specific ligand–receptor (L–R) interactions, we found that the CXCL12–CXCR4 interaction had the largest relative contribution in the CXCL pathway, coming largely from fibroblast CXCL12 (Fig. 8c and Extended Data Fig. 10d). CXCR4 is known to be involved in leukocyte recruitment and, in epithelial cells, has been shown to regulate mucosal host defence^69–71^. We evaluated CXCL12 expression levels in the interstitium of the IC-LOC via confocal microscopy and found a significant increase in response to severe influenza (Extended Data Fig. 10e). We then probed CXCR4 expression levels across adaptive immune populations and found that in the cells expressing CXCR4, the expression levels in CD8 T and NK cells were significantly upregulated in response to infection, according to scRNA-seq analysis (Fig. 8d,e). Thus, we hypothesized that this axis may play a role in recruiting leukocytes from the media channels into the central channel of infected devices. To evaluate this, we treated devices with a CXCR4 inhibitor, AMD3100 (1 μg ml^−1^), 1 h before infection. At the endpoint (72 hpi), we collected all circulating immune cells from the media channels and found that while H1N1 infection significantly decreased the numbers of circulatory immune cells in the media channels, indicative of an increase in migration into the central channel as seen in Fig. 4j,k, treatment with AMD3100 restored this number nearly back to uninfected levels (Fig. 8f).

We also evaluated the effect of CXCR4 inhibition via AMD3100 on the activation state of the collected PBMCs through flow cytometric analysis. We found that while H1N1 infection resulted in significant increases in activation markers CD25, CD69, HLA-DR, granzyme B, IFNγ and NKp44 on CD8 T and NK cells, the addition of AMD3100 did not reduce a majority of these markers except HLA-DR and granzyme B in CD8 T cells (Fig. 8g,h). Thus, it appears that while this axis plays a prominent role in immune cell migration, it does not majorly impact the activation state of canonical effectors, CD8 T and NK cells. We further probed the effect of inhibiting this axis on the resultant cytokine storm following severe influenza infection. We observed that inhibition of CXCR4 resulted in a decrease in inflammatory and regulatory cytokines (IL-1β, TNF-α, IL-10 and GM-CSF) and an increase in antiviral-associated cytokines (CXCL10, IFN-λ1, IFN-λ2/3 and IFN-α2) (Fig. 8i). Thus, CXCR4 inhibition could be a potential therapeutic target in the treatment of severe influenza, as it does not hamper the necessary activation of effector immune cells (CD8 T and NK) and increases the antiviral cytokine response while simultaneously decreasing levels of cytokines associated with tissue damage, namely IL-1β, TNF-α and GM-CSF.

To evaluate the potential of each inhibitor to reduce viral load, we then quantified the detectable levels of viral particles in the airway and interstitial regions of the devices. We found that while Tamiflu did not reduce cytokine levels, it significantly reduced the viral levels in both layers of the device (Fig. 8j,k). TNF-α inhibition also significantly reduced viral levels in the interstitium but had no effect in the airway layer of the device. Meanwhile, IL-1β inhibition did not significantly affect viral levels in the interstitium but significantly increased the observed viral level in the airway, compared with untreated devices. Thus, while IL-1β inhibition effectively ameliorates the observed cytokine storm, it may inhibit the immune response too severely and lead to increased viral presence. When we evaluated the viral levels in devices treated with the CXCR4 inhibitor AMD3100, we found that viral levels in the epithelial and interstitial regions of the device were significantly reduced to levels similar to those resulting from Tamiflu treatment (Fig. 8j,k). This is surprising, as CXCR4 does not directly interfere with viral entry or replication as Tamiflu does. We hypothesize that the significant drop in viral levels is instead a consequence of the significantly increased antiviral cytokines secreted in response to AMD3100 treatment and not a result of CXCR4i-induced epithelial cell death, as the treatment with AMD3100 did not significantly impact the epithelial permeability in the IC-LOC (Extended Data Fig. 10f).

Taken together, we demonstrate the unique roles of IL-1β, TNF-α and CXCR4 in response to severe influenza infection. TNF-α appears critical for the regulation of the epithelial cytokine response as its inhibition results in dramatic spikes in inflammatory and antiviral cytokine secretion. Conversely, IL-1β may be a driver of the hyperinflammatory response to severe influenza, as its inhibition results in the amelioration of the cytokine storm both in the interstitium and airway of the IC-LOC. Meanwhile, we find that CXCR4 is critical for the migration of circulatory immune cells into inflamed tissues and its inhibition significantly modulates the cytokine landscape of the inflamed tissue. In fact, CXCR4 inhibition is unique in its ability to decrease the pro-inflammatory cytokine profiles while increasing the antiviral response, in turn decreasing viral levels and arising as a potential therapeutic target.

## Discussion

Here we have demonstrated the development of an immune-competent, microvascularized, human LOC device to model the small airways. The modular design of the developed LOC allows immune components to be easily included or excluded depending on the desired application. We generated three distinct models, the LOC, Mϕ-LOC and IC-LOC, to investigate the role of various immune populations in the context of severe influenza. By using immune cells from a single donor, we could mitigate non-self-activation in the IC-LOC, as evidenced by low baseline cytokine release profiles and minimal levels of T cell activation markers, and maintain high immune cell viability in the device. Furthermore, through the investigation of the three distinct models, we found that tissue-resident macrophages were necessary to protect the structural components of the LOC. Still, the incorporation of circulating immune components was necessary to recapitulate the cytokine storm canonically associated with severe influenza within the BAL fluid and serum.

We performed scRNA-seq on the IC-LOC at various timepoints of infection to gain a more thorough understanding of the cellular dynamics and gene expression patterns in response to severe influenza infection. Our observations within the IC-LOC aligned with known phenomena in patients with severe influenza, including immune cell infiltration and death, innate and adaptive immune activation, and aberrant fibrosis. Specifically, we demonstrate a prominent antiviral response in both innate and adaptive immune populations. We also highlight the role of the stromal components of the lung during severe infection and demonstrate their importance in the antiviral response, ECM remodelling and immune cell recruitment.

While our bioengineered IC-LOC incorporates many components of the in vivo lung, including a microvascular network, stromal cells, tissue-resident and circulatory immune cells, and an epithelial monolayer at ALI, there are still components of the in vivo lung that are lacking and phenomena that we are currently unable to study in this platform. Specifically, our system lacks the native plasma, platelets and RBCs and thus cannot study microcoagulation or thrombocytopenia in response to H1N1 infection^72,73^. In addition, while we have incorporated both innate and adaptive immune cells and have demonstrated a functional antiviral response, the IC-LOC system lacks a true secondary lymphoid organ structure^74,75^, and thus responses such as germinal centers, affinity maturation of B cells and antibody generation are likely hindered. Our system also uses HUVECs as the endothelial cell source, which, while optimal for generating spontaneous vascular networks, may slightly differ in their response to severe influenza when compared with pulmonary endothelial cells. In addition, the stability of the microvascular network limits the length of studies that can be performed in the device, as the network overgrows with extended culture times. Thus, the chosen experimental timeline balances the stability of the vascular network with the maturation of the airway epithelium. Furthermore, the IC-LOC system is comprised of a fixed number of immune cells, and thus, while we observe immune cell infiltration in response to infection, the magnitude is limited, and the resulting immune cell death may be exaggerated as there are no new infiltrating cells to replace those that have died. Lastly, as the goal was to develop and validate a fully immune-competent LOC model of severe influenza, all experiments performed herein utilize immune cells from a single donor to ensure reproducibility in the complex IC-LOC device. Evaluation of additional donors would be necessary to extrapolate these findings to a broader biological context, especially as they relate to the response to potential therapeutics. Now that the system has been validated in its ability to model severe influenza, the IC-LOC could be utilized to investigate the impact of donor variability, including how differences in age, race, gender and underlying conditions impact the observed responses to severe influenza.

Herein we demonstrate the use of the developed IC-LOC. Specifically, this model offers a multitude of advantages in the study of the lung microenvironmental response to infection: the incorporation of tissue-resident and circulating immune components allows for a more complete immune system mimic, the microvascular network allows for elucidation of perfused immune cell interactions and extravasation in a physiologically relevant environment, and the cell-permeable membrane allows for immune cell trafficking of viral particles from the epithelium. Furthermore, we demonstrate the importance of interstitial components, such as the IMs and stromal cells, which are often overlooked in in vitro LOC models when studying the response to H1N1 infection. Through insights from scRNA-seq and subsequent experimental validation, we demonstrate the unique roles of IL-1β, TNF-α and CXCR4 in response to influenza and highlight CXCR4 as a potential therapeutic target. This immune response model paves the way for future explorations of the human lung-immune microenvironment in vitro.

## Methods

### Ethics oversight

The research conducted herein complies with relevant ethical regulations from Emory University’s institutional review board-approved protocol ‘Phlebotomy of Healthy Adults for the Purpose of Evaluation and Validation of Immune Response Assays’ (IRB00045821). Blood for primary immune cell isolation was acquired from informed donors and researchers were blinded to donor identity.

### Microfluidic device fabrication

The LOC device is comprised of two layers of PDMS (Dow Corning, catalogue number 2065622) with a 5.0 μm PETE membrane (Sterlitech, catalogue number PET5025100) sandwiched in between. The vascular layer of the device was fabricated using a previously developed^34^ AutoCAD design that has been slightly modified to increase the ease of handling and imaging. In brief, a 150 mm silicon wafer was spin-coated with SU-8 photoresist at a height of approximately 150 μm. The Heidelberg 150 Maskless Aligner was then used to expose the pattern onto the photoresist and the wafer was developed. From the wafer, multiple SmoothCast moulds were made to increase throughput and protect the fragile wafer from repeated use. The airway layer of the device was also fabricated using a previously developed^34^ SOLIDWORKS design that has been slightly modified to increase ease of fabrication. The SOLIDWORKS design was then 3D printed and, as with the vascular design, multiple SmoothCast moulds are made. PDMS, used at a 10:1 base/curing agent ratio by weight, was then poured onto the moulds and cured at 65 °C for at least 2 h.

The device was fabricated on the back of a black bottomless 96-well plate (Sigma-Aldrich, catalogue number 448931), allowing for the fabrication of 8 independent devices at once. First, the airway layer was plasma bonded to the bottom of the 96-well plate treated with 2% (v/v) 3-mercaptopropyltrimethoxysilane (Sigma-Aldrich, catalogue number 175617) in methanol (EMD Millipore, catalogue number MX0490-04). The airway layer bonded plates were put in a 65 °C oven overnight before the membranes and the vascular layer were plasma bonded to the airway. Before incorporation, the PETE membranes were plasma treated and immersed in a 5% APTES solution (Sigma-Aldrich, catalogue number 440140) for 10 min. Upon plasma bonding the membranes and vascular layer, the completed device was put into a 65 °C oven to cure overnight.

Before loading cells into the devices, the PETE membranes were coated to permit epithelial cell adhesion. In brief, a 0.1% dopamine-HCl (Sigma-Aldrich, catalogue number 175617) solution was made in Tris-HCl pH 8.8 (Bioworld, catalogue number 42020414-2). This solution was added to the airway channel to coat the membrane for 1 h. The solution was then removed, the membrane was washed with 1× PBS (Cytiva, catalogue number SH30256), and a 100 μg ml^−1^ Type I Rat Tail Collagen (Corning, catalogue number 354249) solution in PBS was added to the airway channel. This was also allowed to incubate for 1 h before being removed. The membranes were then washed a final time with 1× PBS, and the devices were put into a 65 °C oven to dry before loading cells.

### Fluorescent labelling of cells

To monitor cellular localization within the devices and identify distinct populations, some cells were labelled with membrane labels, including CellTracker Green and CellTrace Violet Fluorescent Probes (Thermo Fisher). To this end, cells were collected through incubation with TryPLE if adherent and centrifuged for 5 min at 300 × *g* to remove cell culture media. The resulting pellet was resuspended in CellTracker solution (20 μM) and incubated for 30 min at 37 °C. The cells were then centrifuged again (300 × *g*, 5 min) and the pellet was resuspended in cell culture media to wash away any unbound fluorescent probe. The cells were centrifuged (300 × *g*, 5 min) and the labelled cells were resuspended in media and used for downstream applications.

### In vitro cell culture in LOC devices

The cell types utilized within the LOC device include endothelial cells, epithelial cells, fibroblasts, macrophages, DCs and white blood cells. The endothelial cells used were primary HUVECs. Specifically, pooled donor HUVECs (Lonza, catalogue number C2519A) were used to minimize donor-to-donor variability. Primary SAECs (Lonza, catalogue number CC-2547S) were used in the epithelial layer of the device. Primary normal human lung fibroblasts (NHLFs; Lonza, catalogue number CC-2512) from two different donors (NHLF-B and NHLF-E) were used in the vascular layer, according to previously performed donor optimization. HUVECs, SAECs and NHLFs were all acquired from Lonza. The macrophages were differentiated from fresh whole blood. In brief, PBMCs were isolated from whole blood using density centrifugation with Ficoll (Cytiva, catalogue number 17-1440-02) and RBC lysis with RBC lysis buffer (Thermo Scientific, catalogue number 00-4333-57). The resulting PBMCs were plated in a tissue culture flask and monocytes were allowed to adhere overnight. All suspension cells were then removed from the flask and the adherent monocytes were differentiated to macrophages with 50 ng ml^−1^ M-CSF (PeproTech, catalogue number 315-02) for 7 days. DCs were differentiated similarly, with blood-derived monocytes differentiated to DCs using 800 U ml^−1^ GM-CSF (PeproTech, catalogue number 300-03) and 1,000 U ml^−1^ IL-4 (PeproTech, catalogue number 200-04) for 7 days. Lastly, PBMCs perfused through the vasculature were also acquired from whole blood using the above isolation techniques.

Seeding cells within the LOC device is a multi-day process. On day 0, the vascular layer was seeded. The two outermost channels were seeded with NHLF-Es at a concentration of 3 × 10^6^ ml^−1^ in a 5 mg ml^−1^ fibrin gel (Sigma-Aldrich, catalogue number F3879-250MG). The central channel was seeded with a mixture of HUVECs, NHLF-Bs, macrophages and DCs at concentrations of 6 × 10^6^ ml^−1^, 0.3 × 10^6^ ml^−1^, 0.3 × 10^6^ ml^−1^ and 0.3 × 10^6^ ml^−1^, respectively, in a 0.5 mg ml^−1^ collagen, 4.5 mg ml^−1^ fibrin gel. The devices were fed with Endothelial Growth Medium (EGM-2MV; Lonza, catalogue number CC-3162) supplemented with 50 ng ml^−1^ VEGF (PeproTech, catalogue number 100-20). On day 2, SAECs were seeded in the airway channel at a concentration of 2.5 × 10^6^ ml^−1^ and the media was changed to EGM-2MV supplemented with 50 ng ml^−1^ VEGF and 100 ng ml^−1^ Ang-1 (PeproTech, 130-06). On day 4, the airway epithelium was airlifted, and the media was changed to a 1:1 mixture of Pneumacult-ALI media (STEMCELL Technologies, catalogue number 05001) and EGM-2MV with 50 ng ml^−1^ VEGF and 100 ng ml^−1^ Ang-1. On day 7, AMs were seeded on top of the epithelial monolayer at a concentration of 1 × 10^6^ ml^−1^. On day 9, PBMCs or RBC-depleted whole blood was added to the media channels at concentrations of 1 × 10^6^ ml^−1^ or 2 × 10^6^ ml^−1^, respectively, to mimic blood concentrations of these cells, and media was supplemented with 100 IU ml^−1^ IL-2. The devices were kept in culture until day 12 and fed every 2 days.

### Viral labelling

Live virus was acquired from ATCC (Influenza A/PR/8/34, VR-1469) at a stock concentration of 1.10 × 10^9^ viral particles per ml. Live virus was labelled as described by Balogh et al.^76^. In brief, 0.5 μl of PKH26 dye (Sigma-Aldrich, catalogue number MIDI26-1KT) was dissolved in 1 ml of Diluent C immediately before labelling. Two volumes of diluted PKH26 was added to 1 volume of virus suspension in PBS and rapidly mixed by pipetting. After 3 min of incubation, an equal volume of 1% BSA (R&D Systems, catalogue number RB02) was added and incubated for 1 min to quench the excess dye. At this point, 10 μl sample was taken for observation under a fluorescent microscope. The viral solution was then centrifuged at 20,000 × *g* for 20 min to pellet the virus. The supernatant was removed, and the pellet was washed with 1 ml PBS. Again, a 10 μl sample was taken for observation under a fluorescent microscope. The viral solution was centrifuged once more to pellet the virus (20,000 × *g*, 20 min), and the pellet was resuspended to a final volume equal to that of the starting volume and a final 10 μl sample was taken to validate fluorescence of viral particles before addition to LOC devices.

### Viral infection

On day 9 of culture after the addition of the AMs, the devices were infected with H1N1 (Puerto Rico Strain A/PR/34/8). Specifically, the epithelial layer was incubated for 2 h at 37 °C with H1N1 in 1× PBS at an MOI of 10. After 2 h, the inoculant was removed, and the channels were gently rinsed with 1× PBS before the devices were returned to the incubator for culture.

### Fluorescence microscopy

At the endpoint of a given experiment (days 12–16), devices were fixed using 4% paraformaldehyde (PFA) (Thermo Fisher, catalogue number 28906) and permeabilized with 0.1% Triton X-100 (Sigma-Aldrich, catalogue number X100-100ML). Specifically, 100 μl of 4% PFA was added to each media channel and 10 μl was added to the airway channel and allowed to incubate for 15 min at room temperature. The PFA was then removed, devices were rinsed with 1× PBS, and 0.01% Triton X-100 was added and allowed to incubate for 15 min at room temperature. Upon fixation and permeabilization, the devices were blocked with 5% bovine serum albumin (BSA) for at least 2 h at room temperature. After blocking, primary antibodies were added to the devices at a concentration of 1–5 μg ml^−1^, with 30 μl added to each media channel and 10 μl added to the airway channel. The devices were then allowed to incubate overnight at 4 °C, protected from light. The following day, the devices were rinsed 3 times with 0.1% BSA and imaged on a PerkinElmer spinning disk confocal microscope using Volocity Imaging Software (version 6.3). All antibodies used are listed in Supplementary Information.

### Image analysis

Image processing was performed using Volocity Software (version 6.3) and all images were processed in batch according to the experiment to ensure consistent background and brightness/contrast adjustment across all images. Image analysis was performed using AngioTool (version 0.5) and ImageJ (version 1.54). AngioTool was used for all analyses of the microvasculature, including percent area covered, average vessel length and average vessel diameter. For co-localization analysis, the ImageJ plugin Coloc2 was used. For particle counts and percent area quantifications (except those for vasculature), images were first thresholded before running Particle Analysis in ImageJ to determine both the number of particles and area covered. As with Volocity image processing, all image analysis was performed in batch to ensure consistent metrics across groups.

### Multiplexed FISH for H1N1 viral RNA

We performed the third-generation hybridization chain reaction (HCR) for RNA-FISH following the HCR mammalian cells on slides protocol provided by Molecular Instruments. The samples were first fixed with 4% PFA (10 ml 16% PFA, 4 ml 10× PBS, 26 ml ultrapure water for 40 ml of fixation buffer) at room temperature for 15 min and washed with 1× PBS. The samples were then permeabilized in 70% ethanol at −20 °C for over 12 h.

After permeabilization, the samples were air-dried for 10 min and washed with 2× SSC buffer 3 times. The samples were then incubated in a 300 μl pre-warmed HCR probe hybridization buffer at 37 °C for 30 min for prehybridization. HCR probes (3 μl) for desired targets were then added to 300 μl of pre-warmed probe hybridization buffer. The prehybridization buffer was aspirated, and the probe dilute was added to the samples. The samples were incubated at 37 °C for 12–16 h. The samples were then washed with 300 μl of pre-warmed probe wash buffer for 5 min at 37 °C 4 times. Then, the samples were rinsed with 300 μl of 5× SSCT for 5 min at room temperature twice. The samples were then incubated with 300 μl of amplification buffer at room temperature for 30 min for pre-amplification. Each HCR amplifier hairpin (6 μl) was then snap-cooled to 95 °C for 90 s and cooled to room temperature in a dark drawer for 30 min. The snap-cooled hairpins were then added to 300 μl of amplification buffer. The pre-amplification buffer was then aspirated, and diluted hairpins were added to the sample. The samples were then incubated for 1 h and 15 min. The amplification solution was then aspirated, and the samples were washed with 5× SSCT for 5 min at room temperature 5 times (see HCR protocol for cells on slide).

The samples were then mounted in an antifade mounting buffer containing Tris-HCL (20 mM), NaCl (50 mM), glucose (0.8%), saturated Trolox (Sigma, 53188-07-1), pyranose oxidase (Sigma, P4234) and catalase (Sigma, 9001-05-2, 1:1,000 dilution).

To remove fluorescent mRNA signals, we used RNase-free DNase I (Sigma, 04716728001). After imaging, the samples were first washed with 2× SSC twice. We then diluted 50 μl of 10× concentration incubation buffer in 450 μl of RNase-free water to 1× concentration. The samples were then incubated with a 1× incubation buffer at room temperature for 5 min. Then 10 μl of DNase I, 50 μl of 10× incubation buffer and 440 μl of RNase-free water were mixed. The samples were then incubated in the DNase I mixture for 4 h at room temperature. After incubation, the samples were then washed with 30% formamide in 2× SSC at room temperature for 5 min 3 times. Removal of signals was confirmed under a fluorescent imaging microscope. The samples were then ready to start the next cycle of RNA labelling.

After the removal of FISH signal by DNase, CD68 staining was performed to identify macrophages in the epithelial layer. The CD68 antibody used in this study is Alexa Fluor conjugated kp1 clone (Santa Cruz sc-20060). The antibody was diluted at 1:50 in cell staining medium containing 0.5% BSA and 0.02% sodium azide in PBS. The samples were incubated in the cell staining medium for 30 min at room temperature for blocking. The samples were then incubated in antibody dilution at 4 °C overnight. The samples were then washed with 1× PBST (0.1% Tween20 in 1× PBS) 3 times, 5 min each. The samples were then imaged at the same positions as previous images.

The captured images were then background-subtracted using the rolling ball method implemented in Fiji1. The autofluorescence signal was then removed using a top-hat filter implemented in the scikit-image2. The FISH images and IF images were then cross-registered to achieve pixel-level alignment using phase cross-correlation methods implemented in the scikit-image2. A threshold was manually identified for the FISH images to distinguish FISH signal dynamic range and the background dynamic range. After the threshold was applied to the images, each contiguous region was identified as a copy of the viral transcript. The cell nuclei were then segmented using cellpose3, and the nuclei masks were expanded to find the cell mask using CellProfiler4. The viral copy number of each cell was counted by combining the cell masks and the positions of detected viral transcripts.

### Collecting cell lysis from LOC devices

Cell lysis was collected from lung chip devices to quantify viral levels. To this end, at the infection endpoint (72 hpi), the media from the media channels was collected and cells from the devices were lysed using water lysis by incubating devices in H_2_O for 15 min. The resulting cell lysis was collected and pooled with the collected media. The mixture was centrifuged at 500 × *g* for 5 min to pellet any cellular debris and the viral-containing supernatant was collected.

### Viral ToxGlo assay

To quantify relative levels of infectious virus, we utilized the luminescent Viral ToxGlo Assay from Promega. The standard protocol was followed. In brief, MDCK cells were seeded in Dulbecco’s modified Eagle medium (DMEM) with 10% FBS at 10,000 cells per well in a 96-well plate and allowed to reach confluence (48 h). The media was then replaced with 100 μl of DMEM supplemented with 1 μg ml^−1^ of TPCK-trypsin. The collected virus solution from the devices (as described above) was added in triplicate at a 1:5 dilution and half-log serial dilutions were performed to generate an 8-point dilution curve. The cells were incubated for 72 h to allow for cytopathic effects to take place. At 72 hpi, 100 μl of the ATP Detection Reagent was added to each well of the 96-well plate. The plate was then placed on a plate shaker at 400 rpm for 10 min to ensure proper mixing. After 10 min, the liquid was transferred from the tissue culture plates into opaque white plates and luminescence readings were obtained using a Synergy HTX plate reader and Agilent BioTek Gen5 software (version 3.07). No-cell controls were included, and those readings were used for background subtraction.

### HA ELISA

Viral antigen HA was measured in the cell lysis solution using an Influenza A H1N1 (A/PR/8/1934) Hemagglutinin HA ELISA kit (Sino Biological), following the provided protocol. In brief, 100 μl of standard or sample was added to the provided 96-well plate in duplicate and the plate was sealed and allowed to incubate for 2 h at room temperature. The wells were then washed 3× with wash buffer and 100 μl of detection antibody was added to each well. The plate was sealed and incubated for 1 h at room temperature. The wells were washed 3× with wash buffer and 100 μl of substrate solution was added to each well and incubated for 20 min at room temperature, protected from light. Finally, 100 μl of Stop solution was added to each well and the plate was gently tapped to mix. The optical density of each well at 450 nm was immediately read using Synergy HTX plate reader and Agilent BioTek Gen5 software (version 3.07).

### LDH activity assay

To determine LDH levels within the devices, we utilized the CyQUANT LDH Cytotoxicity Assay from Thermo Fisher. The standard protocol was followed. In brief, 50 μl samples from the lung chip devices were plated in triplicate in black, clear-bottom 96-well plates and 50 μl of the reaction mixture was added to each. Triplicate wells of LDH positive control and blank cell culture media (negative control) were also included. The wells were incubated at room temperature for 30 min, protected from light. Then, 50 μl of Stop solution was added to each well and was mixed by gently tapping the plate. The absorbance at 490 nm and 680 nm was measured using the Synergy HTX plate reader and Agilent BioTek Gen5 software (version 3.07). LDH activity was determined by subtracting the 680 nm absorbance values from the 490 nm absorbance.

### 10 kDa FITC-dextran epithelial permeability assay

To determine the epithelial permeability in the lung chip devices, we utilized a 10 kDa FITC-dextran permeability assay. To this end, 10 μl of 10 kDa FITC-dextran at 250 μg ml^−1^ in 1× PBS was added to the epithelium and allowed to incubate for 2 h at 37 °C, 5% CO_2_. After 2 h, the liquid was collected from the airway channels, added to 90 μl of PBS (10-fold dilution) and split between 2 wells of a 96-well plate to run in duplicate (50 μl per well). A 12-point standard curve was also run in duplicate. The absorbance at 490 nm was measured using the Synergy HTX plate reader and concentrations of the lung chip samples were calculated using the standard curve.

Apparent permeability coefficients were calculated by assuming conservation of mass to estimate FITC-dextran concentrations in the basolateral compartment. Explicitly, measurements of FITC-dextran in the epithelial chamber were taken after the 2 h incubation and the change in concentration was assumed to be due to FITC-dextran transport into the basolateral chamber. In this way, we estimated the FITC-dextran flux (1) and calculated the apparent permeability (2).

(1) $$\frac{dQ}{\;dt}=\frac{\Delta C_{b}\times V_{b}}{\Delta t}$$

(2) $$P_{app}=\frac{\frac{dQ}{\;dt}}{A\times C_{0}}$$

where $C_{b}$ is the estimated basolateral concentration, $V_{b}$ is the basolateral volume (10 μl), A is the membrane area (0.01 cm^2^), $C_{0}$ is the initial dextran concentration (250 μg cm^−3^) and Δt is the incubation time (2 h).

### SEM

Membranes were extracted from devices and fixed with 2.5% glutaraldehyde (Electron Microscopy Sciences, catalogue number 15960) overnight at 4 °C. Following fixation, membranes were rinsed thoroughly with water and stained with 1% osmium tetrachloride (Sigma-Aldrich, catalogue number 251755-2ML) for 30 min at room temperature. Stain was rinsed (4 times) and samples were treated with 0.1 M thiocarbohydrazide (TCI Chemicals, catalogue number T1136-5G) for 20 min at room temperature and rinsed again with water (4 times). The osmium-thiocarbohydrazide staining step was repeated thrice and finally samples were washed with water (6 times) and dehydrated in ethanol using graded concentrations (30%, 70% and 95%). Further, samples were dried overnight at room temperature and visualized in Thermo Axia variable pressure SEM.

### Multiplexed cytokine analysis

To measure the cytokine release profiles over time in devices, the LEGENDplex Human Anti-Virus Response Panel (13-plex) (BioLegend, catalogue number 740390) was used. Specifically, media was sampled with replacement at each timepoint and stored at −20 °C until analysis. For samples from the ALI epithelial layer of the device, 10 μl of 1× PBS was added to the epithelial chamber, allowed to incubate for 10 min, and then removed and diluted 1:4 with additional 1× PBS. All media samples were centrifuged at 500 × *g* for 5 min before freezing to remove any cells or debris and samples from each device were analysed in duplicate. The LEGENDplex kit was then run using a 3-laser Beckman Coulter CytoFLEX flow cytometer and analysed with the LEGENDplex analysis software Qognit.

### Collecting cells from devices

To perform single cell analysis on cells from the LOC devices, the cells had to first be removed from the device. This was done by removing all media from the device and flushing the media channels with 1× PBS. All channels were then filled with 0.25% trypsin and allowed to incubate for at least 30 min at 37 °C. The central vascular channel and airway channel were pump-mixed to break up all gel and remove cells from the membrane. The trypsin solution was then removed and collected, and the device was flushed with an equal volume of media to collect all residual cells. The cell suspension was then centrifuged for 5 min at 300 × *g* to pellet the cells, which were then used for downstream analysis.

### Flow cytometric analysis

Once cells were collected from the LOC devices, as described above, they were rinsed 3 times with 1× PBS to remove any residual cell culture media. They were then pelleted via centrifugation (300 × *g*, 5 min) and resuspended in a Zombie live/dead stain (BioLegend) at a 1:500 dilution in 1× PBS. The cells were allowed to incubate for 15 min at room temperature, protected from light. After incubation, 2 μl of FC block was added to each sample and allowed to incubate for an additional 15 min on ice. The relevant surface-staining antibodies were then added to each sample, and they were incubated at 4 °C, protected from light, for 30 min. For panels that included more than 2 BD Horizon Brilliant dyes, BD Horizon Brilliant Stain Buffer Plus was added to the staining solution at 10 μl per sample. The samples were then centrifuged at 300 × *g* for 5 min and resuspended in 250 μl of FACS buffer. If intracellular staining was to be done, the samples were then pelleted (300 × *g*, 5 min) and resuspended in 250 μl of BD Cytofix/Cytoperm (BD Biosciences) fixation and permeabilization buffer and incubated for 20 min at room temperature, protected from light. After incubation, the samples were pelleted (300 × *g*, 5 min) and resuspended in 100 μl of BD Perm/Wash buffer. The relevant intracellular staining antibodies were added to the samples and allowed to incubate for 30 min on ice, protected from light. The samples were then washed 3× with Perm/Wash buffer and centrifuged for a final time (300 × *g*, 5 min) and resuspended in 250 μl Perm/Wash buffer before being analysed on either a 3-laser Beckman Coulter CytoFLEX or a 5-laser Cytek Aurora. All antibodies were used at a 1:200 dilution and are listed in Supplementary Information.

### scRNA-seq

Cells were first removed from the devices as described for flow cytometric analysis. For scRNA-seq, ten devices from each group were pooled together. Library preparation was carried out using the 10x Genomics Single Cell 3′ v3 kit according to the manufacturer’s protocols. Before sequencing, the quality and integrity of the libraries were assessed using the Agilent Bioanalyzer system to ensure optimal sample input. The validated libraries were then sequenced on a NovaSeq 6000 platform, utilizing the S4 kit to generate the sequencing data. For data preprocessing, we converted raw BCL files into demultiplexed FASTQ files using the bcl2fastq software, while applying 10x Genomics-specific parameters appropriate for single-index 3′ gene expression libraries. Following the generation of FASTQ files, the Cell Ranger software (version 7.0.1) was used to perform read alignment, barcode counting and UMI counting. Specifically, the reads were aligned to the human reference genome GRCh38-2020-A. The downstream data analysis was done using SEURAT pipeline. Specifically, Seurat V4^48^, DESeq2^77^ and CellChat^49^ packages were used for analysis and graph output. Nebulosa^78^ and EnhancedVolcano^79^ packages were used to generate additional plots.

### Statistical analysis

All of the results reported are from at least two independent experiments. Investigators were blinded when possible during image analysis and cytokine response assays, and randomization of immune cell incorporation and infection was performed for all LOC studies. Data are generally presented as mean ± s.d., with figure legends indicating specific statistical tests, including two-tailed unpaired Student’s *t*-test or one-way or two-way analysis of variance (ANOVA), with indicated multiple comparisons tests. Individual data points and distributions are included in plots and data distribution was assumed to be normal, but this was not formally tested. Plotting and statistical analysis were performed using R (version 4.3.0) or GraphPad Prism (version 9).

### Reporting summary

Further information on research design is available in the Nature Portfolio Reporting Summary linked to this article.

## Extended Data

## Supplementary Material

Supp

Video

**Supplementary information** The online version contains supplementary material available at https://doi.org/10.1038/s41551-025-01491-9.

## Data Availability

The raw RNA-seq data are available in the GEO database under accession codes GSE294626 (ref. 80). The remaining data are available within the article and its Supplementary Information. Owing to the large file sizes and volume of imaging and flow cytometry data, the corresponding raw data supporting the findings of this study are available from the senior authors upon request. Source data are provided with this paper.
