# Supplementary material for: An immune-competent lung-on-a-chip for modelling the human severe influenza infection response
Source: Nat Biomed Eng. 2025 Sep 23;10(5):897–919. doi: 10.1038/s41551-025-01491-9 (PMC12969995; doi:10.1038/s41551-025-01491-9)
Supplement: Supplementary file 1 — Supplementary Figs. 1–18. [file 41551_2025_1491_MOESM1_ESM.pdf]

nature biomedical engineering

Supplementary information https://doi.org/10.1038/s41551-025-01491-9

An immune-competent lung-on-a-chip for modelling the human severe influenza infection response

In the format provided by the authors and unedited

Supplemental Figure S1

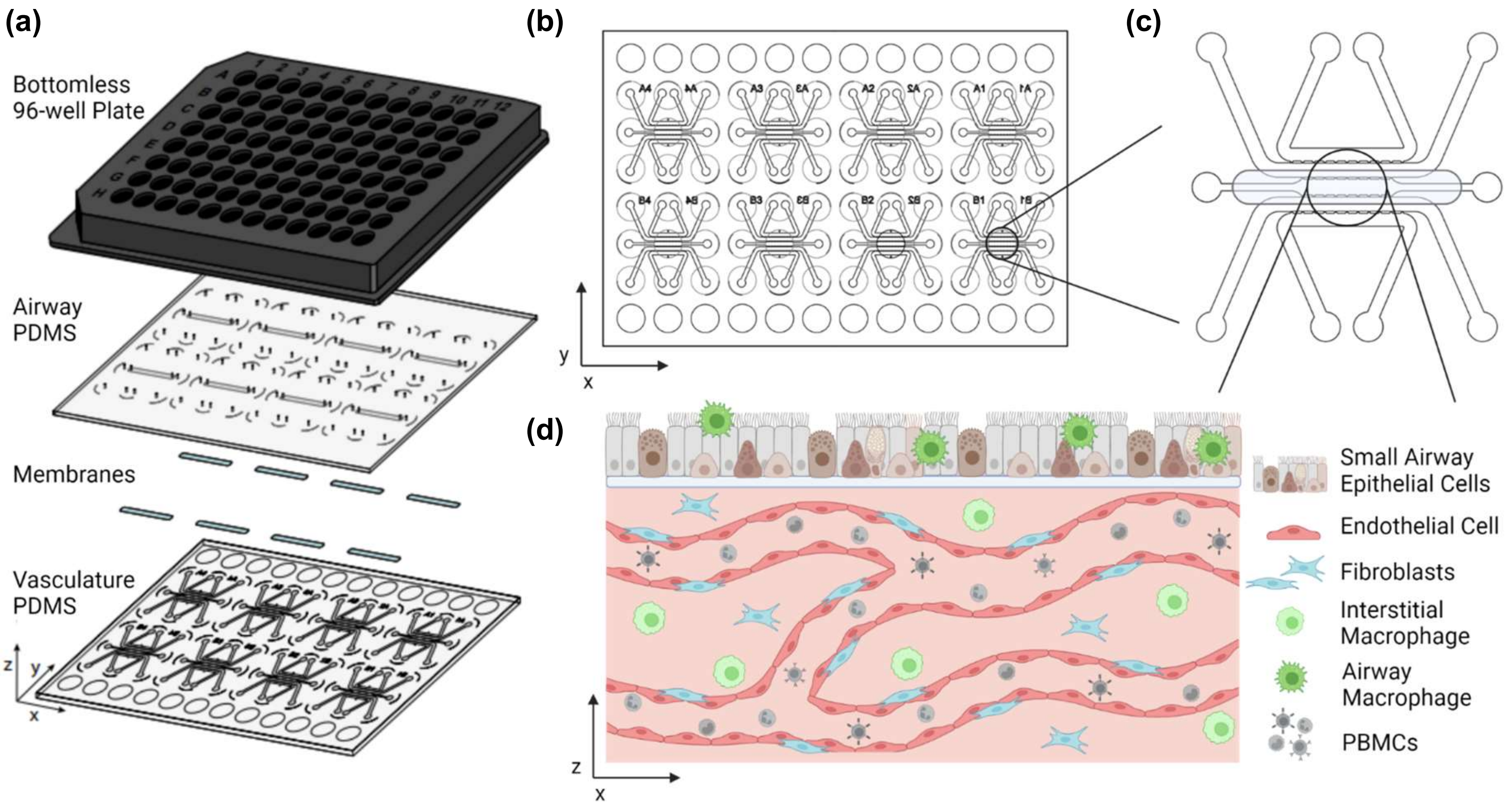

Supplemental Fig S1: (a) An exploded schematic of the 96-well plate format lung-on-chip device, with the bottom 5 channel vascular layer, PETE membranes, top single-channel airway layer, and bottomless 96 well plate comprising the media reservoirs. **(b)** A top-down view of the fully fabricated plate of lung-on-chip devices with **(c)** a top-down view of a single device. **(d)** A schematic of the structure of the central vascular and airway channel of the lung chip.

Supplemental Figure S2

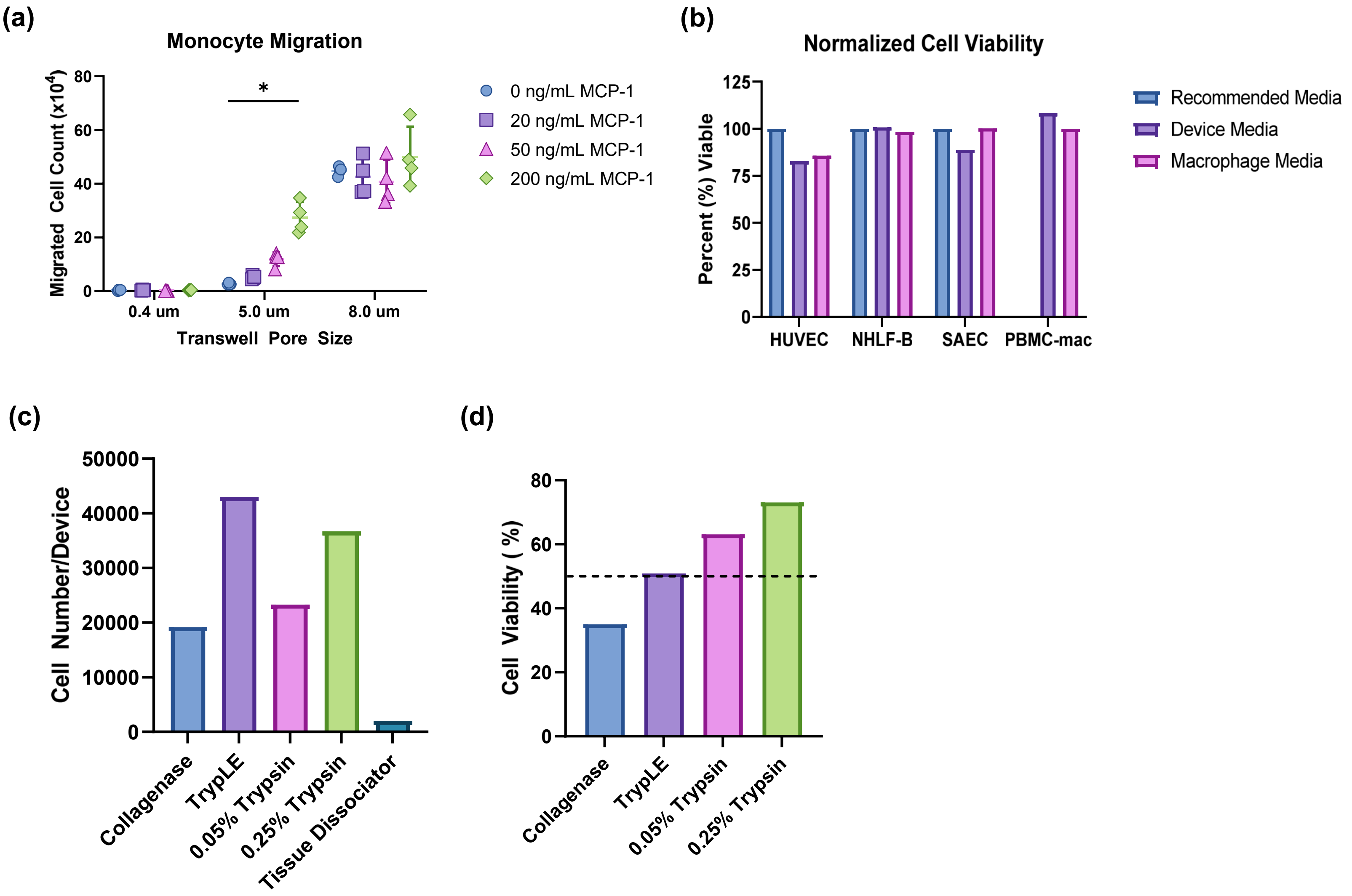

Supplemental Fig S2: **(a)** Optimization of membrane pore size. Monocyte migration across PETE transwells with pore sizes of 0.4 um, 5.0 um, and 8.0 um in response to basolateral MCP-1 concentration. Concentration-dependent migration was only observed in the 5.0um pore size membrane, thus this membrane was chosen for incorporation into the LOC. * denotes $p<0.05$ and indicates significant difference between each condition within the 5.0 um pore size group, as determined via ordinary one-way ANOVA. **(b)** Media optimization for the incorporation of tissue-resident macrophages into the interstitium of the LOC. Viability of each cell type was evaluated in the recommended media for each cell type, the device media (50:50 mix of EGM and PneumaCult-ALI, supplemented with VEGF and Ang-1), and macrophage media (RPMI + 50 ng/mL M-CSF). We found that the device media was able to sustain the culture of PBMC-derived macrophages and thus used this media formulation. **(c)** Optimization of the protocol for harvesting cells from devices in order to maximize both cell number and **(d)** cell viability. Error bars showing S.E.M..

Supplemental Figure S3

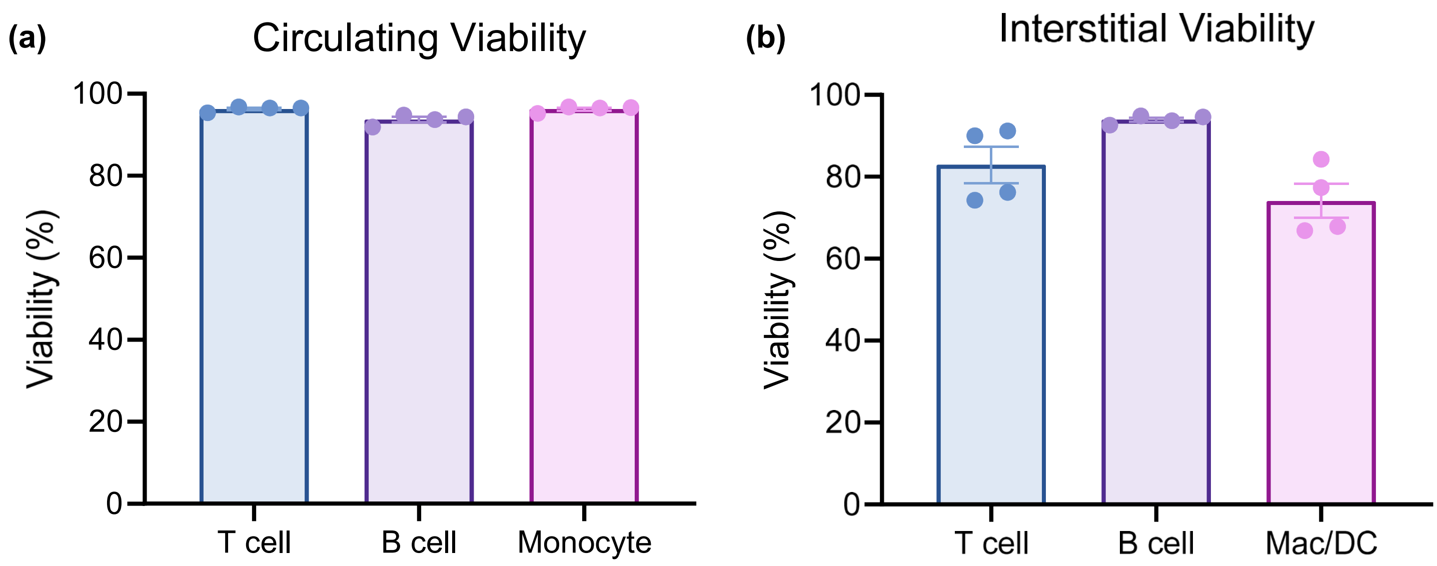

Supplemental Fig S3: (a) Viability of immune cell subsets within the circulating immune population in the IC-LOC. Viability determined through flow cytometry analysis of Zombie Aqua live/dead stain, with T cells identified as CD3+, B cells identified at CD20+, and monocytes identified as CD14+. **(b)** Viability of interstitial immune cell subsets within the IC-LOC as determined via flow cytometric analysis of Zombie Aqua live/dead stain. T and B cells were identified as defined in (A) and the Mac/DC population was identified as CD68+. Error bars showing S.E.M. N = 4 independent devices using the same immune cell donor.

Supplemental Figure S4

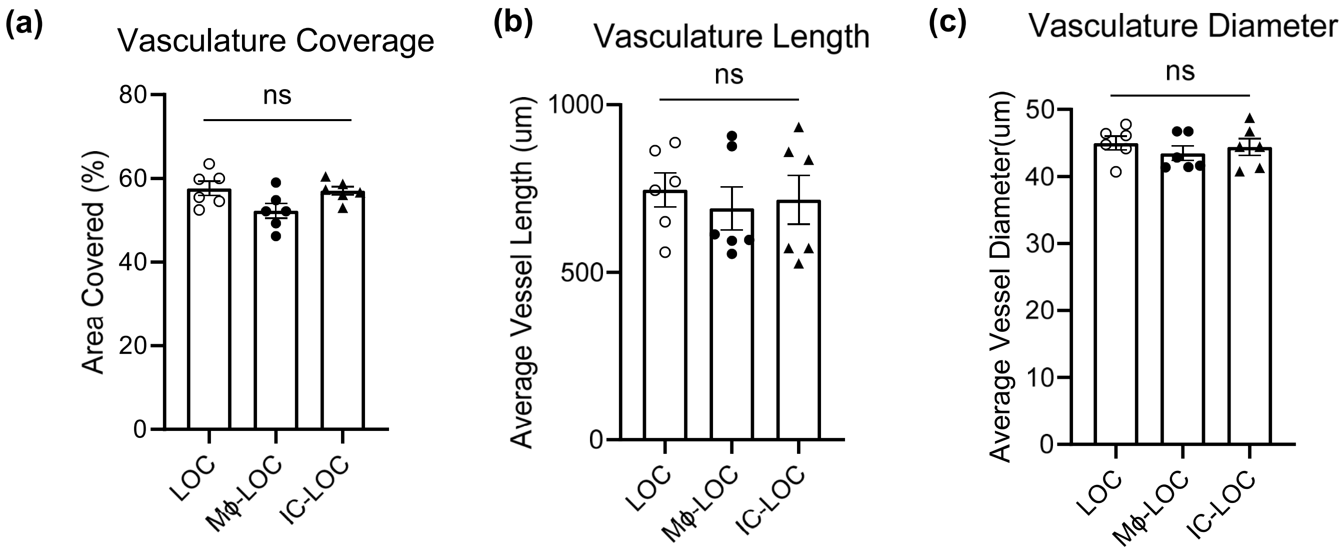

Supplemental Fig S4: Comparison of the **(a)** percent area covered by the vascular network, **(b)** average vessel length, and **(c)** average vessel diameter in LOC, Mϕ-LOC, and IC-LOC devices. N=5 independent devices from the same immune cell donor across at least 2 independent experiments and statistical significance determined via ordinary one-way ANOVA. Error bars showing S.E.M..

Supplemental Figure S5

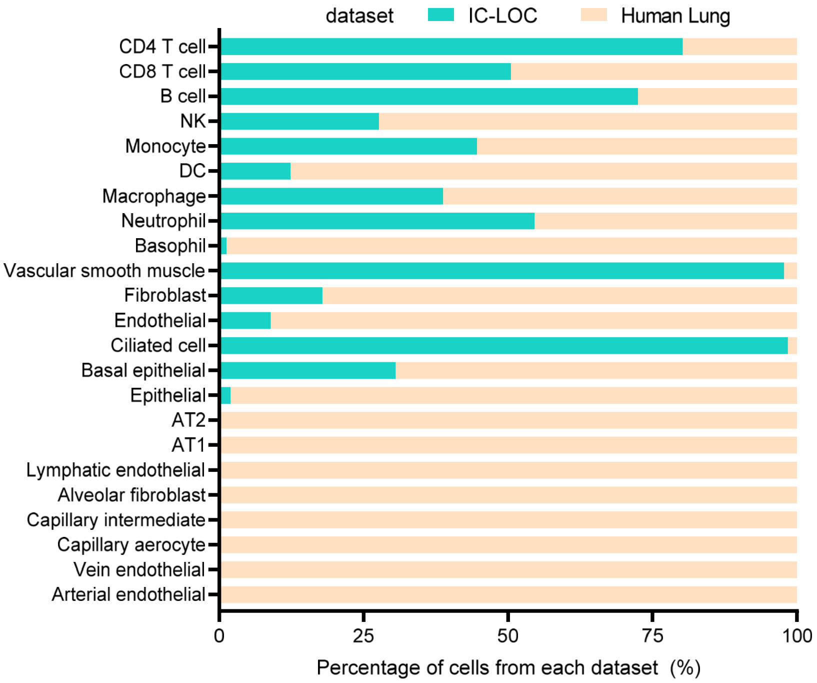

Supplemental Fig S5: Relative proportions of major cell types detected via scRNA-sequencing within the IC-LOC compared to the human lung. Single cell RNA sequencing datasets integrated using Harmony to account for batch effects. Cluster proportions shown, with teal representing IC-LOC dataset and tan representing human lung dataset.

Supplemental Figure S6

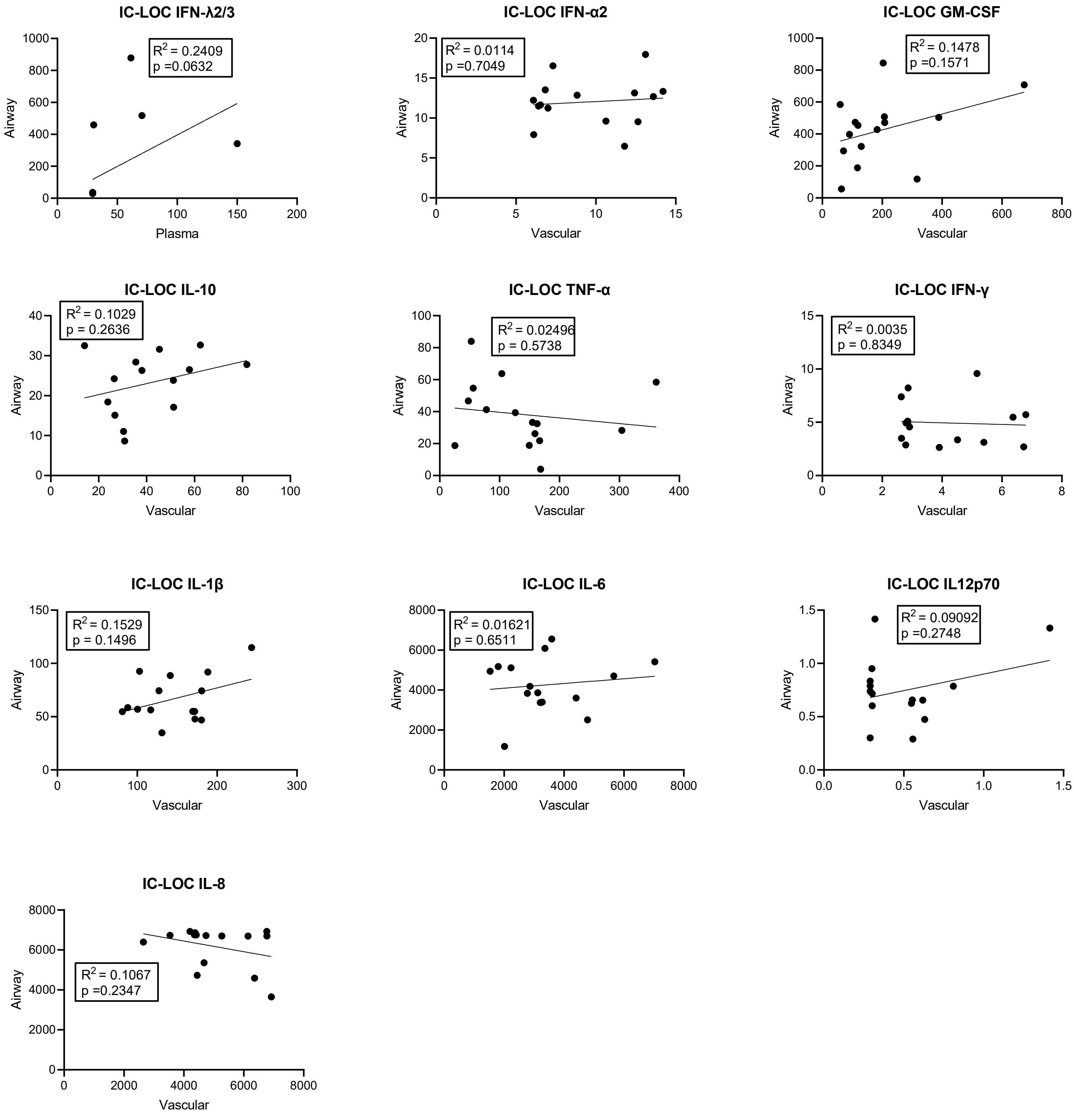

Supplemental Fig S6: Correlations between cytokine levels in the airway and vascular (interstitial) layers of the IC-LOC devices infected with H1N1. Cytokines measured with the LEGENDplex 13-plex Human Antivirus Response Panel.

Supplemental Figure S7

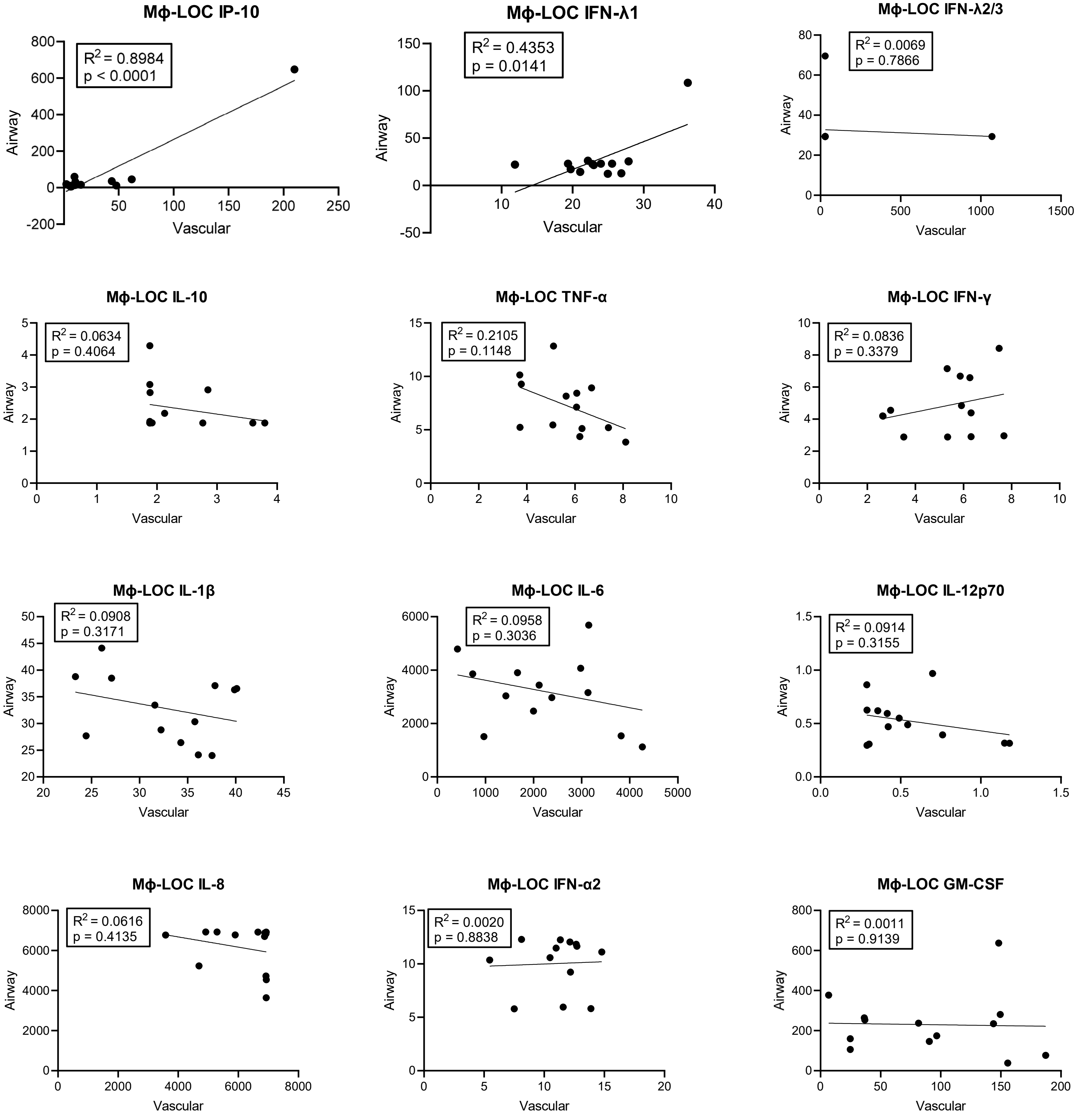

Supplemental Fig S7: Correlations between cytokines levels in the airway and vascular (interstitial) layers of the Mϕ-LOC devices infected with H1N1. Cytokines measured with the LEGENDplex 13-plex Human Antivirus Response Panel.

Supplemental Figure S8

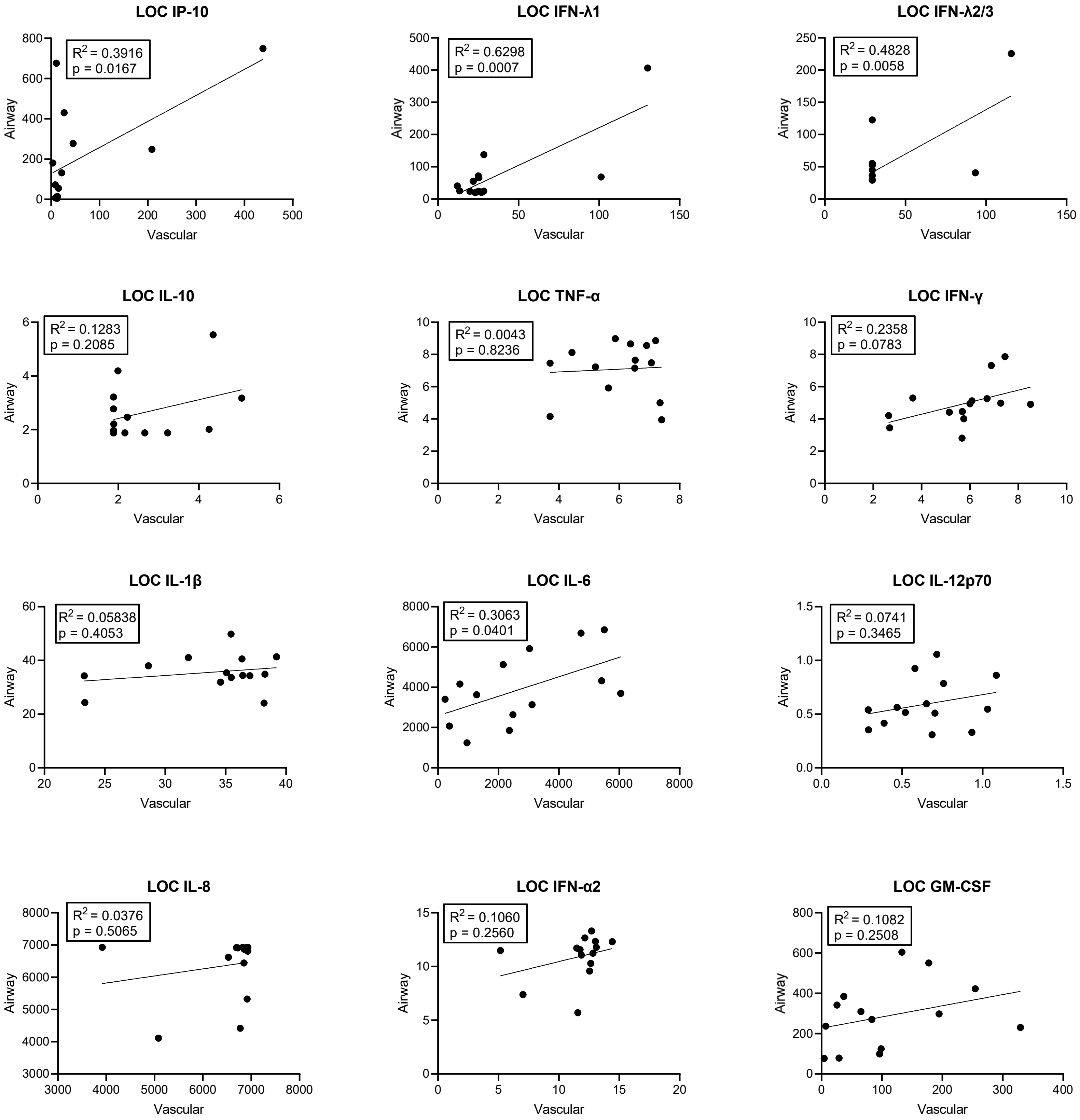

Supplemental Fig S8: Correlations between cytokines levels in the airway and vascular (interstitial) layers of the LOC devices infected with H1N1. Cytokines measured with the LEGENDplex 13-plex Human Antivirus Response Panel.

Supplemental Figure S9

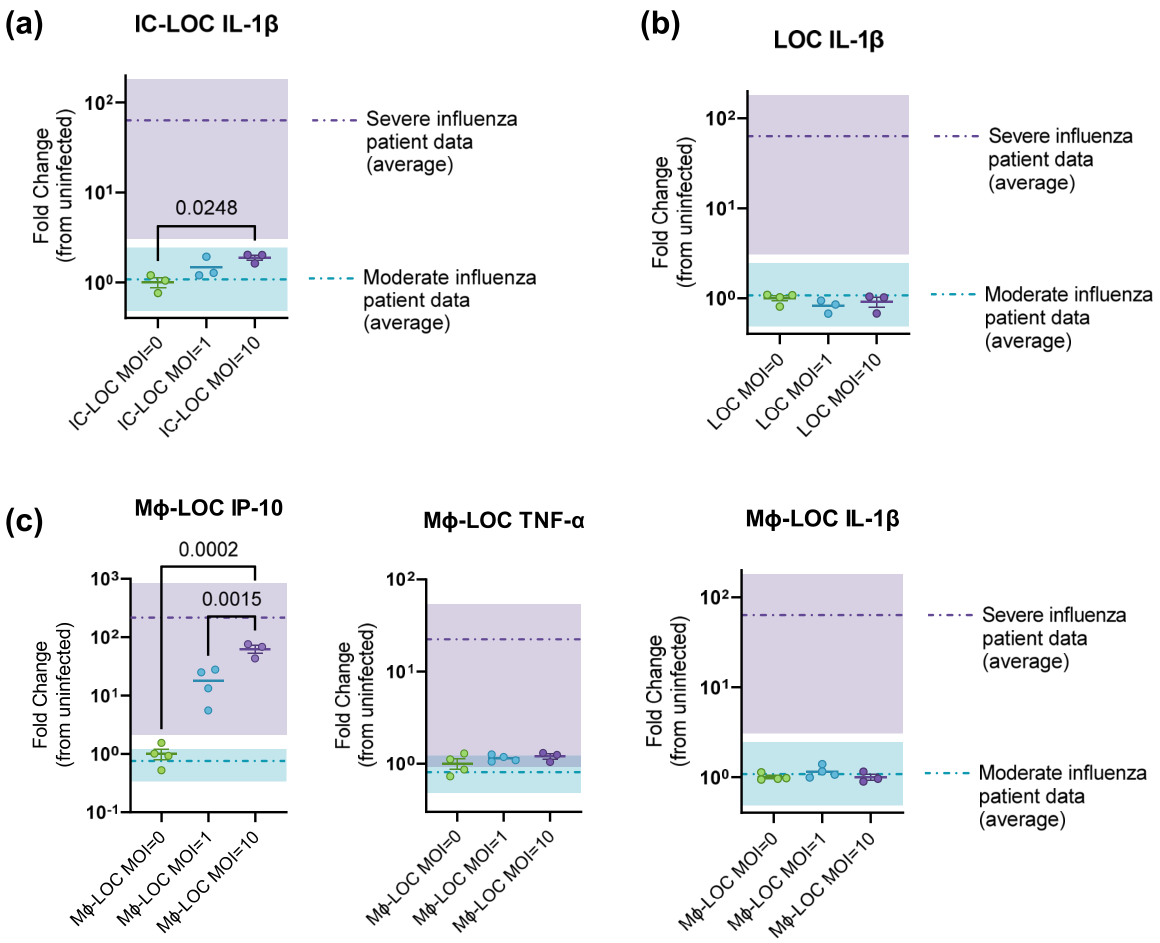

Supplemental Fig S9: Fold change in IL-1β in the airway of **(a)** IC-LOC and **(b)** LOC devices at MOI 1 and MOI 10 at 72 hpi, relative to uninfected devices (MOI 0). **(b)** Fold change in IP-10, TNF-α, and IL-1β in the airway of MΦ-LOC devices at 72 hpi at MOI 1 and MOI 10, relative to uninfected devices (MOI 0). For all plots, changes in cytokine levels are compared to patient data from moderate and severe influenza patients, with the range in patient data shown by the solid color band and average in patient data shown by the dotted line. Purple corresponds to severe influenza patient data, while teal corresponds to moderate influenza patient data. Statistical significance determined via ordinary one-way ANOVA with Tukey's multiple comparison test. N=3-4 independent devices from the same immune cell donor across at least 2 independent experiments.

Supplemental Figure S10

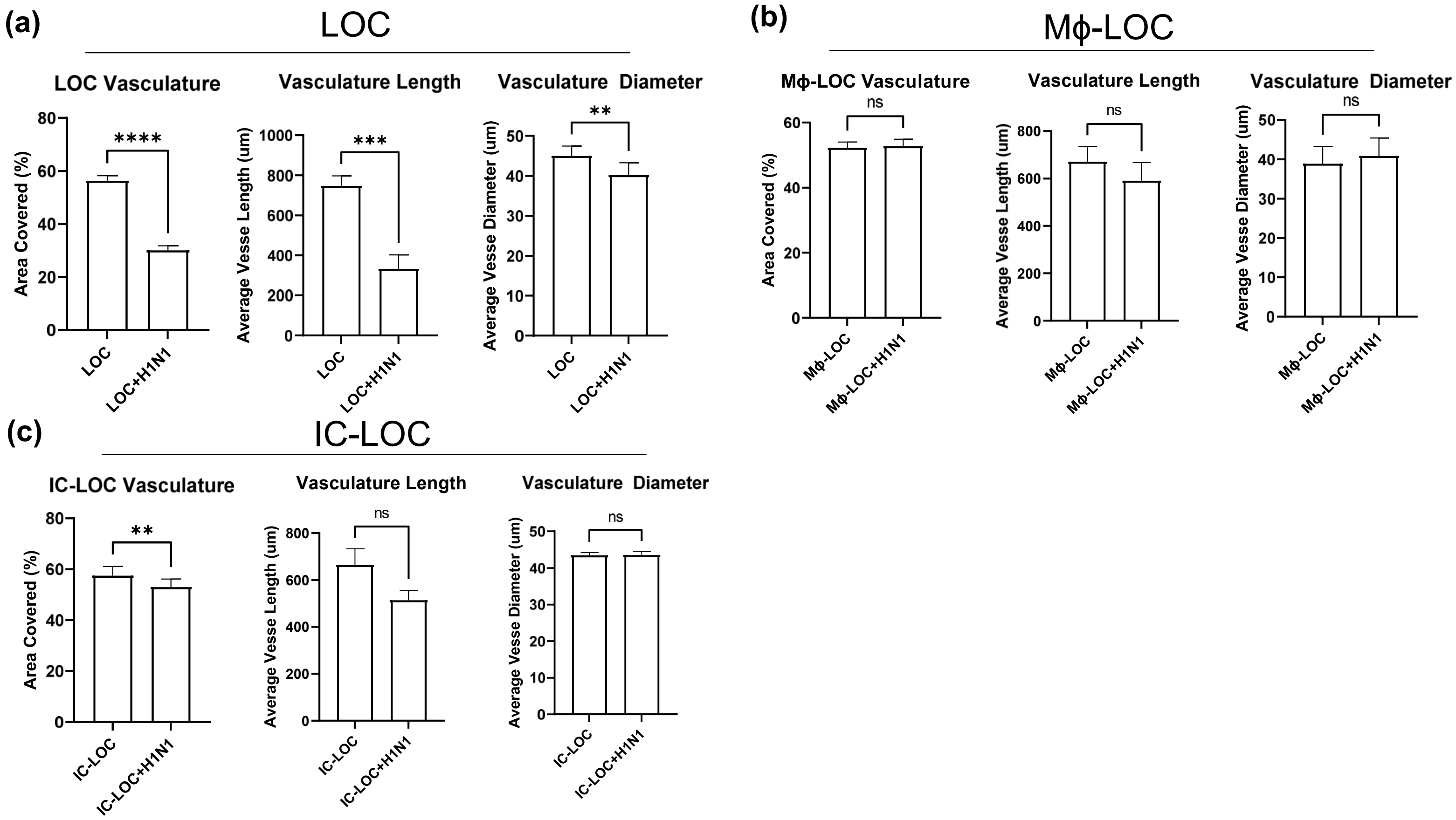

Supplemental Figure S10: (A) Quantification of the effect of H1N1 infection on the percent area covered by the vascular network, the average vessel length, and the average vessel diameter in LOC, **(B)** Mϕ-LOC, and **(C)** IC-LOC devices. N = 7 independent devices from the same immune cell donor across at least 2 independent experiments. Statistical significance determined via two-tailed unpaired t-test, with * $p<0.05$, ** $p<0.01$, *** $p<0.001$, **** $p<0.0001$. Error bars show S.E.M..

Supplemental Figure S11

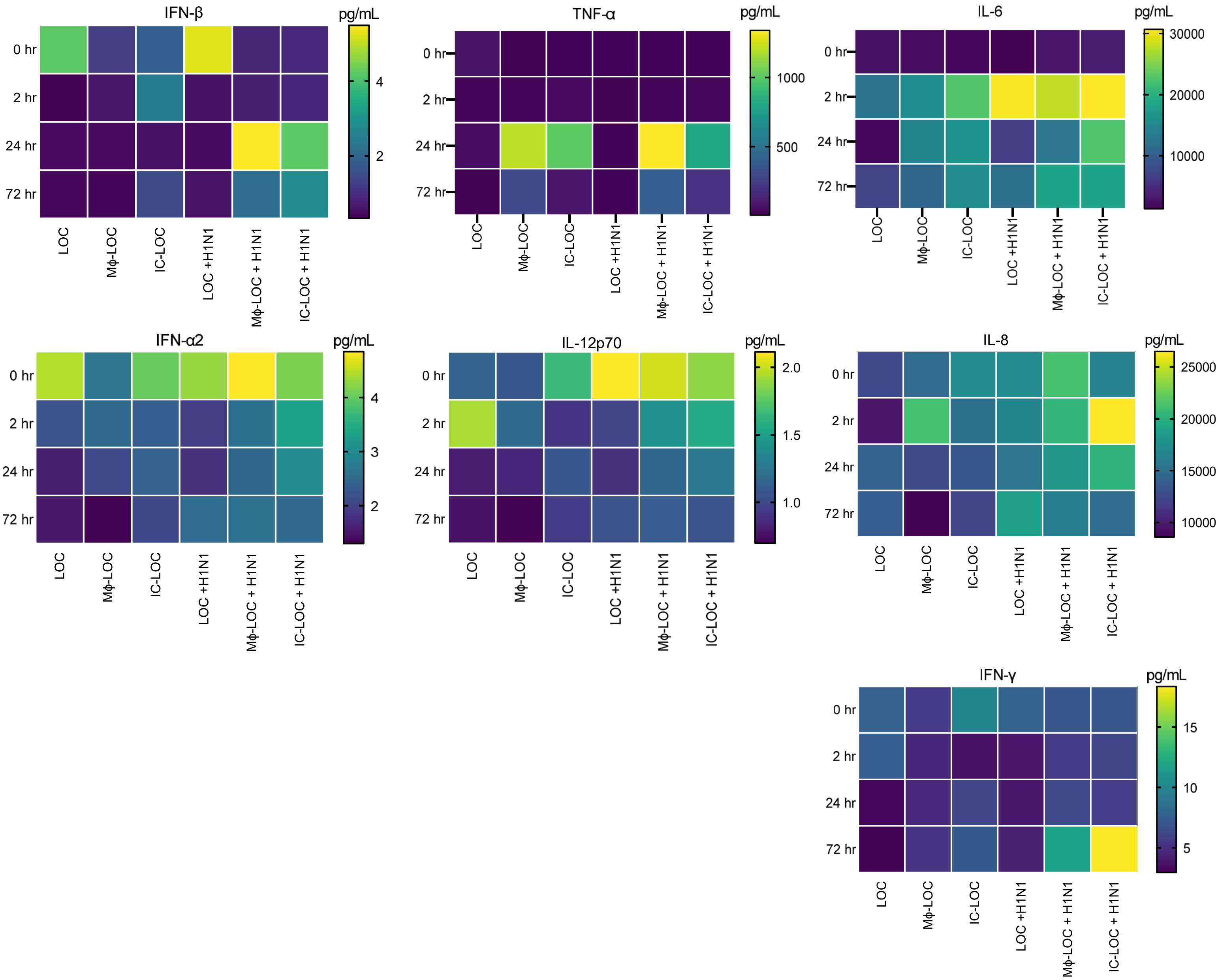

Supplemental Figure S11: Cytokine release profiles measured in the airway layer of the device using the LEGENDplex 13-plex Human Antivirus Response Panel. Heatmaps colored according to expression levels in pg/mL, with significance indicated numerically. Significance compared to time 0 determined via ordinary one-way ANOVA and displayed on plots.

Supplemental Figure S12

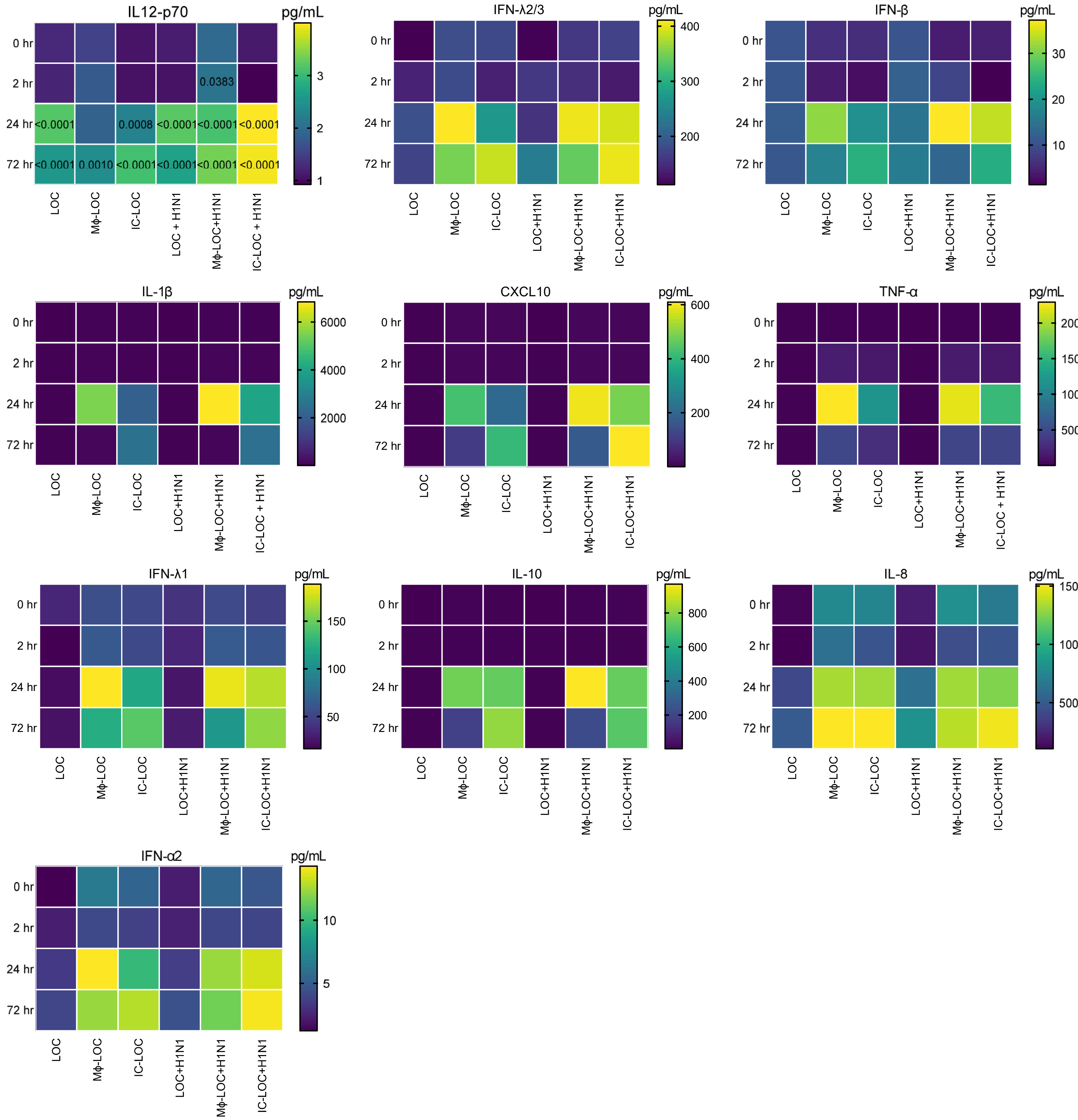

Supplemental Figure S12: Cytokine release profiles measured in the interstitial layer of the device using the LEGENDplex 13-plex Human Antivirus Response Panel. GM-CSF was over the detection limit and thus is not shown. Heatmaps colored according to expression levels in pg/mL, with significance indicated numerically. Significance compared to time 0 determined via ordinary one-way ANOVA and displayed on plots.

Supplemental Figure S13

(a)

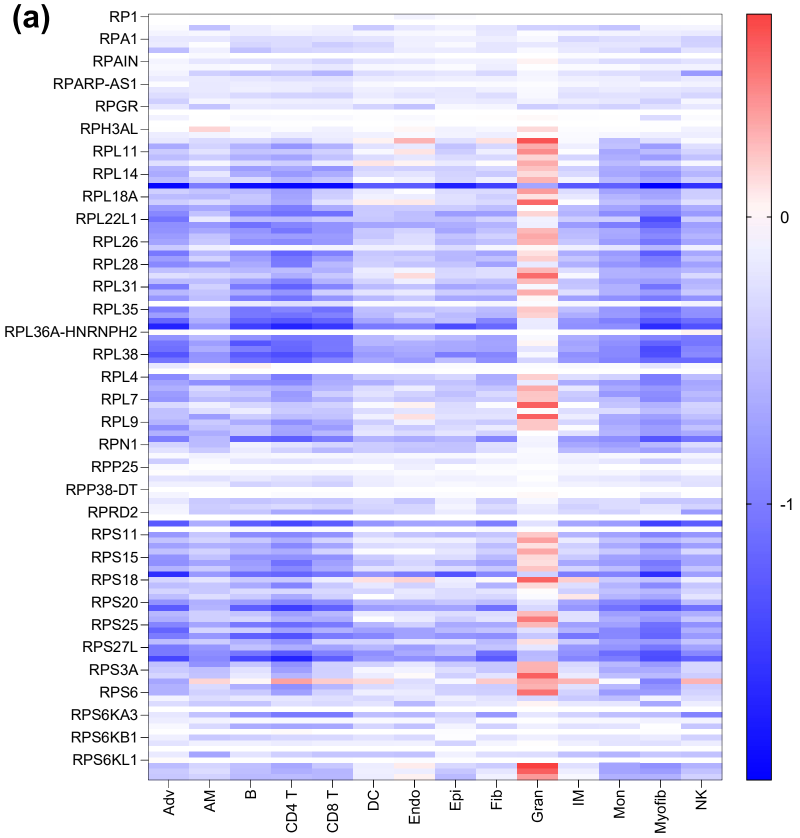

(b)

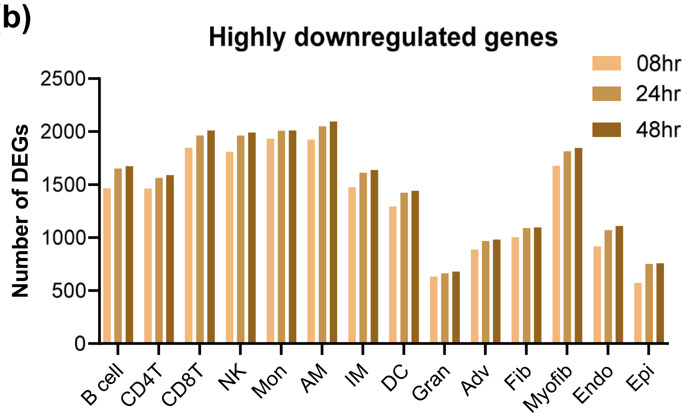

Supplementary Figure S13: (a) Gene set enrichment analysis (GSEA) analysis of 50 ribosomal genes, showing significant downregulation across the majority of cell types within the IC-LOC in response to infection (48 hpi), indicative of major transcriptional shutdown. **(b)** Differentially expressed gene (DEG analysis showing the number of downregulated genes in each cell type in the H1N1 infected devices (08hr, 24hr, and 48hr) relative to the uninfected control.

Supplemental Figure S14

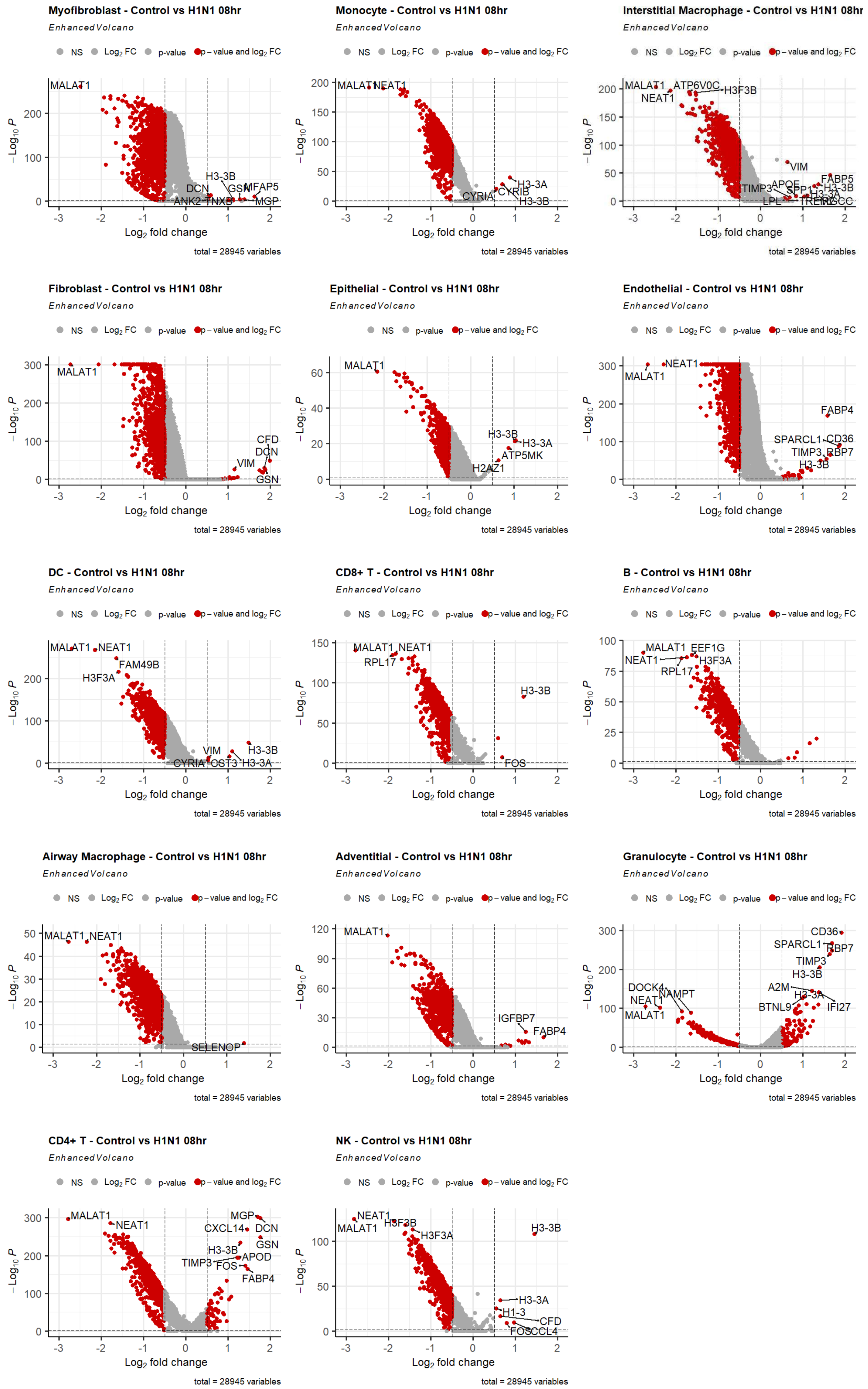

Supplemental Figure S14: Volcano plots showing up and downregulated genes in each cell type cluster at the 8 hour infection timepoint compared to the uninfected control.

Supplemental Figure S15

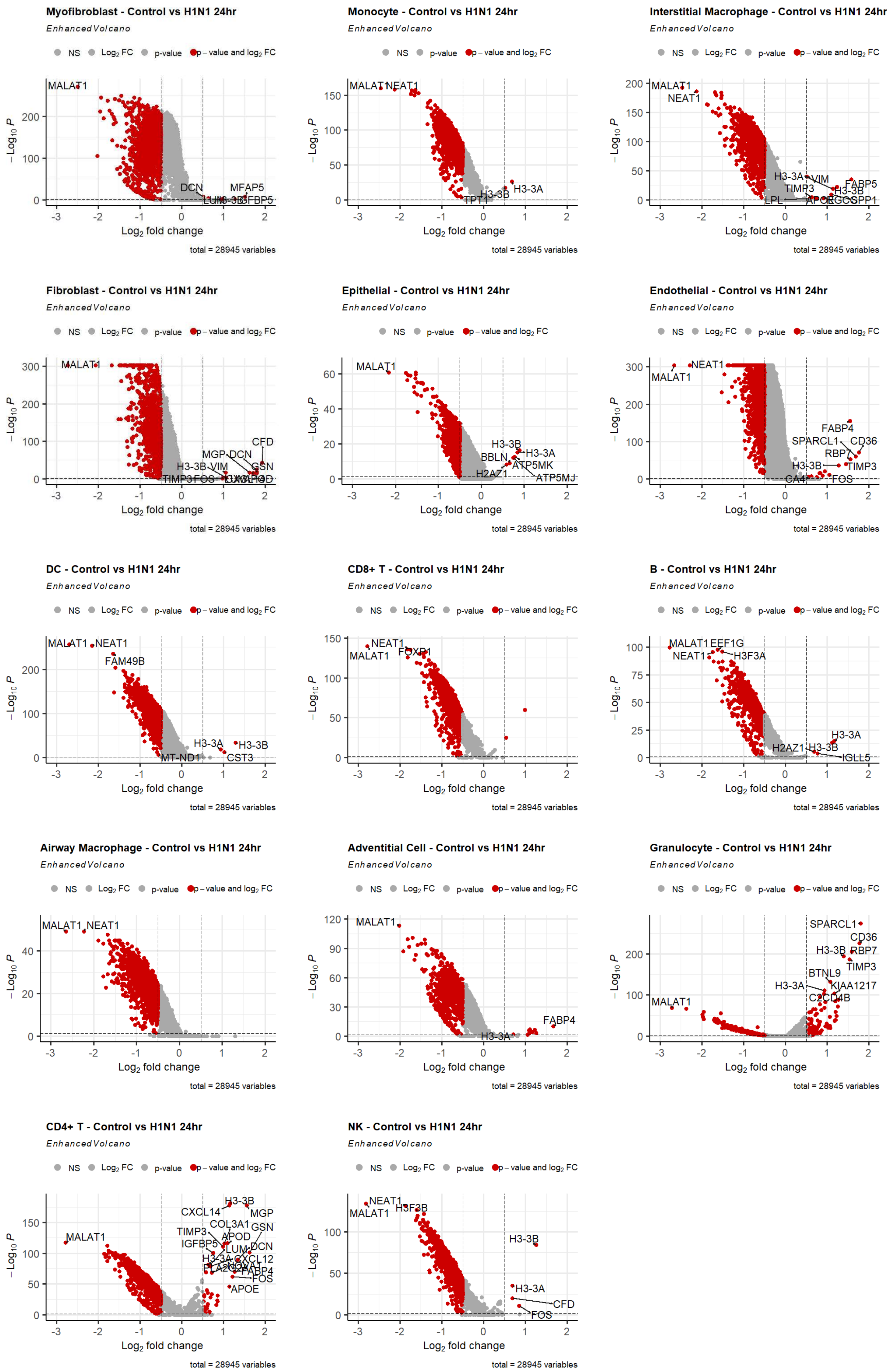

Supplemental Figure S15: Volcano plots showing up and downregulated genes in each cell type cluster at the 24 hour infection timepoint compared to the uninfected control.

Supplemental Figure S16

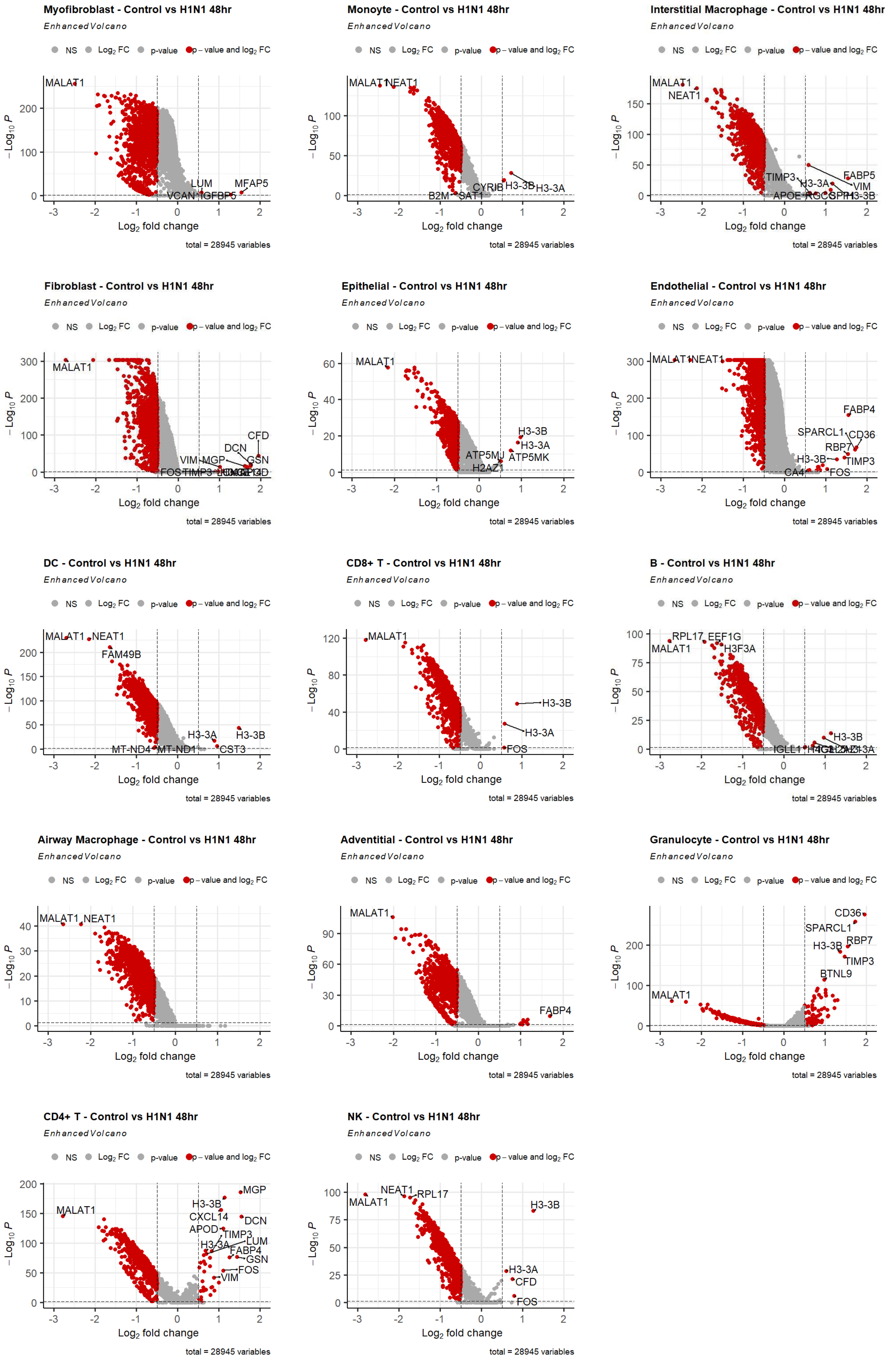

Supplemental Figure S16: Volcano plots showing up and downregulated genes in each cell type cluster at the 48 hour infection timepoint compared to the uninfected control.

Supplemental Figure S17

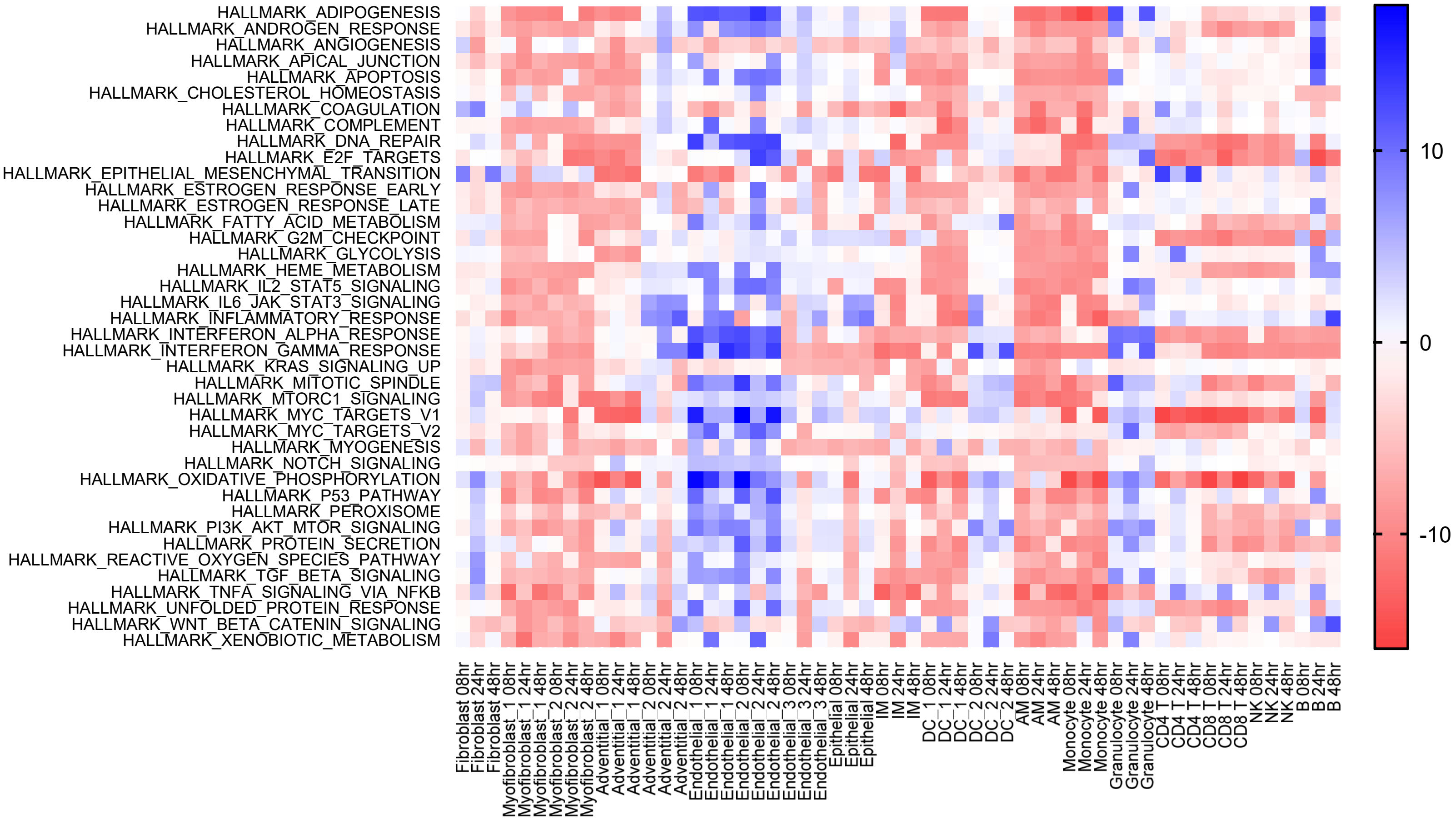

Supplementary Figure S17: *DGE* analysis of hallmark pathways in the infected (08, 24, 48 hr) IC-LOC compared to the uninfected control.

Supplemental Figure S18

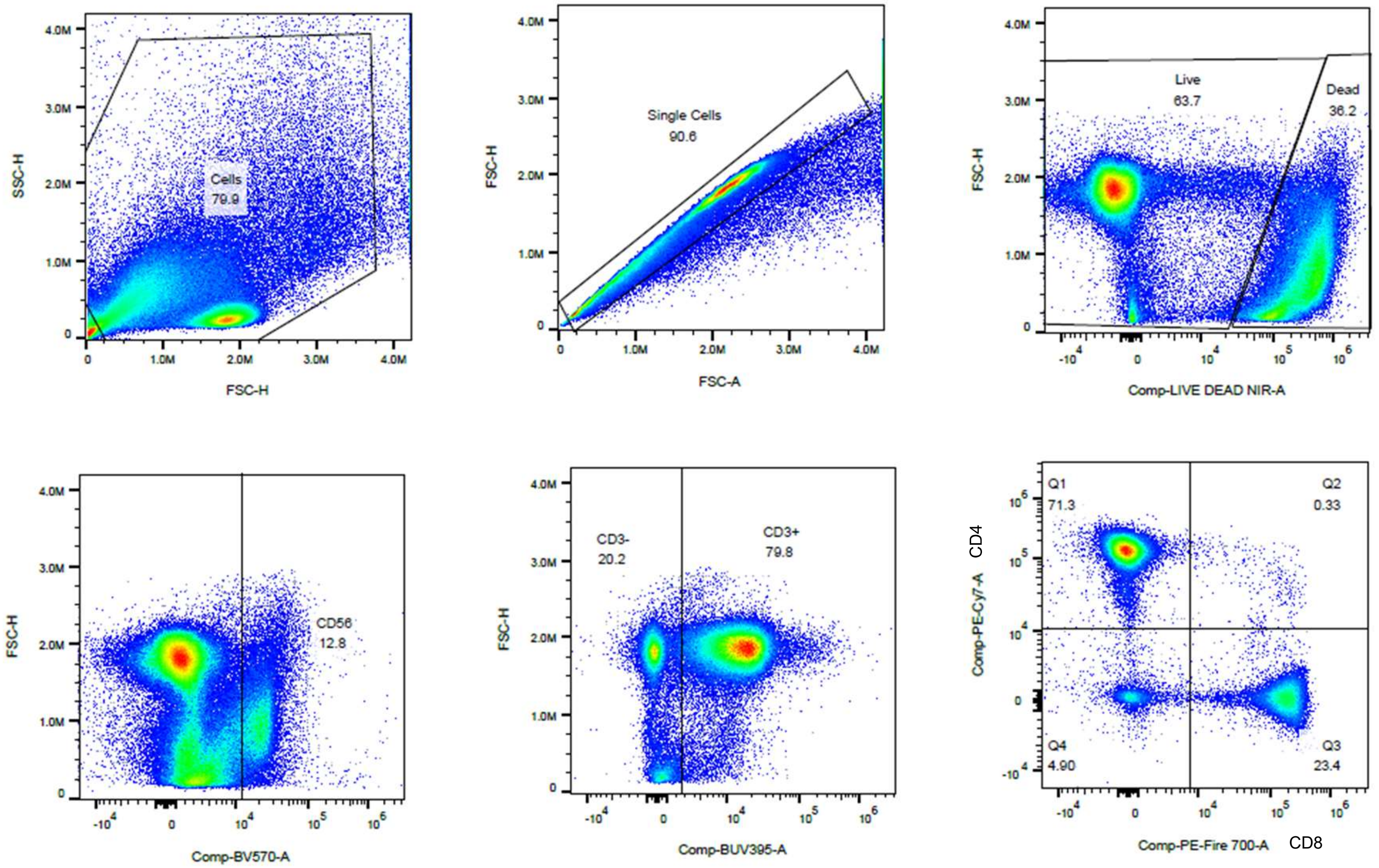

Supplementary Figure S18: Gating strategy used to define the NK cell (CD56+), CD4 T cell (CD3+ CD4+) and CD8 T cell (CD3+ CD8+) populations for analysis of activation markers.

Supplementary Table 1: List of antibodies used for both fluorescence microscopy and flow cytometry.

	**Antibody Name**	**Vendor**	**Catalog Number**
Microscopy Antibodies	Anti-Hu CD206 (19.2), Alexa Fluor 488	Invitrogen	53-2069-42
	Anti-Hu CC10 (E-11), Alexa Fluor 488	Santa Cruz Biotechnology	sc-365992 AF488
	Anti-Hu CC10 (E-11), FITC	Santa Cruz Biotechnology	sc-365992 FITC
	Anti-Hu CD326 (9C4), FITC (Ep-CAM)	BioLegend	324203
	Anti-B-actin (2F1-1), Alexa Fluor 488	BioLegend	643812
	Anti-Hu Integrin a11 (2F1C10), Alexa Fluor 488	Santa Cruz Biotechnology	sc-390091 AF488
	Anti-Hu Cytokeratin Pan Monoclonal Antibody (C-11), Alexa Fluor 488	Invitrogen	MA5-18156
	Anti-Hu CD3 (UCHT1), Alexa Fluor 488	BioLegend	300454
	Anti-Hu CD20 (2H7), Alexa Fluor 488	BioLegend	302316
	Anti-Hu CD152 (CTLA-4) (14D3), PE	Invitrogen	12-1529-42
	Anti-Hu CD279 (PD-1) (MIH4), APC	Invitrogen	17-9969-42
	Anti-Hu Elastin (BA-4), Alexa Fluor 546	Santa Cruz Biotechnology	sc-58756 AF546
	Anti-Hu CD31 (WM59), Alexa Fluor 594	BioLegend	303126
	Anti-Hu CD14 (HCD14), Alexa Fluor 594	BioLegend	325630
	Anti-Hu CD4 (RPA-T4), Alexa Fluor 594	BioLegend	300544
	Anti-Hu SP-C (H-8), Alexa Fluor 488	Santa Cruz Biotechnology	sc-518029 AF488
	Anti-Hu Z0-1 (ZO1-1A12) Monoclonal Antibody, Alexa Fluor 488	Invitrogen	MA3-39100-A488
	Anti-Hu Beta Tubulin Monoclonal Antibody (2 28 33), Alexa Fluor 647	Invitrogen	MA3-22600-A647
	Anti-Hu Mucin 5AC (EPR16904), Alexa Fluor 555	Abcam	ab218714
	Anti-Hu Mucin 5AC (45M1), Alexa Fluor 647	Santa Cruz Biotechnology	sc-21701 AF647
	Anti-Hu Z0-1 Monoclonal Antibody (ZO1-1A12), Alexa Fluor 555	Invitrogen	MA3-39100-A555
	Anti-Hu CD68 (KP1), Alexa Fluor 488	Abcam	ab222914
	Anti-Hu CD68 (Y1/82A), Alexa Fluor 647	BioLegend	333820
	Anti-Hu CD140a (PDGFRa) (16A1), APC	BioLegend	323512
	Anti-Hu CD80 (MEM233), Alexa Fluor 647	Invitrogen	A51018
	Anti-B-actin (2F1-1), Alexa Fluor 647	BioLegend	643810
	Anti-Hu CD45 (HI30), Alexa Fluor 647	BioLegend	304056

	Anti-Hu Laminin alpha 1, Alexa Fluor 647	R&D systems	IC4187R-100UG
	Anti-Hu CD56 (NCAM) (5.1H11), Alexa Fluor 647	BioLegend	362514
	Anti-Hu CD8a (C8/114B), Alexa Fluor 647	BioLegend	372906
	Anti-Hu S100A4 (NJ-4F3-D1), APC	BioLegend	370005
	Anti-Hu Z0-1 Monoclonal Antibody (ZO1-1A12), Alexa Fluor 647	Invitrogen	MA3-39100-A647
	Lectin-LEA, Dylight	Invitrogen	2384029
Flow Antibodies	Anti-Hu CD14 (S18004B), Spark UV 387	BioLegend	399215
	Anti-Hu CD11b (ICRF44), Brilliant UltraViolet 805	Invitrogen	368-0118-42
	Anti-Hu CD11c (Bu15), APC/Fire 750	BioLegend	337240
	Anti-Hu CD206 (15-2), Pacific Blue	BioLegend	321152
	Anti-Hu CD1c (L161), APC	BioLegend	331524
	Anti-Hu CD141 (M80), PE	BioLegend	344104
	Anti-Hu CD103 (Ber-ACT8), Brilliant Ultraviolet 605	BioLegend	350218
	Anti-Hu MARCO (PLK-1), PE-Cyanine7	Invitrogen	25-5447-42
	Anti-Hu CD64 (10.1), Brilliant UltraViolet 510	BioLegend	305028
	Anti-Hu CD303 (201A), PerCP cyanine5.5	BioLegend	354210
	Anti-Hu CCR7 (3D12), Brilliant UltraViolet 661	BD Biosciences	376-1979-42
	Anti-Hu CD14 (S18004B), Spark UV 387	BioLegend	399215
	Anti-Hu CD14 (M5E2), PE	BioLegend	301850
	Anti-Hu CD68 (Y1/82A), Alexa Fluor 488	BioLegend	333812
	Anti-Hu CD206 (19.2), PerCP-eFluor 710	Invitrogen	46-2069-42
	Anti-Hu CD11c (3.9), APC-eFluor 780	Invitrogen	47-0116-42
	Anti-Hu CD45 (HI30), Spark UV 387	BioLegend	304086
	Anti-Hu CD20 (2H7), Brilliant UltraViolet 737	Invitrogen	367-0209-42
	Anti-Hu CD56 (NCAM) (TULY56), Super Bright 600	Invitrogen	63-0566-42
	Anti-Hu CD15 (W6D3), Brilliant Violet 711	BioLegend	323049
	Anti-Hu CD11b (ICRF44), APC	Invitrogen	17-0118-42
	Anti-Hu CD138 (MI15), FITC	BioLegend	356507
	Anti-Hu CD31 (WM59), PerCP/Cyanine5.5	BioLegend	303132
	Anti-Vimentin (O91D3), Alexa Fluor 594	BioLegend	677804

	Anti-Hu CD68 (Y1/82A), PE/Cyanine7	BioLegend	333816
	Anti-Cytokeratin (pan reactive) (C-11), Alexa Fluor 647	BioLegend	628604
	Anti-Hu CD326 (Ep-CAM) (9C4), Alexa Fluor 700	BioLegend	324244
	Anti-Hu CD8a (RPA-T8), APC-eFluor 780	Invitrogen	47-0088-42
	Anti-Hu CD86 (IT2.2), Super Bright 600	Invitrogen	63-0869-42
	Anti-Hu HLA-DR (LN3), Super Bright 780	Invitrogen	78-9956-42
	Zombie UV Fixable Viability	BioLegend	423108
	Anti-Hu CD15 (MMA), PerCP-eFluor 710	Invitrogen	46-0158-42
	Anti-Hu CD3 (OKT3), FITC	Invitrogen	11-0037-42
	Anti-Hu CD4 (RPA-T4), Super Bright 780	Invitrogen	78-0049-42
	Anti-Hu CD14 (M5E2), PE	BioLegend	301850
	Anti-Hu CD20 (2H7), APC	Invitrogen	17-0209-42
	Anti-Hu CD45 (2D1), Super Bright 780	Invitrogen	78-9459-42
	Anti-Hu CD133 (Prominin-1) (TMP4), PerCP-eFluor 710	Invitrogen	46-1338-42
	Anti-Hu CD14 (61D3), APC	Invitrogen	17-0149-42
	LIVE/DEAD Blue	Invitrogen	L23105
	Anti-Hu CD25 (BC96), Super Bright 780	Invitrogen	78-0259-42
	Anti-Hu CD69 (FN50), RB705	BD Biosciences	570278
	Anti-Hu NKp44 (p44-8), Brilliant UltraViolet 615	BD Biosciences	752353
	Anti-Hu HLA-DR (G46-6), Brilliant UltraViolet 737	BD Biosciences	568351
	Anti-Hu CD56 (HCD56), Brilliant Violet570	BioLegend	318330
	Anti-Hu CD8 (SK1), PE-Fire700	BioLegend	344766
	Anti-Hu CD16 (3G8), Brilliant Violet 650	BD Biosciences	563691
	Anti-Hu NKG2D (1D11), PE-Cyanine5	BioLegend	320844
	Anti-Hu CD4 (RPA-T4), PE-Cyanine7	BioLegend	300512
	Anti-Hu CD3 (SK7), Brilliant UltraViolet 395	BD Biosciences	565983
	Anti-Hu CD45 (HI30), Brilliant UltraViolet 496	BD Biosciences	569101
	Anti-Hu IFN-γ (B27), V500	BD Biosciences	561980
	Anti-Hu Granzyme B (QA18A28), PerCP	BioLegend	396416
