# Supplementary material for: An immune-competent lung-on-a-chip for modelling the human severe influenza infection response
Source: Nat Biomed Eng. 2025 Sep 23;10(5):897–919. doi: 10.1038/s41551-025-01491-9 (PMC12969995; doi:10.1038/s41551-025-01491-9)
Supplement: Supplementary file 2 — Reporting Summary [file 41551_2025_1491_MOESM2_ESM.pdf]

nature research

Corresponding author(s): Krishnendu Roy, Ankur Singh

Last updated by author(s): 4/20/2025

Reporting Summary

Nature Research wishes to improve the reproducibility of the work that we publish. This form provides structure for consistency and transparency in reporting. For further information on Nature Research policies, see Authors & Referees and the Editorial Policy Checklist.

Statistics

For all statistical analyses, confirm that the following items are present in the figure legend, table legend, main text, or Methods section.

n/a	Confirmed	
☐	☒	The exact sample size (*n*) for each experimental group/condition, given as a discrete number and unit of measurement
☐	☒	A statement on whether measurements were taken from distinct samples or whether the same sample was measured repeatedly
☐	☒	The statistical test(s) used AND whether they are one- or two-sided<br>*Only common tests should be described solely by name; describe more complex techniques in the Methods section.*
☒	☐	A description of all covariates tested
☐	☒	A description of any assumptions or corrections, such as tests of normality and adjustment for multiple comparisons
☐	☒	A full description of the statistical parameters including central tendency (e.g. means) or other basic estimates (e.g. regression coefficient) AND variation (e.g. standard deviation) or associated estimates of uncertainty (e.g. confidence intervals)
☐	☒	For null hypothesis testing, the test statistic (e.g. *F*, *t*, *r*) with confidence intervals, effect sizes, degrees of freedom and *P* value noted<br>*Give P values as exact values whenever suitable.*
☒	☐	For Bayesian analysis, information on the choice of priors and Markov chain Monte Carlo settings
☒	☐	For hierarchical and complex designs, identification of the appropriate level for tests and full reporting of outcomes
☐	☒	Estimates of effect sizes (e.g. Cohen's *d*, Pearson's *r*), indicating how they were calculated

Our web collection on statistics for biologists contains articles on many of the points above.

Software and code

Policy information about availability of computer code

Data collection	Volocity v6.3, SpectroFlo v3.2.0, CytExpert v 2.6, Agilent BioTek Gen5 v 3.07
Data analysis	FlowJo v 10.8; GraphPad Prism 9; ImageJ 1.54; AngioTool 0.5. For scRNA-seq, library preparation was carried out using the 10x Genomics Single Cell 3' v3 kit according to the manufacturer's protocols. Prior to sequencing, the quality and integrity of the libraries were assessed using the Agilent Bioanalyzer system to ensure optimal sample input. The validated libraries were then sequenced on a NovaSeq 6000 platform, utilizing the S4 kit to generate the sequencing data. For data preprocessing, we converted raw BCL files into demultiplexed FASTQ files using the bcl2fastq software, while applying 10x Genomics-specific parameters appropriate for single-index 3' gene expression libraries. Following the generation of FASTQ files, the Cell Ranger software (version 7.0.1) was employed to perform read alignment, barcode counting, and UMI counting. Specifically, the reads were aligned to the human reference genome GRCh38-2020-A. The downstream data analysis was done using SEURAT pipeline (v4) using R v 4.3.0.

For manuscripts utilizing custom algorithms or software that are central to the research but not yet described in published literature, software must be made available to editors/reviewers. We strongly encourage code deposition in a community repository (e.g. GitHub). See the Nature Research guidelines for submitting code & software for further information.

Data

Policy information about availability of data

All manuscripts must include a data availability statement. This statement should provide the following information, where applicable:

- Accession codes, unique identifiers, or web links for publicly available datasets
- A list of figures that have associated raw data
- A description of any restrictions on data availability

The raw sequencing data files generated in the IC-LOC scRNA sequencing experiments are available in the GEO database under accession code GSE294626. The human genome used for alignment is available under NCBI RefSeq Assembly number GCF_000001405.40. All source data underlying the findings in this study are

available in the supplementary materials. Due to the large number and size of the flow cytometry and imaging files, these raw files are available from the senior authors upon request.

Field-specific reporting

Please select the one below that is the best fit for your research. If you are not sure, read the appropriate sections before making your selection.

☒ Life sciences ☐ Behavioural & social sciences ☐ Ecological, evolutionary & environmental sciences

For a reference copy of the document with all sections, see nature.com/documents/nr-reporting-summary-flat.pdf

Life sciences study design

All studies must disclose on these points even when the disclosure is negative.

Sample size	Sample sizes were selected based on power calculations and previous experience in our lab (Mejias at al, Lab Chip 2019. Nelson et al, Biomaterials 2021. Ghoshal et al, Biomaterials 2024).
Data exclusions	Data was only excluded if devices did not meet the established QC standards for the lung chip devices or if data points were identified as outliers using Outlier Identification in GraphPad Prism.
Replication	All studies were replicated at least two times and results were verified, with the exception of scRNA-seq in which 10 devices were pooled for each sequencing sample in efforts to cover device variability while keeping cost considerations in mind.
Randomization	Immune cell incorporation was randomized among devices and influenza infection was randomized on a plate-basis (8 devices). For inhibitor studies, the addition of inhibitor or PBS was also randomized across devices.
Blinding	Investigators were blinded when possible during all data collection and analysis. Blinding was performed by assigning samples with device IDs, independent of their experimental group (LOC model, infection status, treatment), and re-annotating with experimental grouping at the end. In some cases, such as imaging of fluorescently-labeled immune cells, blinding to the model (LOC, M-LOC, IC-LOC) was not possible; however, in these cases blinding to treatment condition (+/- H1N1, +/- inhibitors) was maintained.

Reporting for specific materials, systems and methods

We require information from authors about some types of materials, experimental systems and methods used in many studies. Here, indicate whether each material, system or method listed is relevant to your study. If you are not sure if a list item applies to your research, read the appropriate section before selecting a response.

Materials & experimental systems

n/a	Involved in the study	
☐	☒	Antibodies
☐	☒	Eukaryotic cell lines
☒	☐	Palaeontology
☒	☐	Animals and other organisms
☐	☒	Human research participants
☒	☐	Clinical data

Methods

n/a	Involved in the study	
☒	☐	ChIP-seq
☐	☒	Flow cytometry
☒	☐	MRI-based neuroimaging

Antibodies

Antibodies used

Anti-Hu CD206 (19.2), Alexa Fluor 488, Invitrogen, 53-2069-42
Anti-Hu CC10 (E-11), Alexa Fluor 488, Santa Cruz Biotechnology, sc-365992 AF488
Anti-Hu CC10 (E-11), FITC, Santa Cruz Biotechnology, sc-365992 FITC
Anti-Hu CD326 (9C4), FITC (Ep-CAM), BioLegend, 324203
Anti-B-actin (2F1-1), Alexa Fluor 488, BioLegend, 643812
Anti-Hu Integrin a11 (2F1C10), Alexa Fluor 488, Santa Cruz Biotechnology, sc-390091 AF488
Anti-Hu Cytokeratin Pan Monoclonal Antibody (C-11), Alexa Fluor 488, Invitrogen, MA5-18156
Anti-Hu CD3 (UCHT1), Alexa Fluor 488, BioLegend, 300454
Anti-Hu CD20 (2H7), Alexa Fluor 488, BioLegend, 302316
Anti-Hu CD152 (CTLA-4) (14D3), PE, Invitrogen, 12-1529-42
Anti-Hu CD279 (PD-1) (MIH4), APC, Invitrogen, 17-9969-42
Anti-Hu Elastin (BA-4), Alexa Fluor 546, Santa Cruz Biotechnology, sc-58756 AF546
Anti-Hu CD31 (WM59), Alexa Fluor 594, BioLegend, 303126
Anti-Hu CD14 (HCD14), Alexa Fluor 594, BioLegend, 325630
Anti-Hu CD4 (RPA-T4), Alexa Fluor 594, BioLegend, 300544
Anti-Hu SP-C (H-8), Alexa Fluor 488, Santa Cruz Biotechnology, sc-518029 AF488
Anti-Hu Z0-1 (ZO1-1A12) Monoclonal Antibody, Alexa Fluor 488, Invitrogen, MA3-39100-A488

Anti-Hu Beta Tubulin Monoclonal Antibody (2 28 33), Alexa Fluor 647, Invitrogen, MA3-22600-A647
Anti-Hu Mucin 5AC (EPR16904), Alexa Fluor 555, Abcam, ab218714
Anti-Hu Mucin 5AC (45M1), Alexa Fluor 647, Santa Cruz Biotechnology, sc-21701 AF647
Anti-Hu Z0-1 Monoclonal Antibody (ZO1-1A12), Alexa Fluor 555, Invitrogen, MA3-39100-A555
Anti-Hu CD68 (KP1), Alexa Fluor 488, Abcam, ab222914
Anti-Hu CD68 (Y1/82A), Alexa Fluor 647, BioLegend, 333820
Anti-Hu CD140a (PDGFRa) (16A1), APC, BioLegend, 323512
Anti-Hu CD80 (MEM233), Alexa Fluor 647, Invitrogen, A51018
Anti-B-actin (2F1-1), Alexa Fluor 647, BioLegend, 643810
Anti-Hu CD45 (HI30), Alexa Fluor 647, BioLegend, 304056
Anti-Hu Laminin alpha 1, Alexa Fluor 647, R&D Systems, IC4187R-100UG
Anti-Hu CD56 (NCAM) (5.1H11), Alexa Fluor 647, BioLegend, 362514
Anti-Hu CD8a (C8/114B), Alexa Fluor 647, BioLegend, 372906
Anti-Hu S100A4 (NJ-4F3-D1), APC, BioLegend, 370005
Anti-Hu Z0-1 Monoclonal Antibody (ZO1-1A12), Alexa Fluor 647, Invitrogen, MA3-39100-A647
Lectin-LEA, Dylight, Invitrogen, 2384029
Anti-Hu CD14 (S18004B), Spark UV 387, BioLegend, 399215
Anti-Hu CD11b (ICRF44), Brilliant UltraViolet 805, Invitrogen, 368-0118-42
Anti-Hu CD11c (Bu15), APC/Fire 750, BioLegend, 337240
Anti-Hu CD206 (15-2), Pacific Blue, BioLegend, 321152
Anti-Hu CD1c (L161), APC, BioLegend, 331524
Anti-Hu CD141 (M80), PE, BioLegend, 344104
Anti-Hu CD103 (Ber-ACT8), Brilliant Ultraviolet 605, BioLegend, 350218
Anti-Hu MARCO (PLK-1), PE-Cyanine7, Invitrogen, 25-5447-42
Anti-Hu CD64 (10.1), Brilliant UltraViolet 510, BioLegend, 305028
Anti-Hu CD303 (201A), PerCP cyanine5.5, BioLegend, 354210
Anti-Hu CCR7 (3D12), Brilliant UltraViolet 661, BD Biosciences, 376-1979-42
Anti-Hu CD14 (S18004B), Spark UV 387, BioLegend, 399215
Anti-Hu CD14 (M5E2), PE, BioLegend, 301850
Anti-Hu CD68 (Y1/82A), Alexa Fluor 488, BioLegend, 333812
Anti-Hu CD206 (19.2), PerCP-eFluor 710, Invitrogen, 46-2069-42
Anti-Hu CD11c (3.9), APC-eFluor 780, Invitrogen, 47-0116-42
Anti-Hu CD45 (HI30), Spark UV 387, BioLegend, 304086
Anti-Hu CD20 (2H7), Brilliant UltraViolet 737, Invitrogen, 367-0209-42
Anti-Hu CD56 (NCAM) (TULY56), Super Bright 600, Invitrogen, 63-0566-42
Anti-Hu CD15 (W6D3), Brilliant Violet 711, BioLegend, 323049
Anti-Hu CD11b (ICRF44), APC, Invitrogen, 17-0118-42
Anti-Hu CD138 (MI15), FITC, BioLegend, 356507
Anti-Hu CD31 (WM59), PerCP/Cyanine5.5, BioLegend, 303132
Anti-Vimentin (O91D3), Alexa Fluor 594, BioLegend, 677804
Anti-Hu CD68 (Y1/82A), PE/Cyanine7, BioLegend, 333816
Anti-Cytokeratin (pan reactive) (C-11), Alexa Fluor 647, BioLegend, 628604
Anti-Hu CD326 (Ep-CAM) (9C4), Alexa Fluor 700, BioLegend, 324244
Anti-Hu CD8a (RPA-T8), APC-eFluor 780, Invitrogen, 47-0088-42
Anti-Hu CD86 (IT2.2), Super Bright 600, Invitrogen, 63-0869-42
Anti-Hu HLA-DR (LN3), Super Bright 780, Invitrogen, 78-9956-42
Zombie UV Fixable Viability, BioLegend, 423108
Anti-Hu CD15 (MMA), PerCP-eFluor 710, Invitrogen, 46-0158-42
Anti-Hu CD3 (OKT3), FITC, Invitrogen, 11-0037-42
Anti-Hu CD4 (RPA-T4), Super Bright 780, Invitrogen, 78-0049-42
Anti-Hu CD14 (M5E2), PE, BioLegend, 301850
Anti-Hu CD20 (2H7), APC, Invitrogen, 17-0209-42
Anti-Hu CD45 (2D1), Super Bright 780, Invitrogen, 78-9459-42
Anti-Hu CD133 (Prominin-1) (TMP4), PerCP-eFluor 710, Invitrogen, 46-1338-42
Anti-Hu CD14 (61D3), APC, Invitrogen, 17-0149-42
LIVE/DEAD Blue, Invitrogen, L23105
Anti-Hu CD25 (BC96), Super Bright 780, Invitrogen, 78-0259-42
Anti-Hu CD69 (FN50), RB705, BD Biosciences, 570278
Anti-Hu NKp44 (p44-8), Brilliant UltraViolet 615, BD Biosciences, 752353
Anti-Hu HLA-DR (G46-6), Brilliant UltraViolet 737, BD Biosciences, 568351
Anti-Hu CD56 (HCD56), Brilliant Violet570, BioLegend, 318330
Anti-Hu CD8 (SK1), PE-Fire700, BioLegend, 344766
Anti-Hu CD16 (3G8), Brilliant Violet 650, BD Biosciences, 563691
Anti-Hu NKG2D (1D11), PE-Cyanine5, BioLegend, 320844
Anti-Hu CD4 (RPA-T4), PE-Cyanine7, BioLegend, 300512
Anti-Hu CD3 (SK7), Brilliant UltraViolet 395, BD Biosciences, 565983
Anti-Hu CD45 (HI30), Brilliant UltraViolet 496, BD Biosciences, 569101
Anti-Hu IFN-γ (B27), V500, BD Biosciences, 561980
Anti-Hu Granzyme B (QA18A28), PerCP, BioLegend, 396416

Validation

All antibodies and viability stains were validated by their respective manufacturers and utilized at the manufacturer's recommended dilution, in the event of no clear recommendation a dilution of 1:200 was used. Antibodies were also validated for use using positive and negative control samples using dilutions and conditions based on the manufacturer's recommendation or our previous experience. The absence of a primary antibody was used for validating staining for all antibodies tested for imaging.

For flow, all antibodies were titrated using cells positive for the antibody marker.

Eukaryotic cell lines

Policy information about cell lines

Cell line source(s)	Primary small airway epithelial cells (CC-2547), primary normal human lung fibroblasts (CC-2512), and primary human umbilical vein endothelial cells (C2519A) were acquired from Lonza. All immune cells were isolated from whole blood from donors.
Authentication	Macrophage and dendritic cell differentiation was confirmed via flow cytometry analysis. HUVECs were confirmed to express endothelial marker CD31 and Lectin via confocal microscopy and flow cytometry, SAECs were confirmed to express epithelial cell markers EpCAM and E-cadherin, and fibroblasts were confirmed via morphology.
Mycoplasma contamination	All cells acquired from Lonza tested negative for mycoplasma prior to purchasing. Immune cells were not tested for mycoplasma contamination.
Commonly misidentified lines (See ICLAC register)	None

Human research participants

Policy information about studies involving human research participants

Population characteristics	Donor Characteristic: M, 44
Recruitment	Donor was recruited under Emory protocol, and de-identified blood samples were provided to us.
Ethics oversight	Sample from human donors were acquired in compliance with Emory University's institutional review board-approved protocol "Phlebotomy of Healthy Adults for the Purpose of Evaluation and Validation of Immune Response Assays" (IRB00045821)

Note that full information on the approval of the study protocol must also be provided in the manuscript.

Flow Cytometry

Plots

Confirm that:

☒ The axis labels state the marker and fluorochrome used (e.g. CD4-FITC).

☒ The axis scales are clearly visible. Include numbers along axes only for bottom left plot of group (a 'group' is an analysis of identical markers).

☒ All plots are contour plots with outliers or pseudocolor plots.

☒ A numerical value for number of cells or percentage (with statistics) is provided.

Methodology

Sample preparation	Once cells were harvested from the lung-on-chip devices, as described above, they were rinsed 3 times with 1X PBS to remove any residual cell culture media. They were then pelleted via centrifugation (300 xg, 5 min) and resuspended in a Zombie live/dead stain (BioLegend) at a 1:500 dilution in 1X PBS. The cells were allowed to incubate for 15 minutes at RT, protected from light. After incubation, 2 µL of FC block was added to each sample and allowed to incubate for an additional 15 minutes, on ice. The relevant surface-staining antibodies were then added to each sample, and they were incubated at 4 °C, protected from light, for 30 minutes. For panels that included more than 2 BD Horizon Brilliant dyes, BD Horizon Brilliant Stain Buffer plus was added to the staining solution at 10 µL per sample. The samples were then centrifuged at 300 xg for 5 minutes and resuspended in 250 µL of FACS buffer. If intracellular staining was to be done, the samples were then pelleted (300 xg, 5 min) and resuspended in 250 µL of BD Cytofix/Cytoperm (BD Biosciences) fixation and permeabilization buffer and incubated for 20 minutes at RT, protected from light. After incubation, the samples were pelleted (300 xg, 5 min) and resuspended in 100 µL of BD Perm/Wash Buffer. The relevant intracellular staining antibodies were added to the samples and allowed to incubate for 30 minutes on ice, protected from light. The samples were then washed 3X with Perm/Wash buffer and centrifuged for a final time (300 xg, 5 min) and resuspended in 250 µL Perm/Wash buffer before being analyzed on either a 3-laser Beckman Coulter CytoFLEX or a 5-laser Cytek Aurora. All antibodies were used at a 1:200 dilution.
Instrument	Flow cytometry data was acquired on either a 3-laser Beckman Coulter CytoFLEX or a 5-Laser Cytek® Aurora System using CytExpert or SpectroFlo® software, respectively.
Software	All flow cytometry data was analyzed using FlowJo using the FlowJo V10 software.
Cell population abundance	All data was performed on samples with at least 10,000 cells.

Gating strategy

Gating was established using negative controls and FMO controls to identify the positive population. For samples with multiple stains, single stain controls were used to establish compensation.

☒ Tick this box to confirm that a figure exemplifying the gating strategy is provided in the Supplementary Information.
